# Supplementary material for: Sld3CBD–Cdc45 structural insights into Cdc45 recruitment for CMG complex formation during DNA replication
Source: eLife. 2025 Sep 8;13:RP101717. doi: 10.7554/eLife.101717 (PMC12416888; doi:10.7554/eLife.101717)
Supplement: Figure 1—figure supplement 2—source data 1. [file elife-101717-fig1-figsupp2-data1.pdf]

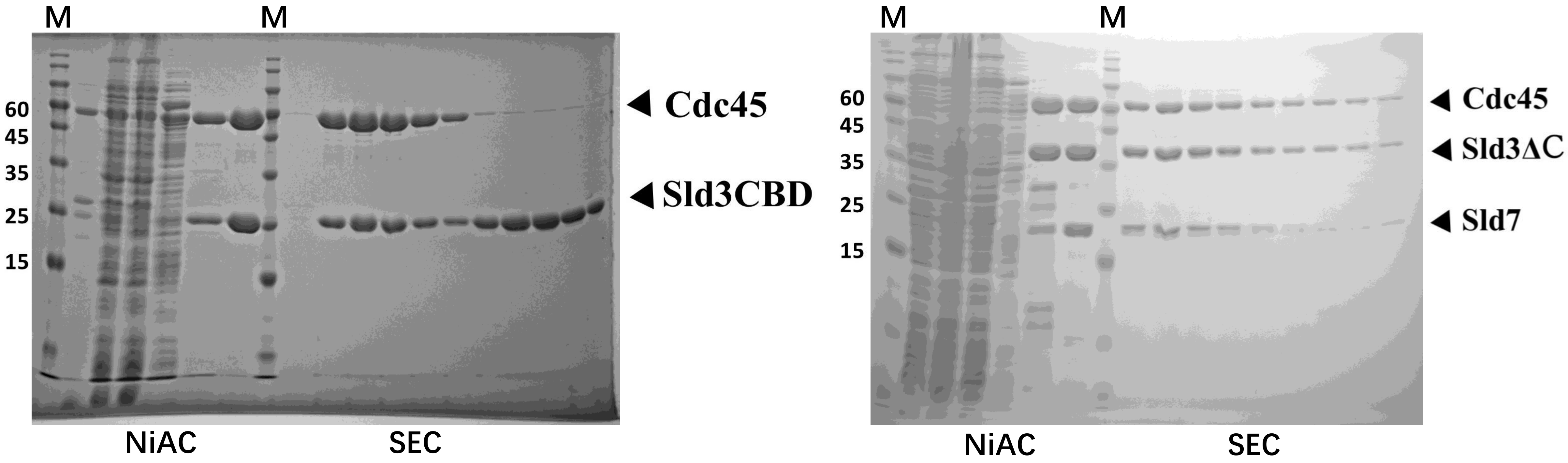

Figure 1, Source Data 1. Original SDS-PAGE corresponding to Figure1-figure supplement2 A and B. The left part is the Ni-AC purification before SEC.
