# Supplementary material for: Sld3CBD–Cdc45 structural insights into Cdc45 recruitment for CMG complex formation during DNA replication
Source: eLife. 2025 Sep 8;13:RP101717. doi: 10.7554/eLife.101717 (PMC12416888; doi:10.7554/eLife.101717)
Supplement: Figure 2—source data 1. [file elife-101717-fig2-data1.pdf]

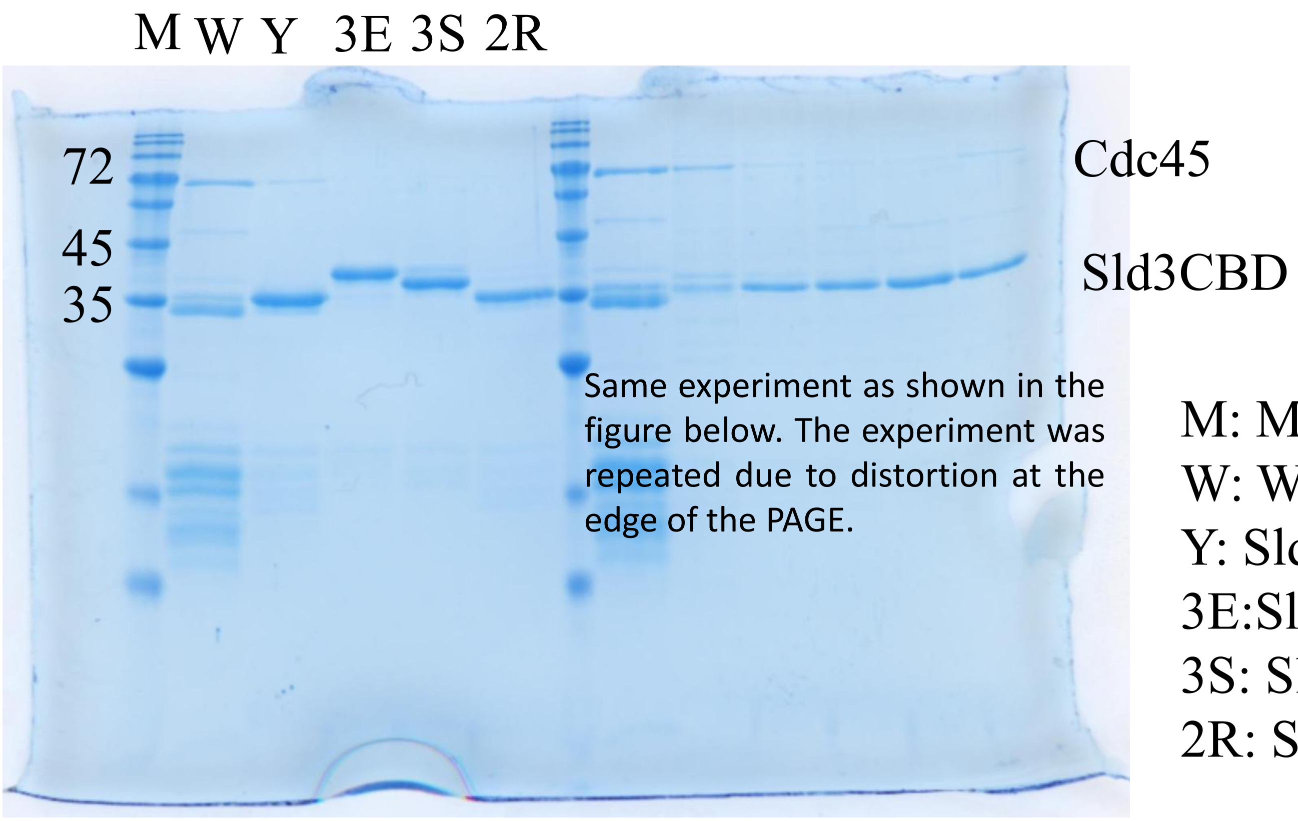

M: Marker
W: Wild type
Y: Sld3-I352Y
3E:Sld3-I352E/I355E/L356E
3S: Sld3-I352S/I355S/L356S
2R: Sld3-D344R/D348R

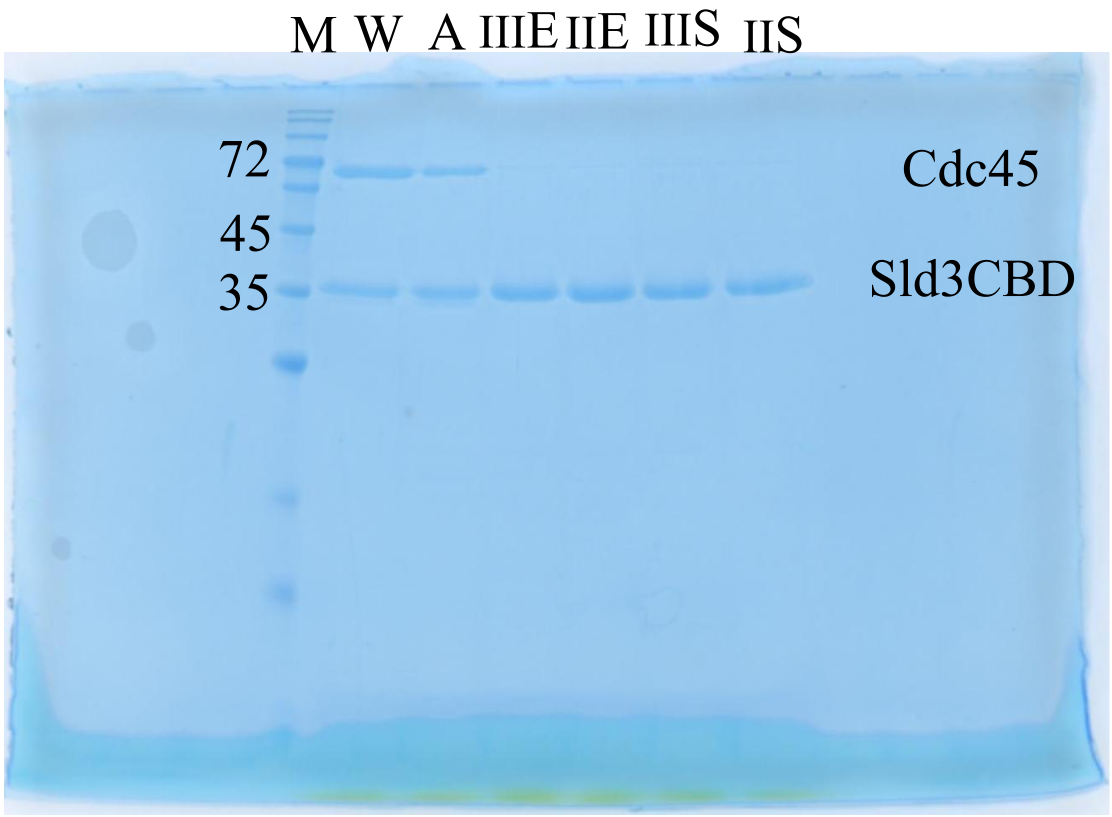

M: Marker
W: Wild type
A: Cdc45 R523A
IIIE: Cdc45-L522E/L527E/V529E
IIE: Cdc45-L637E/L641E
IIIS: Cdc45-L522S/L527S/V529S
IIS: Cdc45-L637S/L641S

Figure 2, Source Data 1. Original SDS-PAGE corresponding to Figure 3 A and B.
