# Supplementary figures and images for: Sld3CBD–Cdc45 structural insights into Cdc45 recruitment for CMG complex formation during DNA replication
Source: eLife. 2025 Sep 8;13:RP101717. doi: 10.7554/eLife.101717 (PMC12416888; doi:10.7554/eLife.101717)

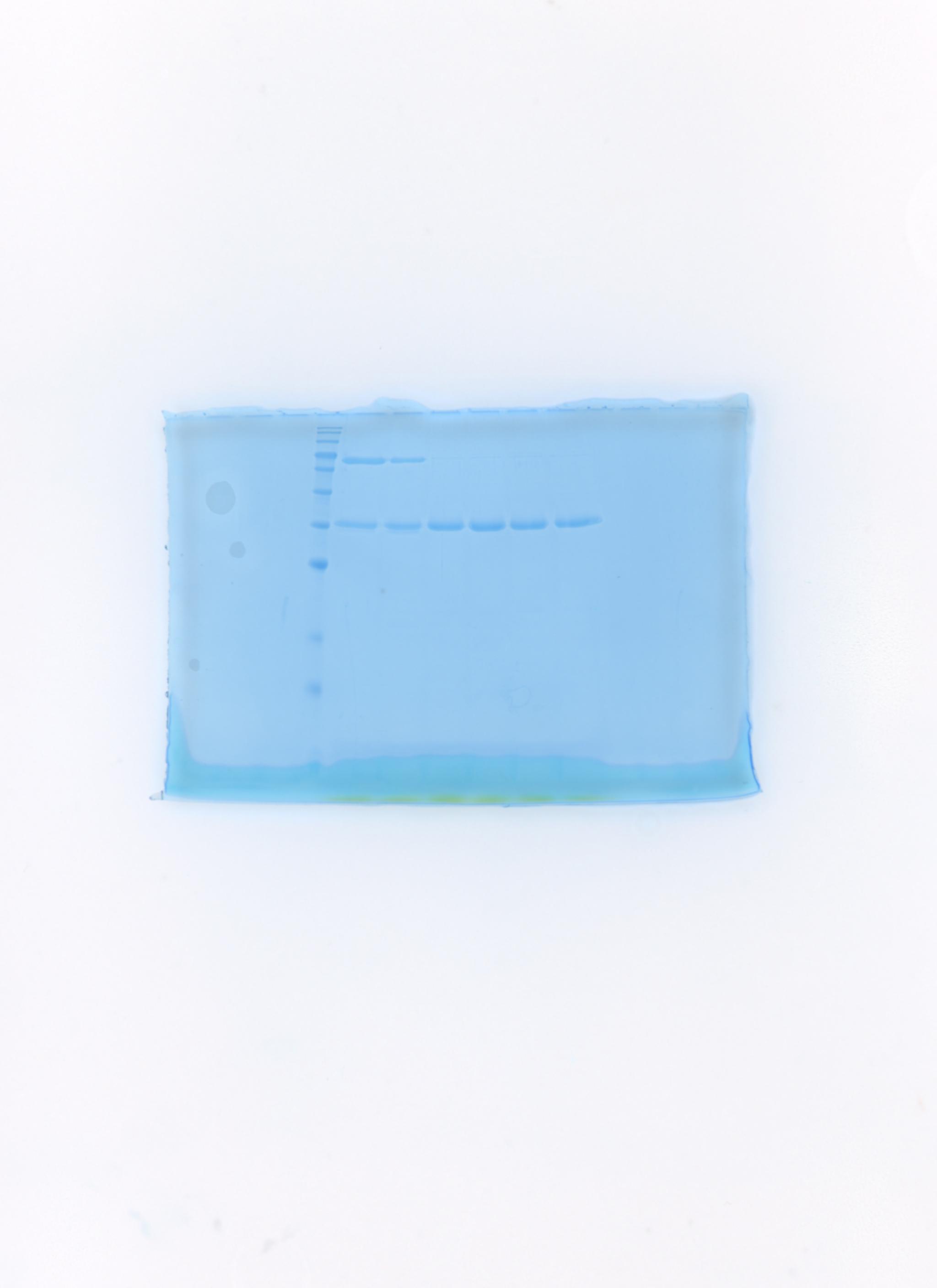

Supplement: Figure 2—source data 2. [file elife-101717-fig2-data2.zip › Sld3Cdc45 mutant re.jpg]

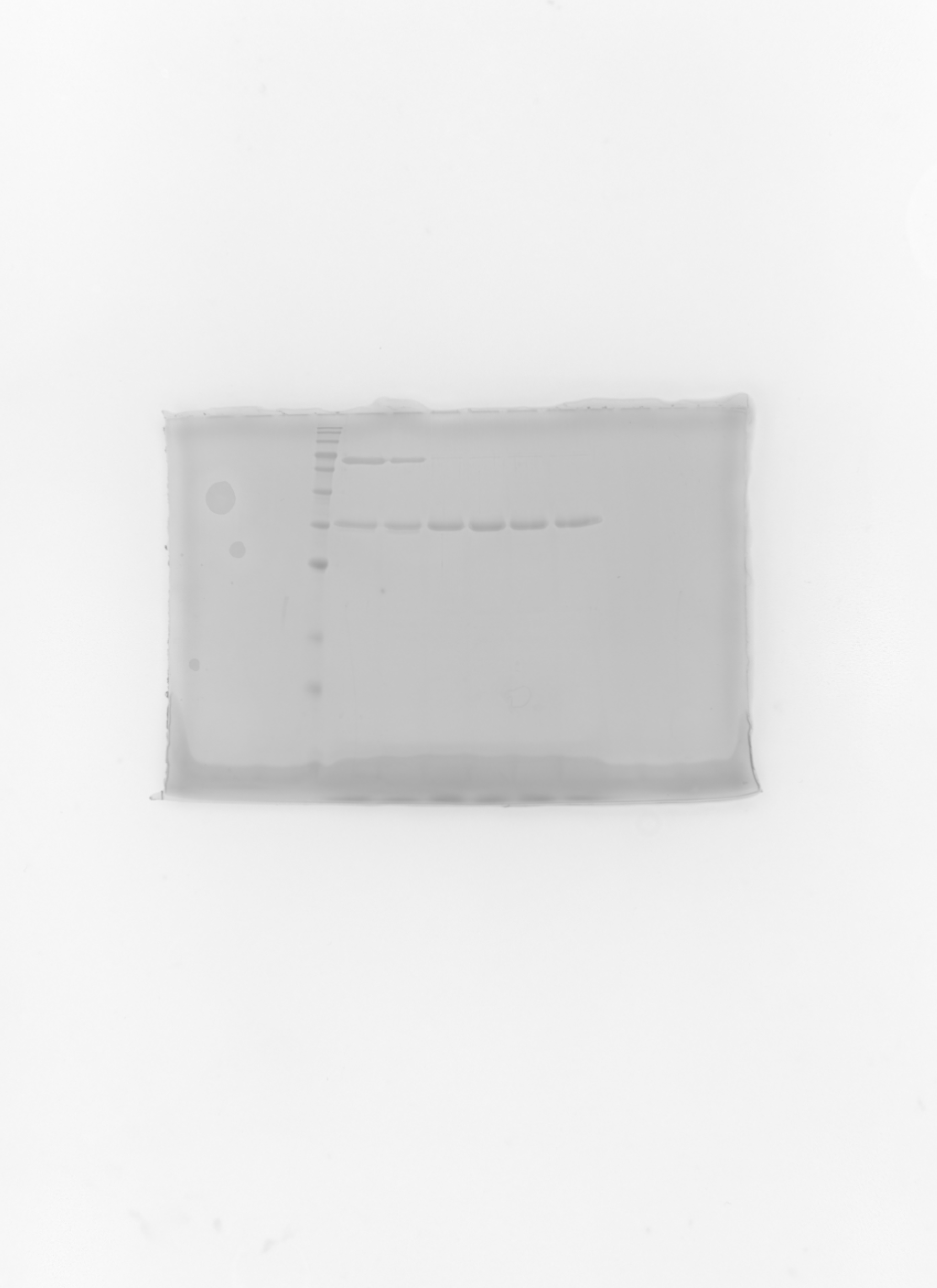

Supplement: Figure 2—source data 2. [file elife-101717-fig2-data2.zip › Sld3Cdc45 mutant re.tif]

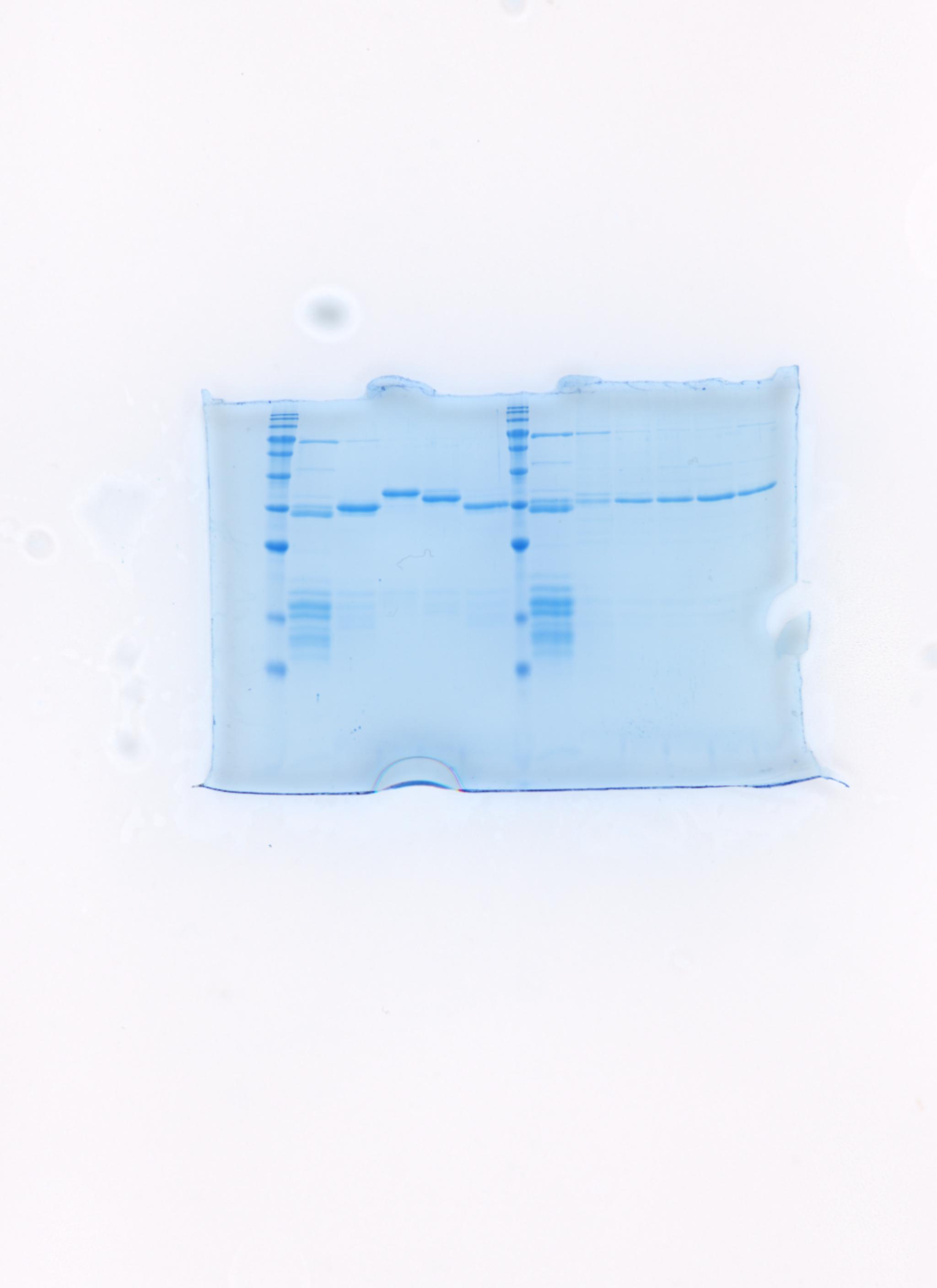

Supplement: Figure 2—source data 2. [file elife-101717-fig2-data2.zip › Sld3Cdc45 mutant.jpg]

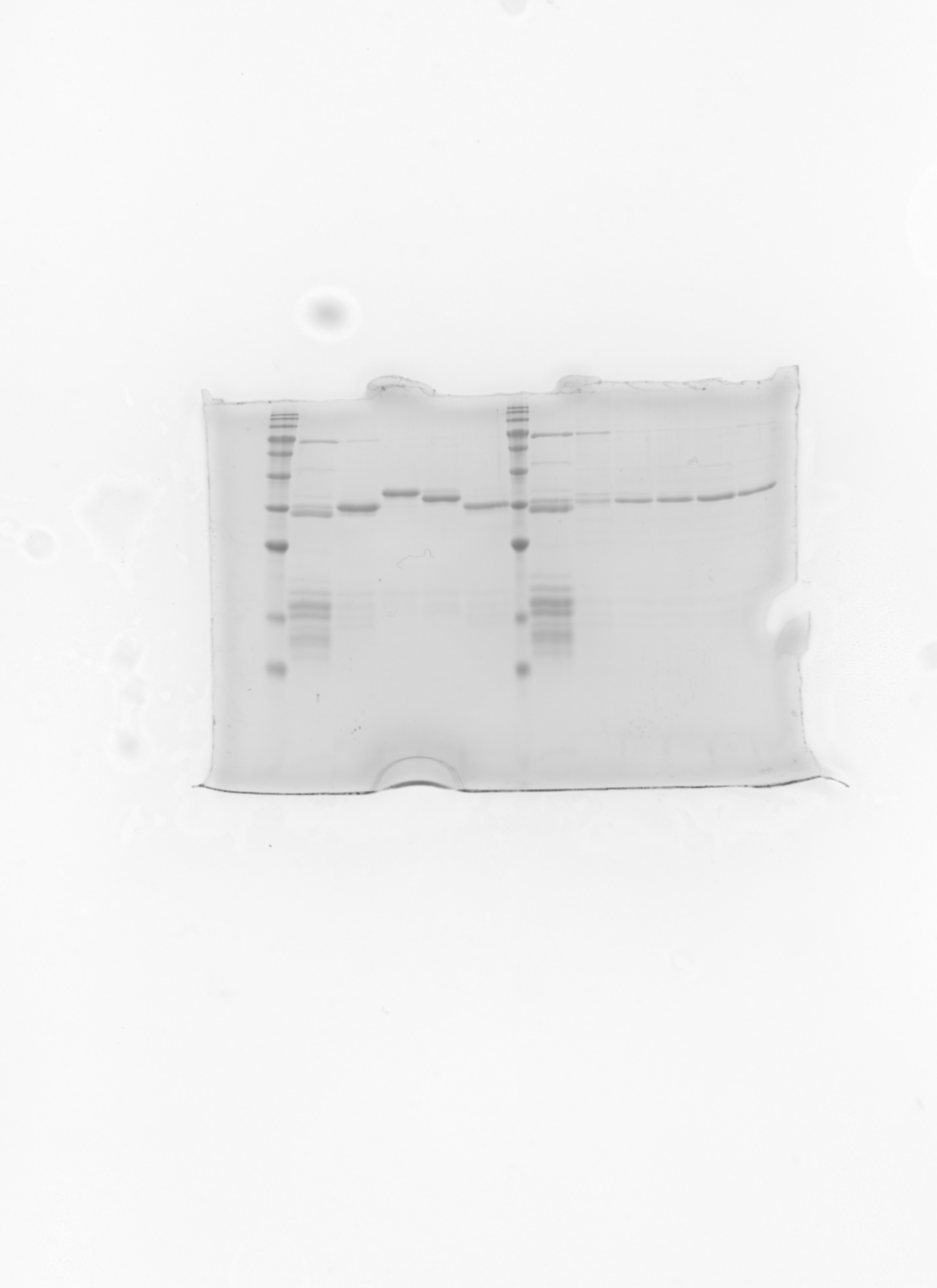

Supplement: Figure 2—source data 2. [file elife-101717-fig2-data2.zip › Sld3Cdc45 mutant.tif]

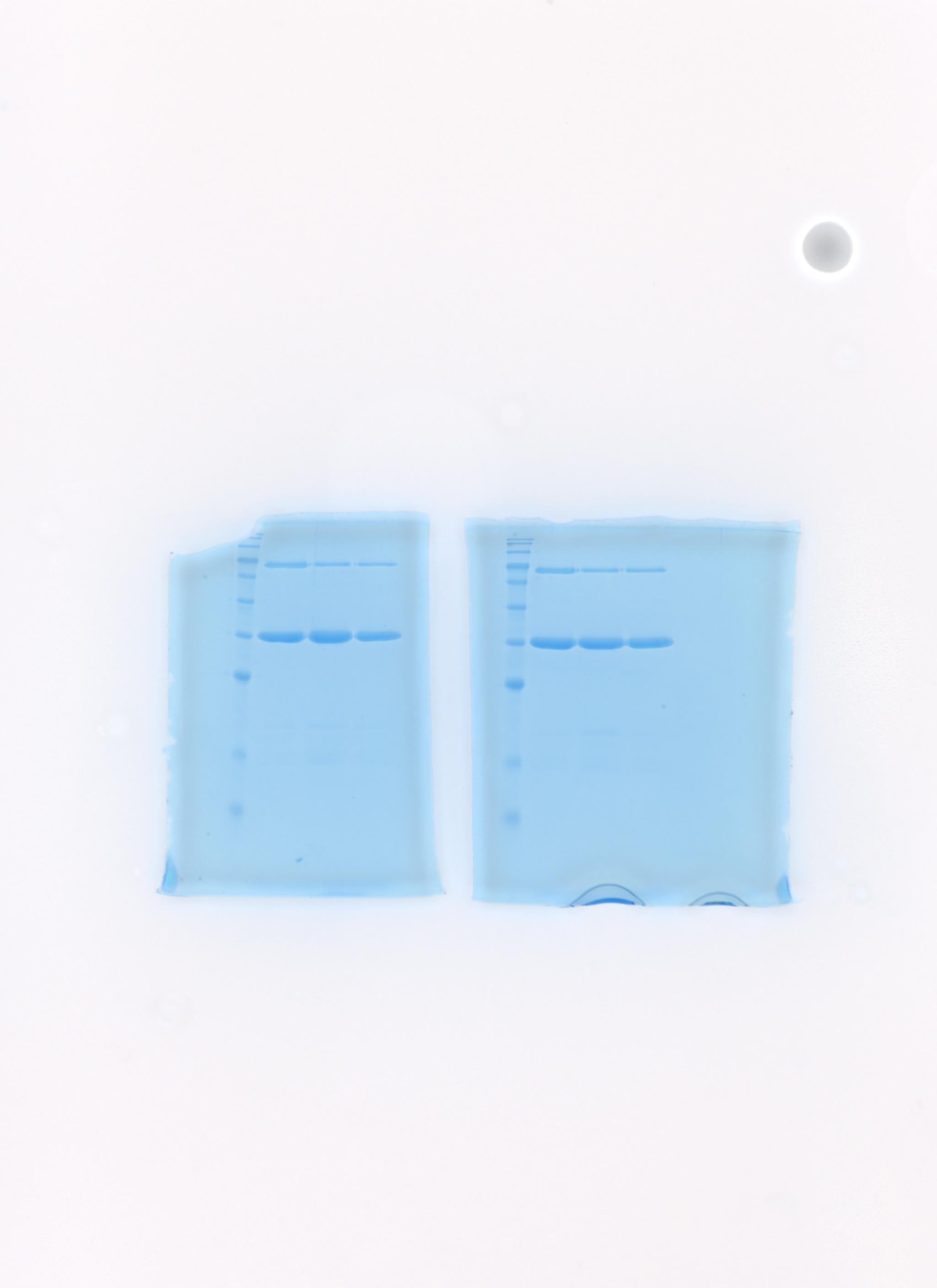

Supplement: Figure 2—figure supplement 3—source data 2. [file elife-101717-fig2-figsupp3-data2.zip › mutant 2.jpg]

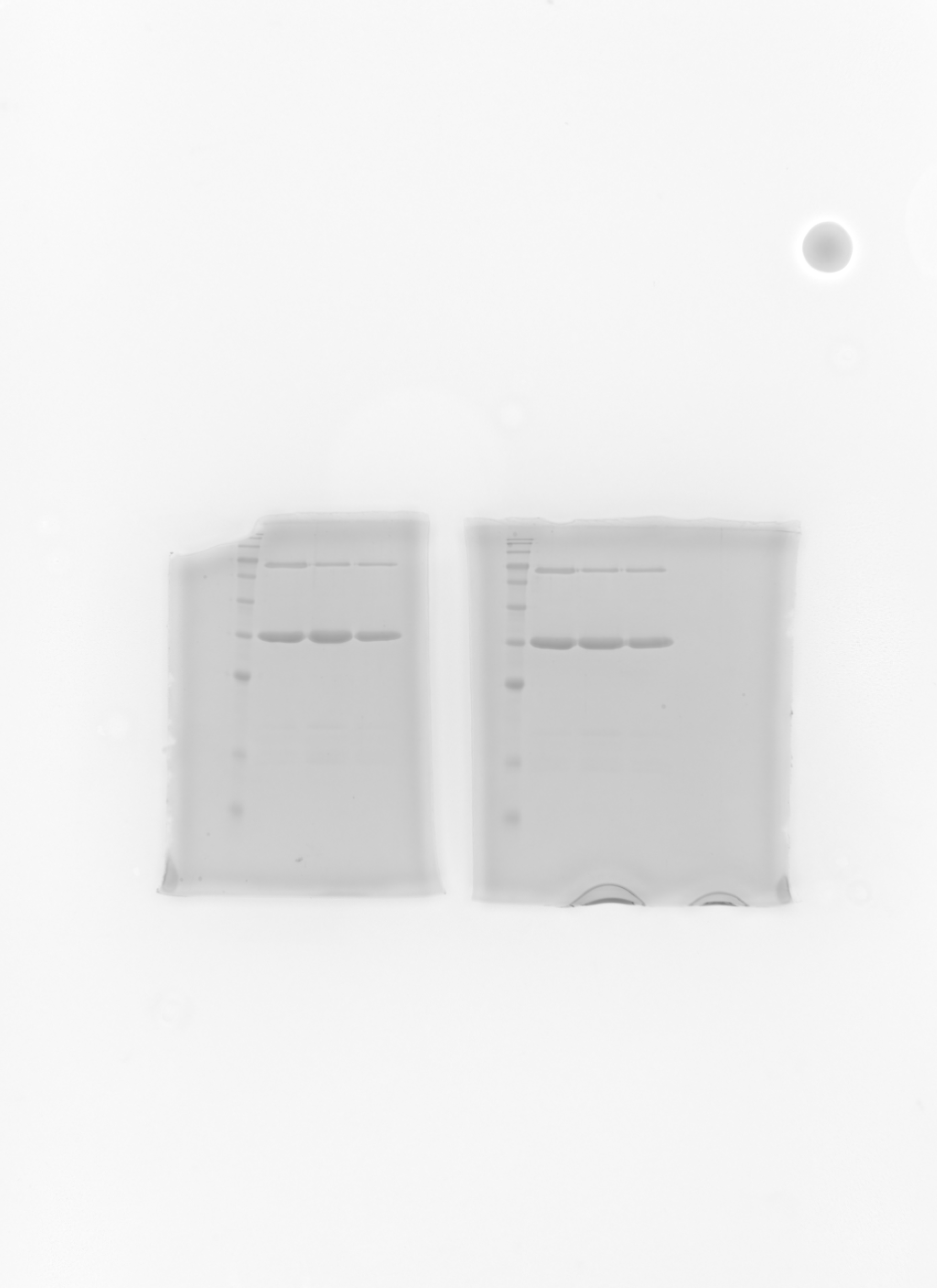

Supplement: Figure 2—figure supplement 3—source data 2. [file elife-101717-fig2-figsupp3-data2.zip › mutant 2.tif]

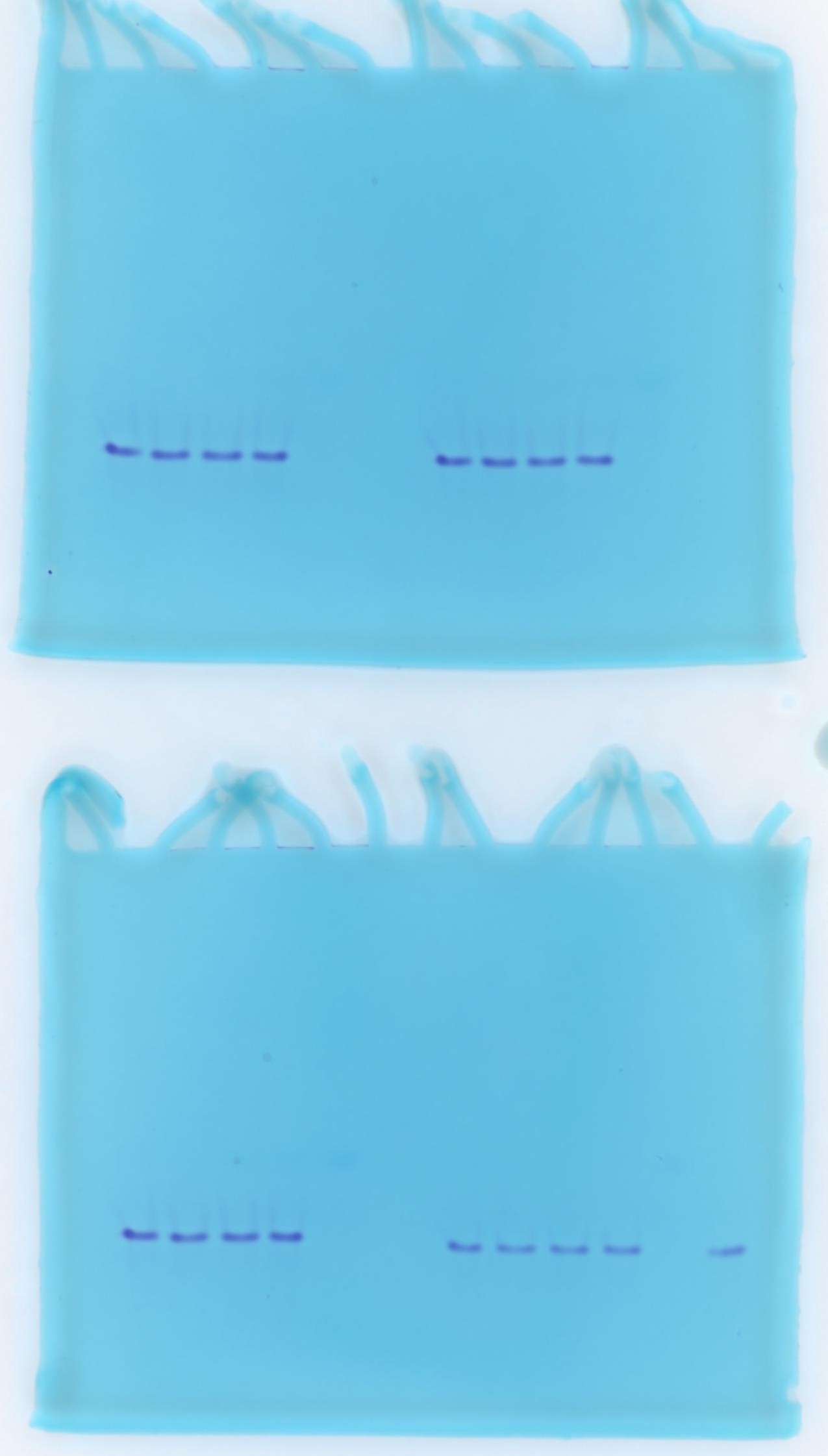

Supplement: Figure 4—source data 2. [file elife-101717-fig4-data2.zip › 1-3-1 NC.jpg]

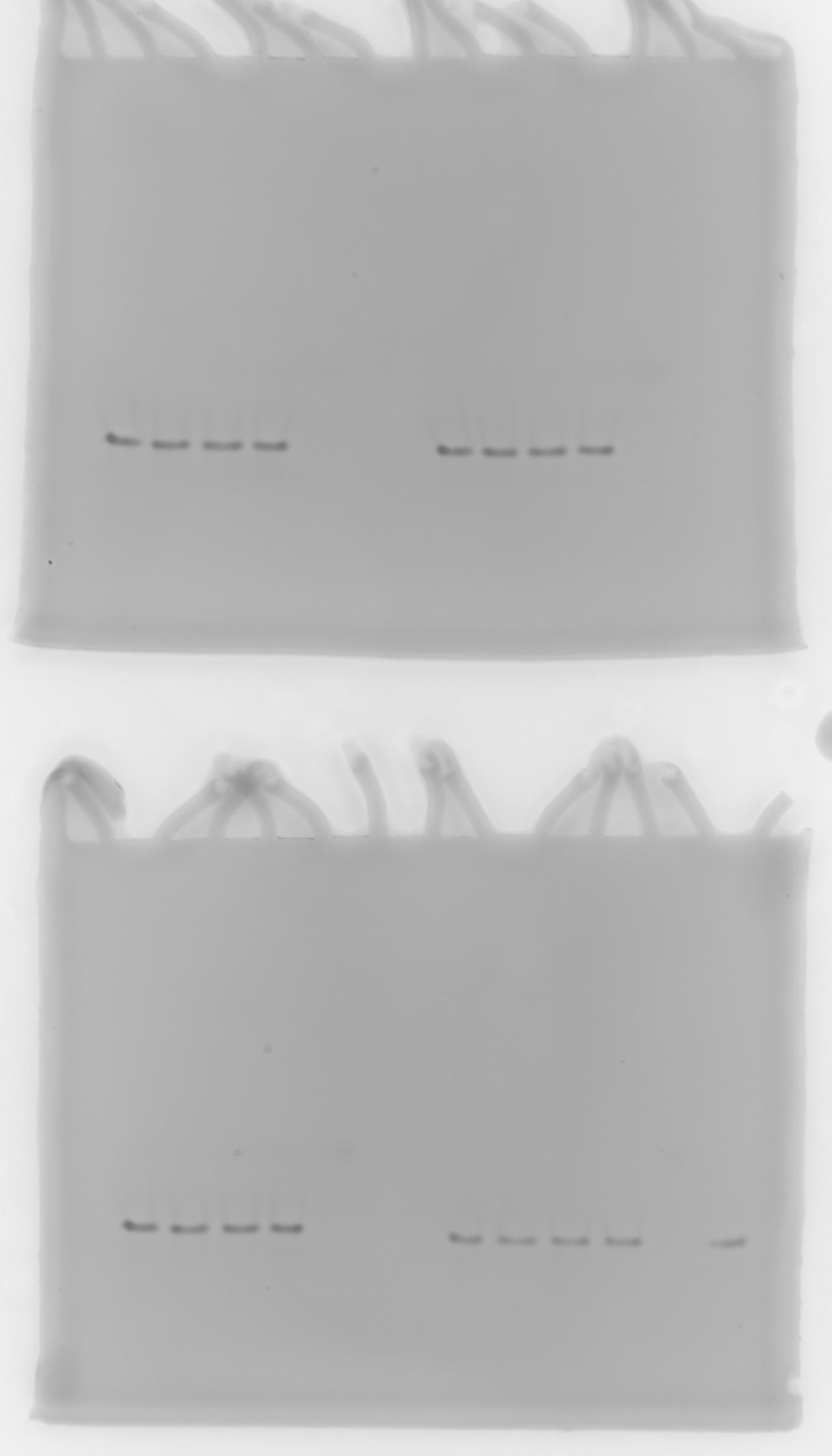

Supplement: Figure 4—source data 2. [file elife-101717-fig4-data2.zip › 1-3-1 NC.tif]

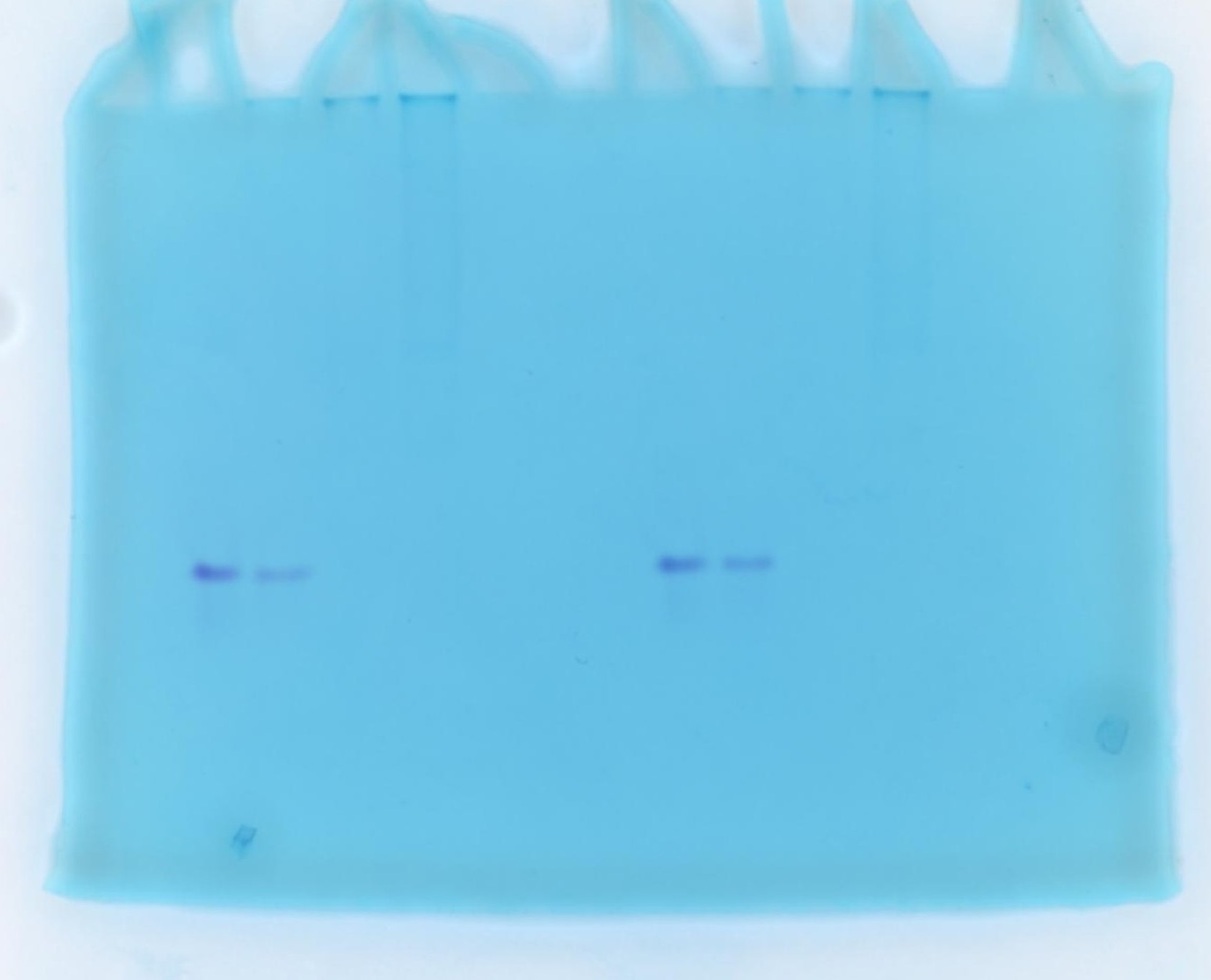

Supplement: Figure 4—source data 2. [file elife-101717-fig4-data2.zip › sld3 1-5-1,2.jpg]

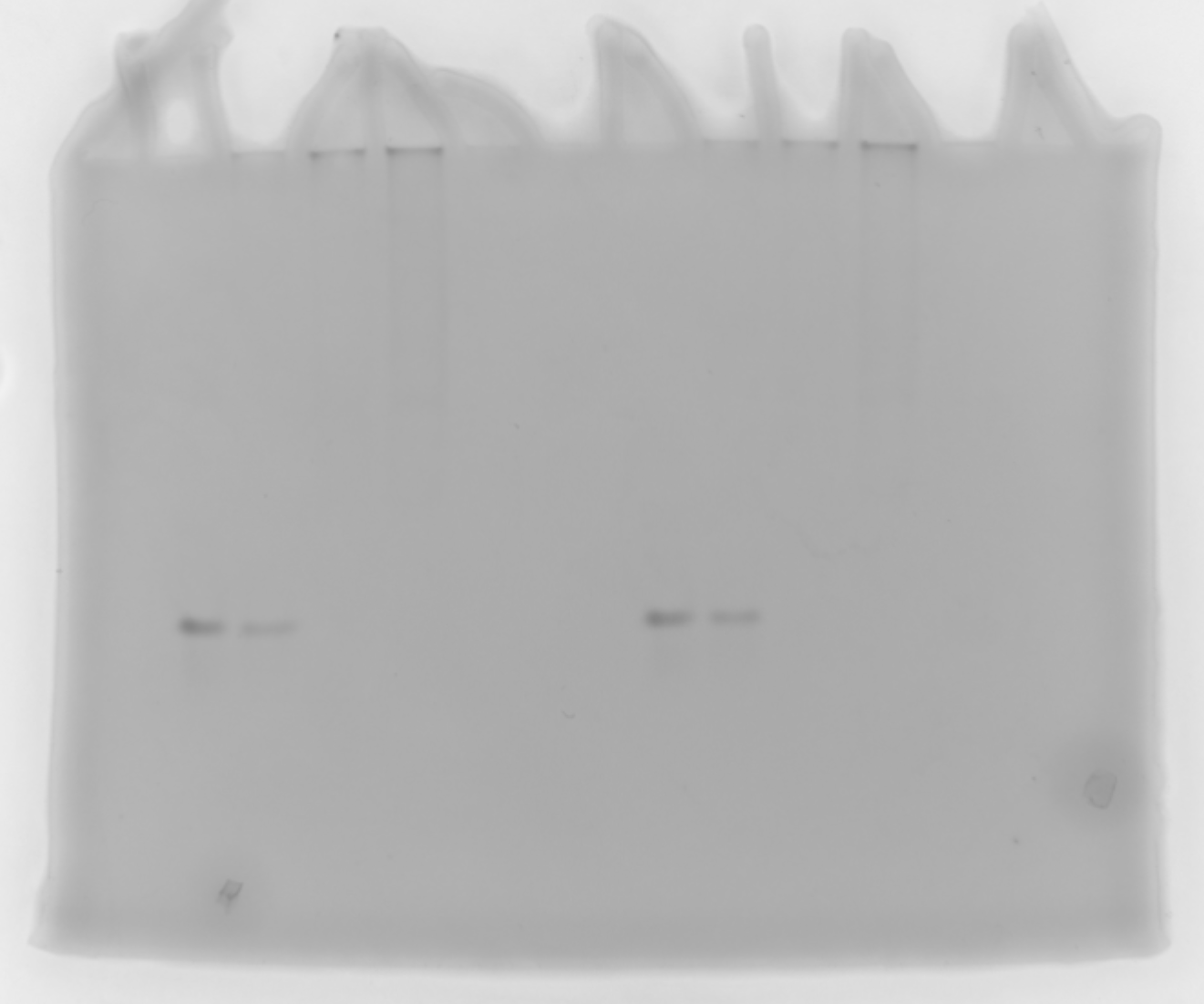

Supplement: Figure 4—source data 2. [file elife-101717-fig4-data2.zip › sld3 1-5-1,2.tif]

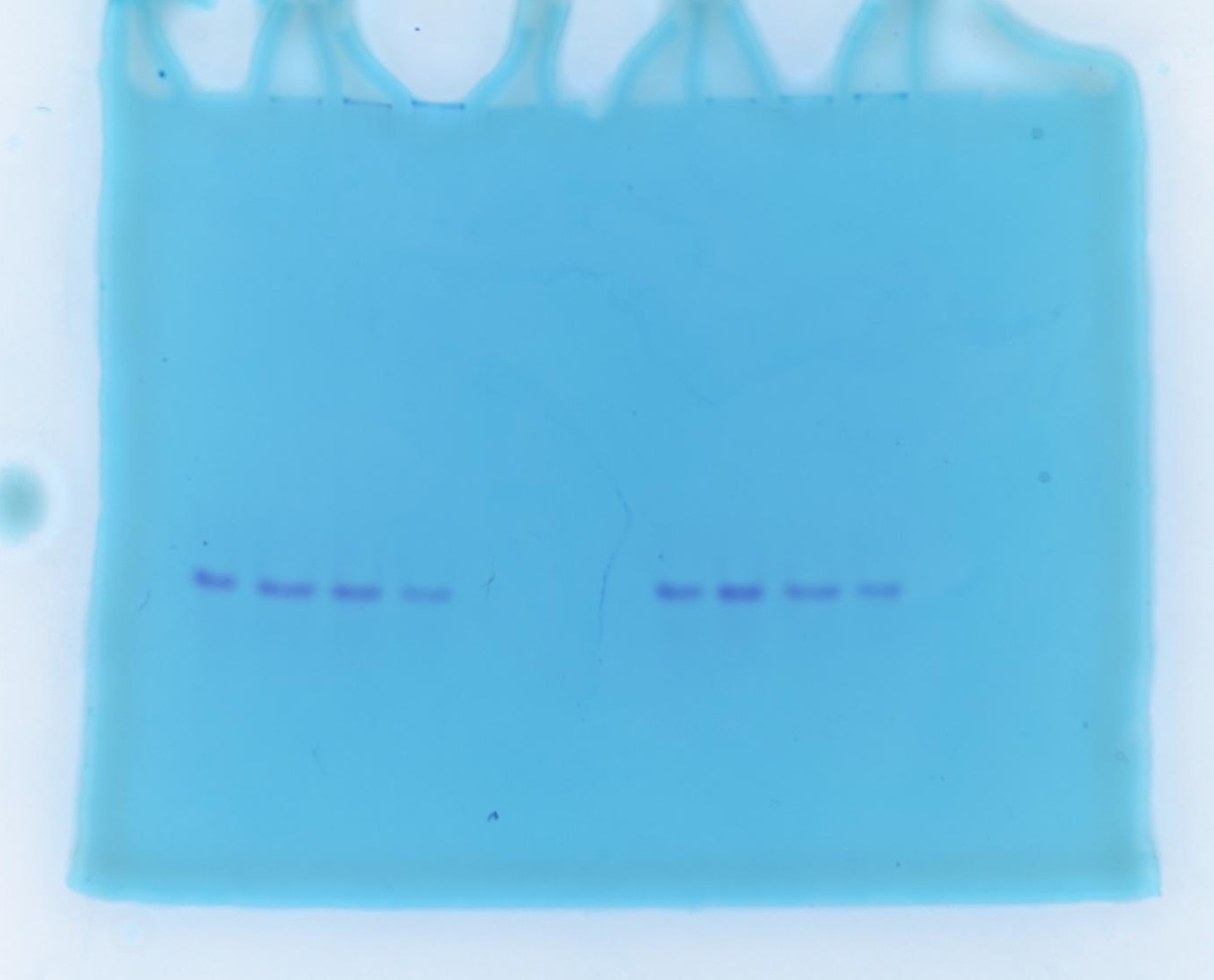

Supplement: Figure 4—source data 2. [file elife-101717-fig4-data2.zip › sld3CBDcdc45 1-5-1,2.jpg]

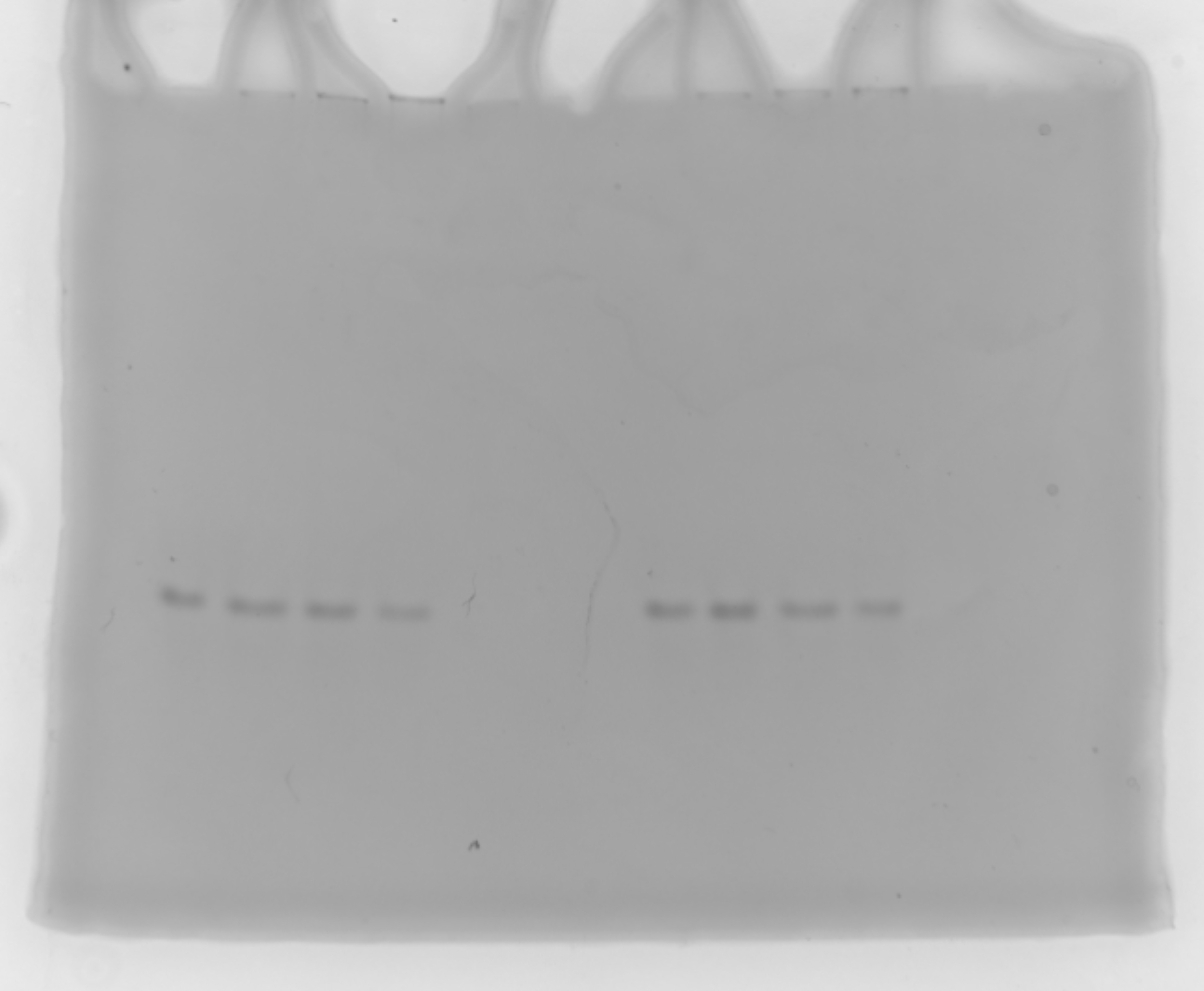

Supplement: Figure 4—source data 2. [file elife-101717-fig4-data2.zip › sld3CBDcdc45 1-5-1,2.tif]

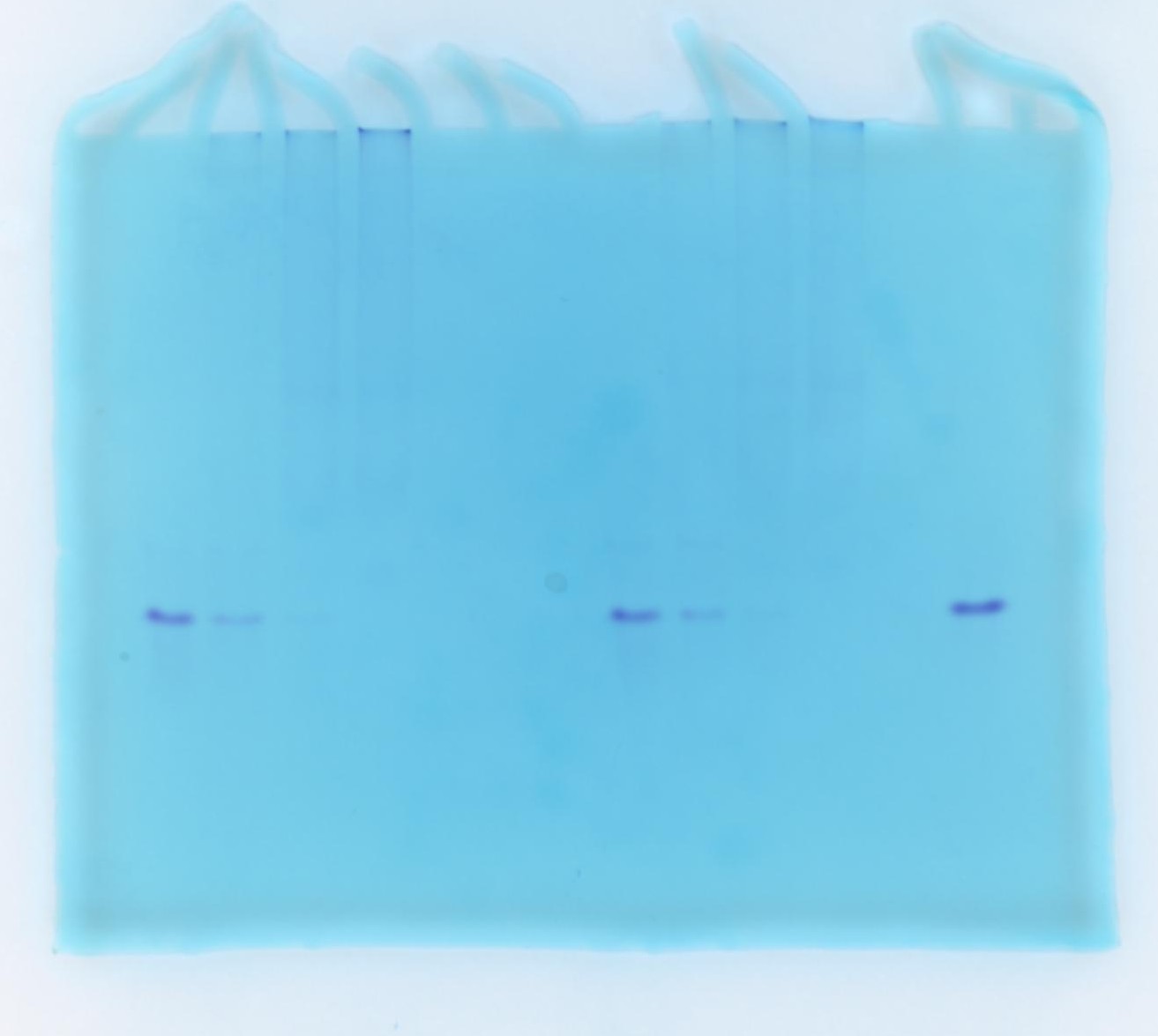

Supplement: Figure 4—source data 2. [file elife-101717-fig4-data2.zip › sld3sld7 1-5-1,2.jpg]

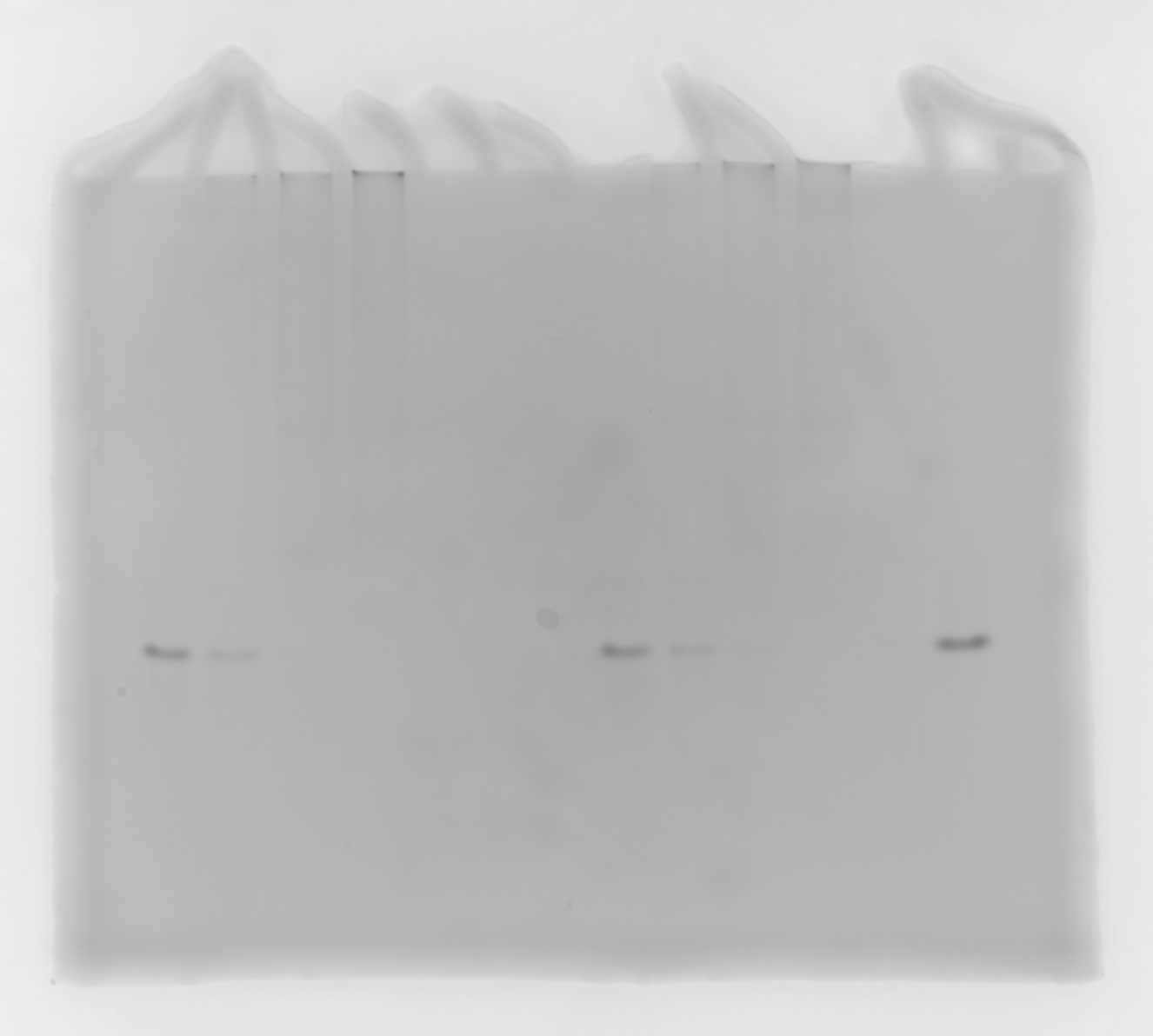

Supplement: Figure 4—source data 2. [file elife-101717-fig4-data2.zip › sld3sld7 1-5-1,2.tif]

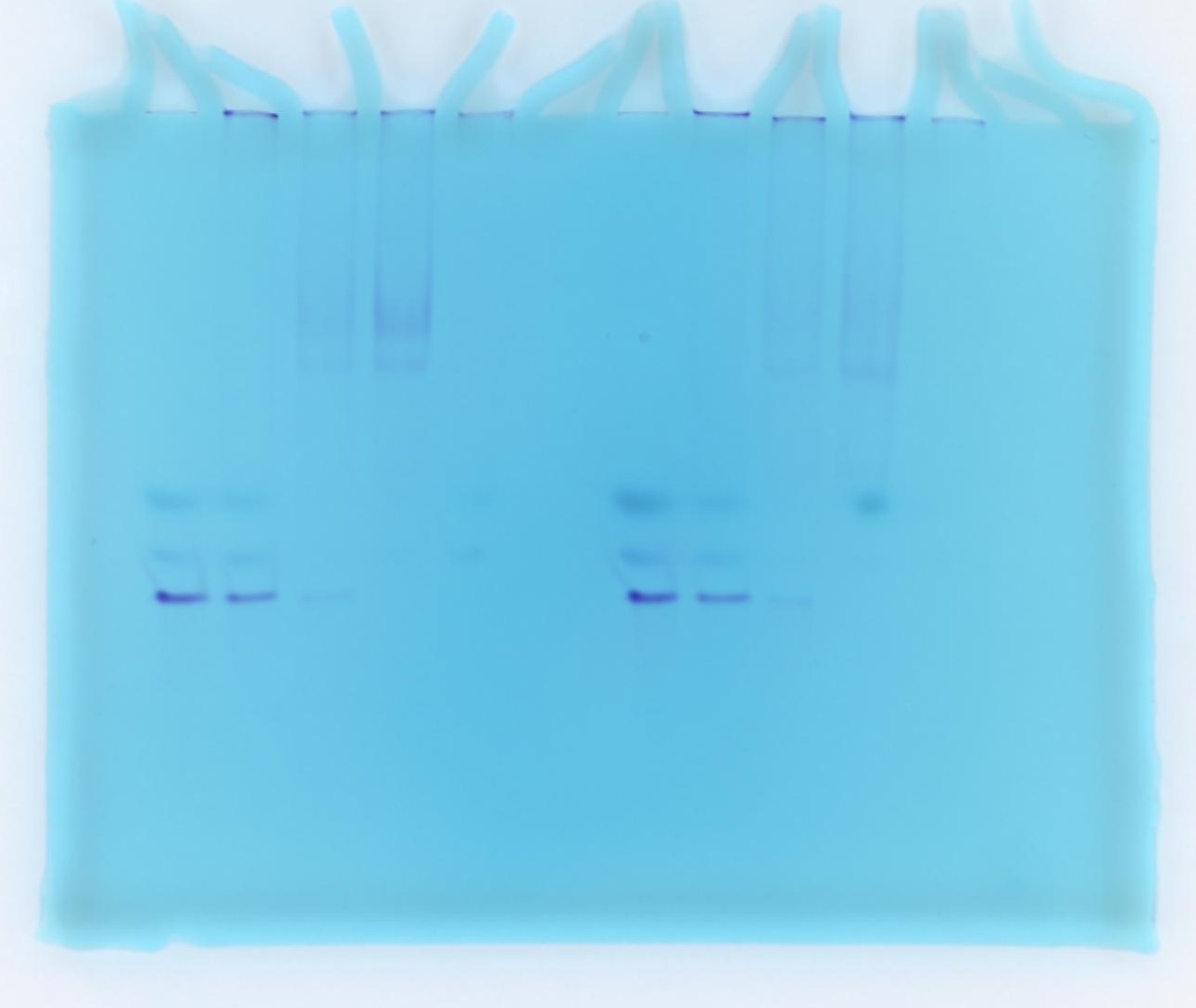

Supplement: Figure 4—source data 2. [file elife-101717-fig4-data2.zip › Sld3Sld7Cdc45 1-5-1,2.jpg]

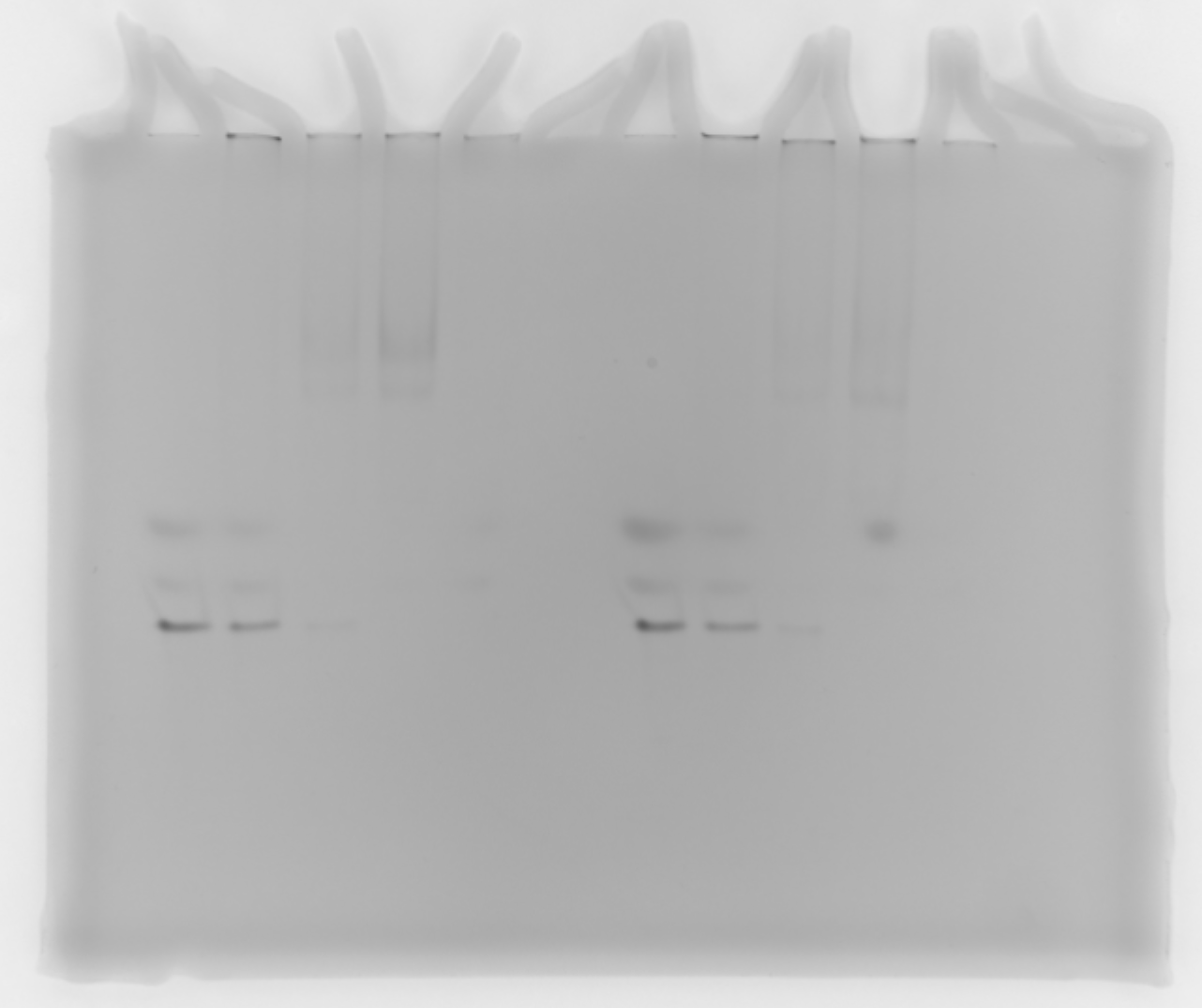

Supplement: Figure 4—source data 2. [file elife-101717-fig4-data2.zip › Sld3Sld7Cdc45 1-5-1,2.tif]

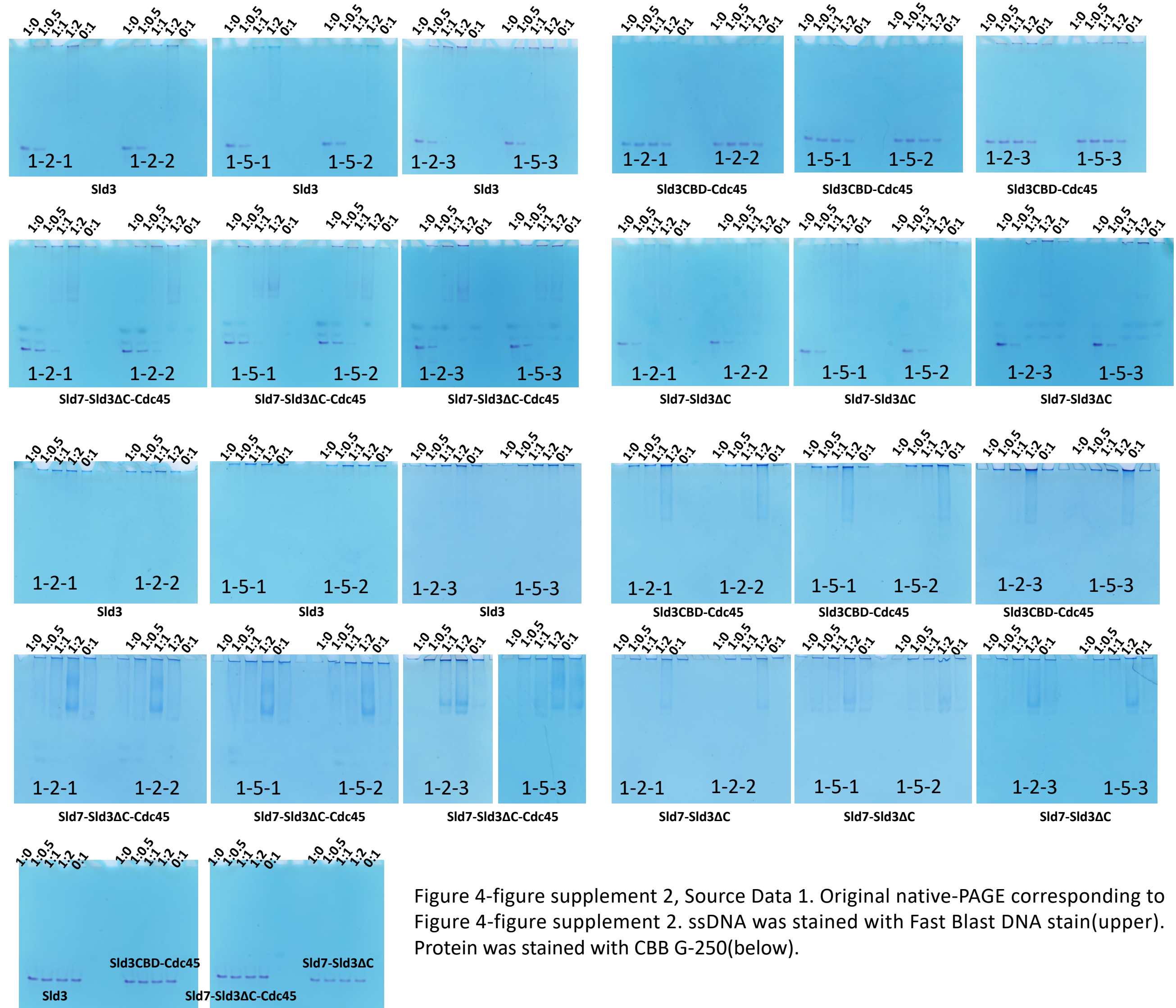

Supplement: Figure 4—figure supplement 2—source data 1. [file elife-101717-fig4-figsupp2-data1.pdf]

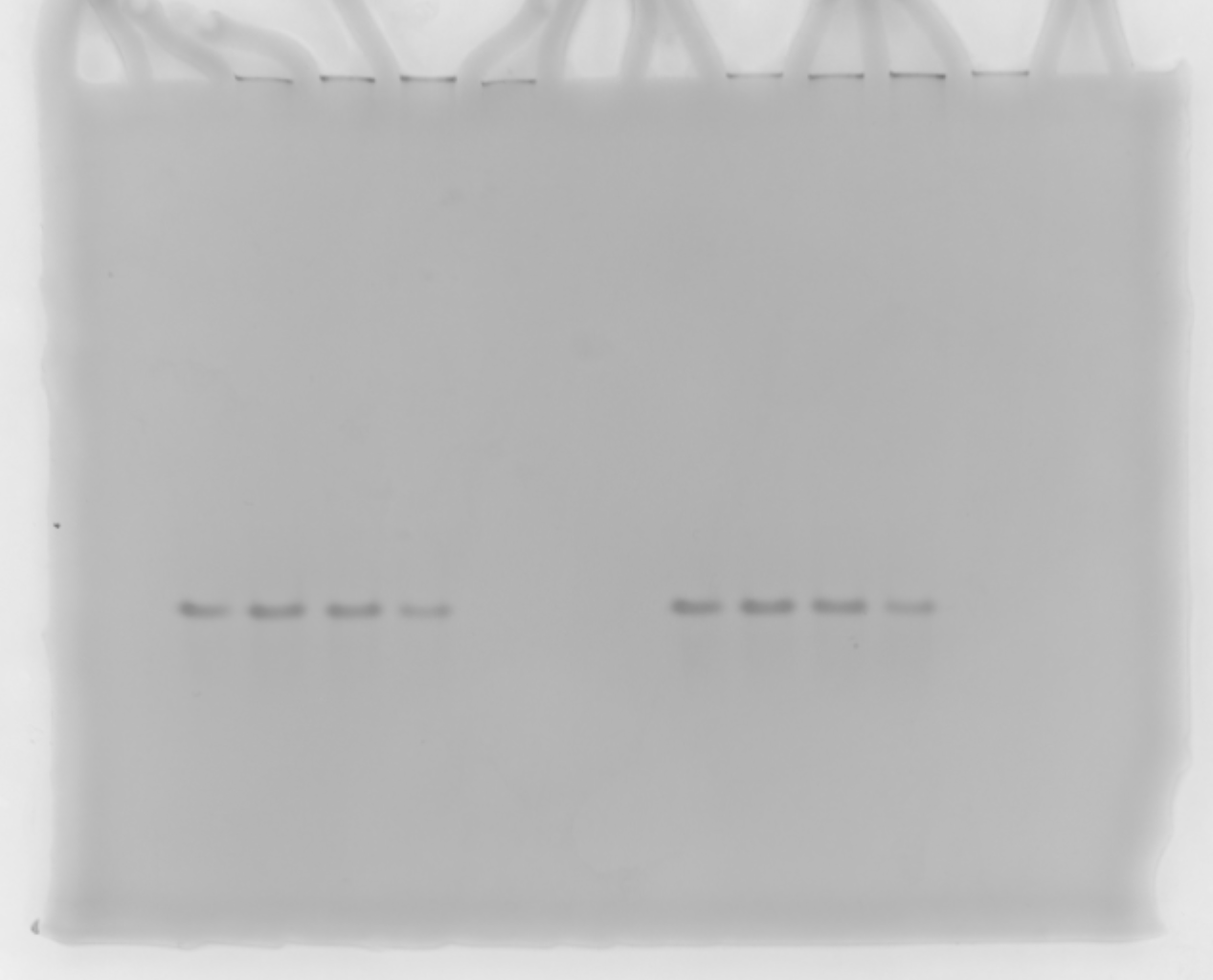

Supplement: Figure 4—figure supplement 2—source data 2. [file elife-101717-fig4-figsupp2-data2.zip › A/sld3Cdc45 1-2,5-3.tif]

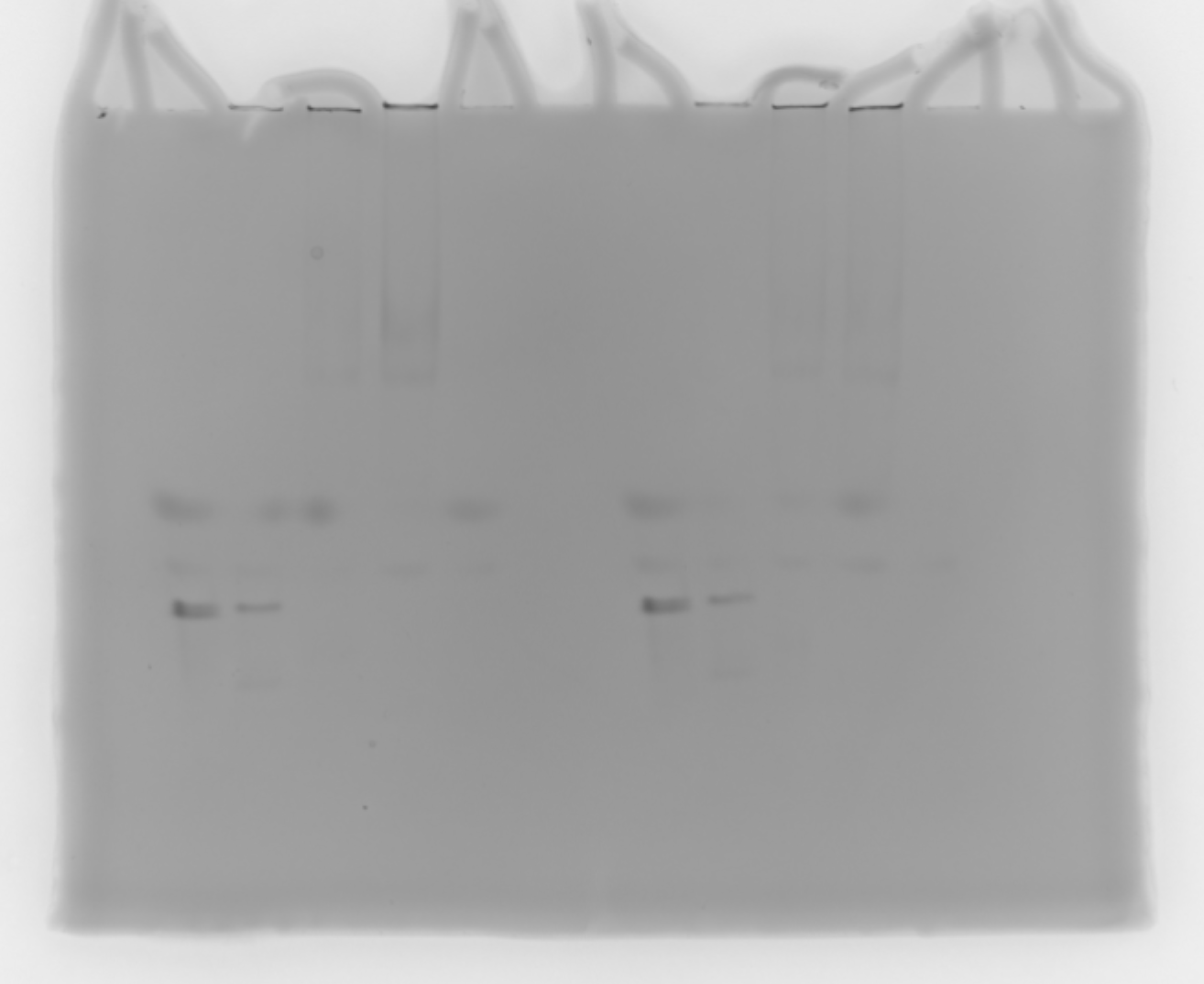

Supplement: Figure 4—figure supplement 2—source data 2. [file elife-101717-fig4-figsupp2-data2.zip › A/Sld3Sld7Cdc45 1-2-3, 1-5-3.tif]

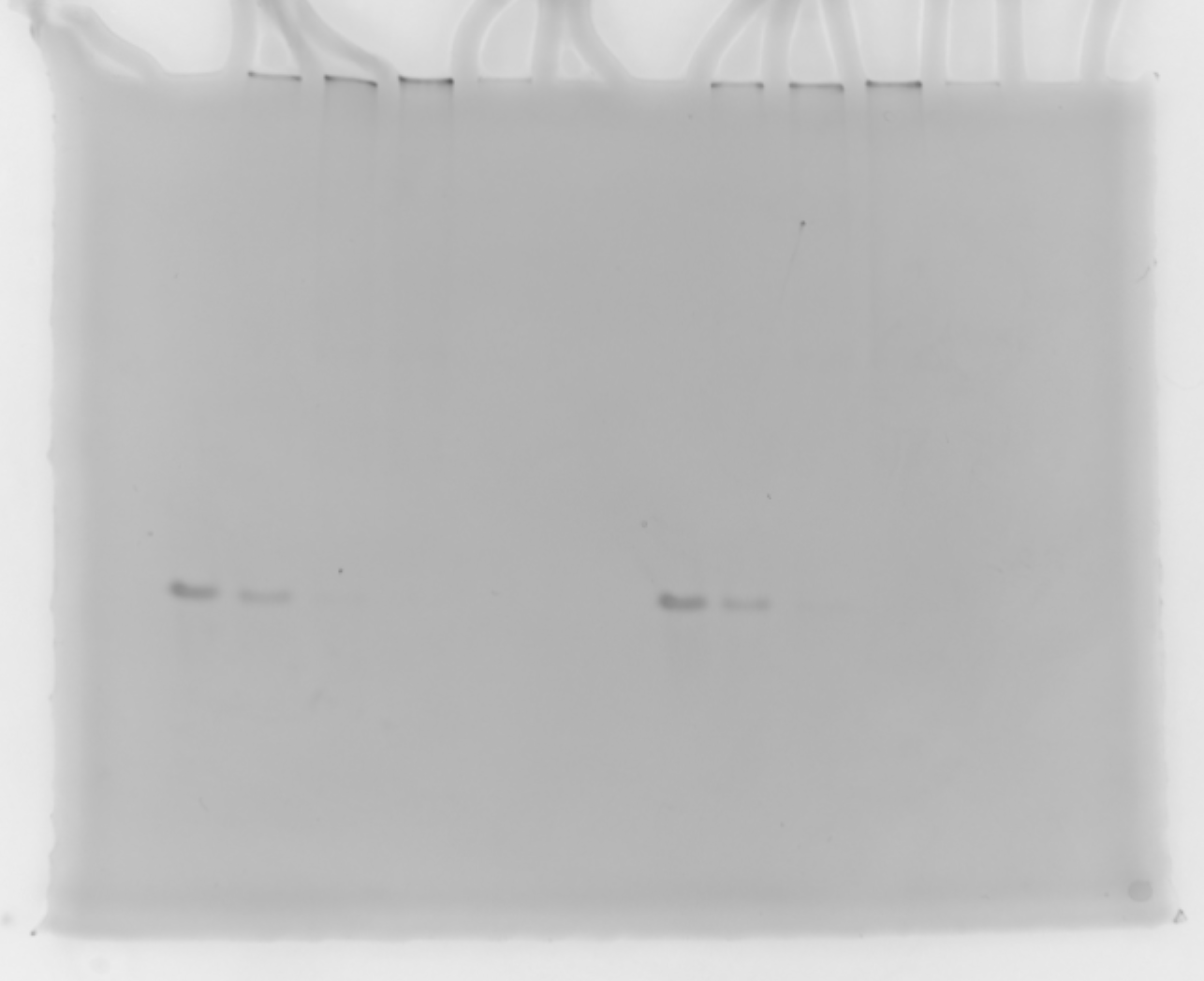

Supplement: Figure 4—figure supplement 2—source data 2. [file elife-101717-fig4-figsupp2-data2.zip › A/sld3 1-2,5-3.tif]

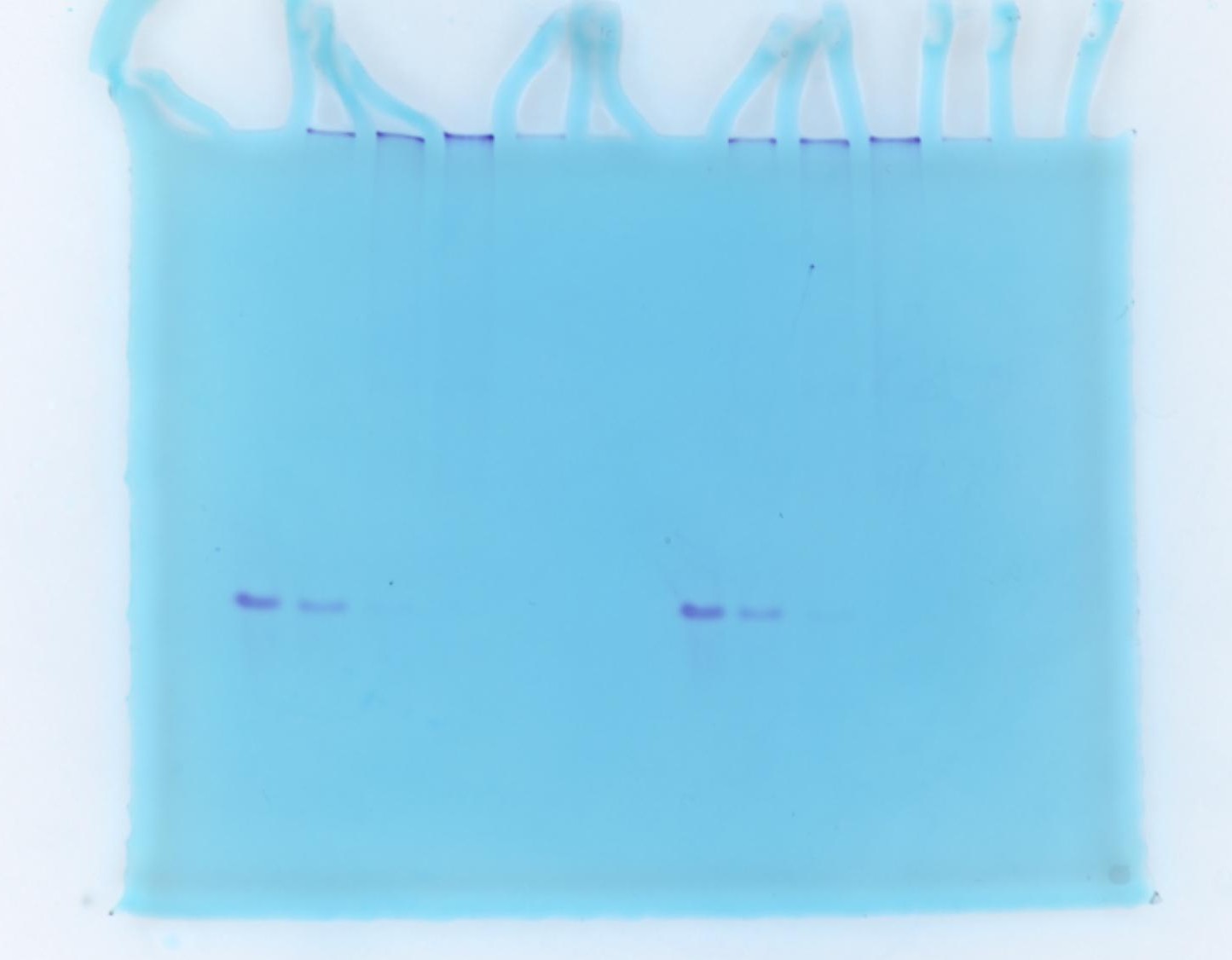

Supplement: Figure 4—figure supplement 2—source data 2. [file elife-101717-fig4-figsupp2-data2.zip › A/sld3 1-2,5-3.jpg]

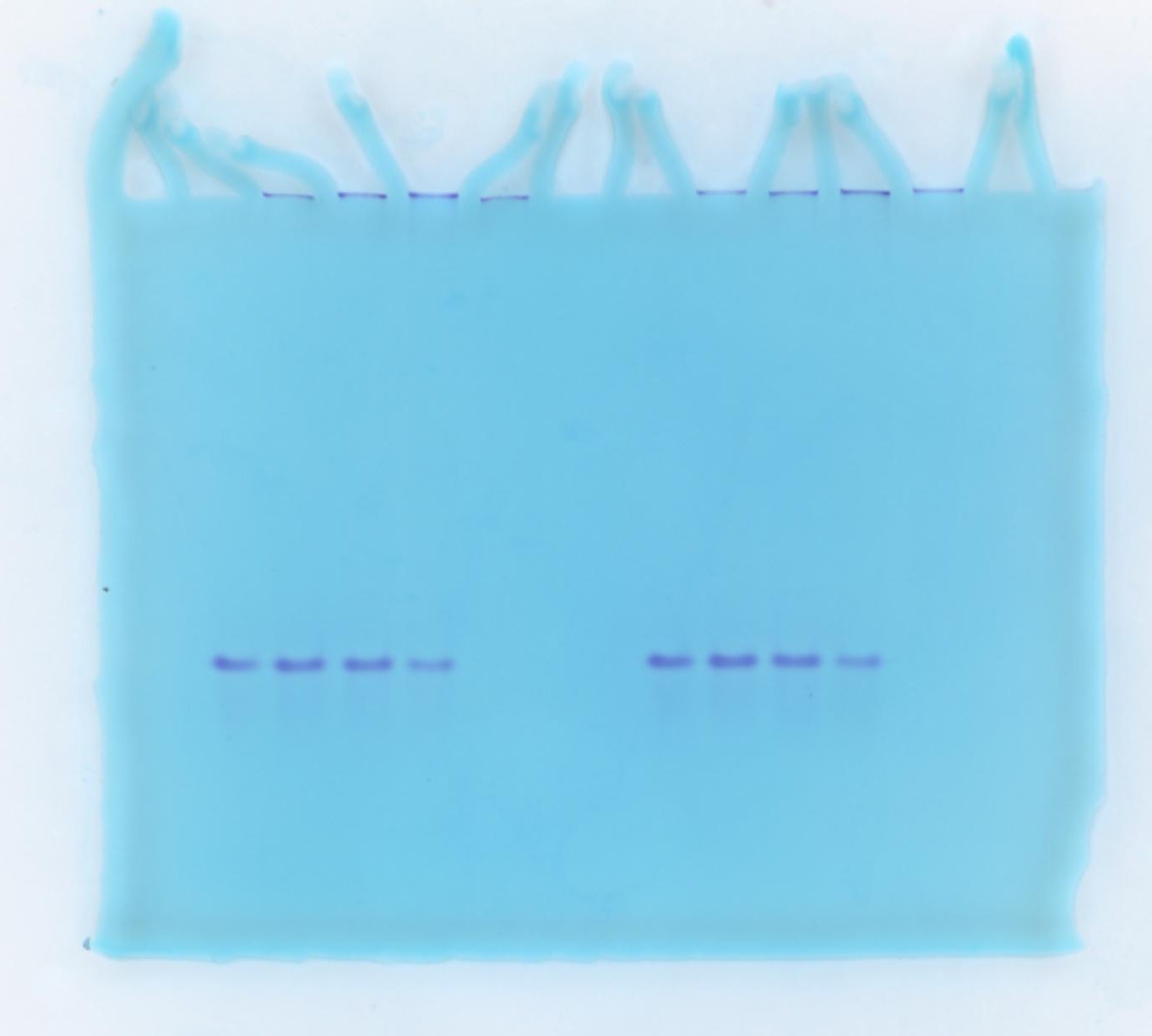

Supplement: Figure 4—figure supplement 2—source data 2. [file elife-101717-fig4-figsupp2-data2.zip › A/sld3cdc45 1-2,5-3.jpg]

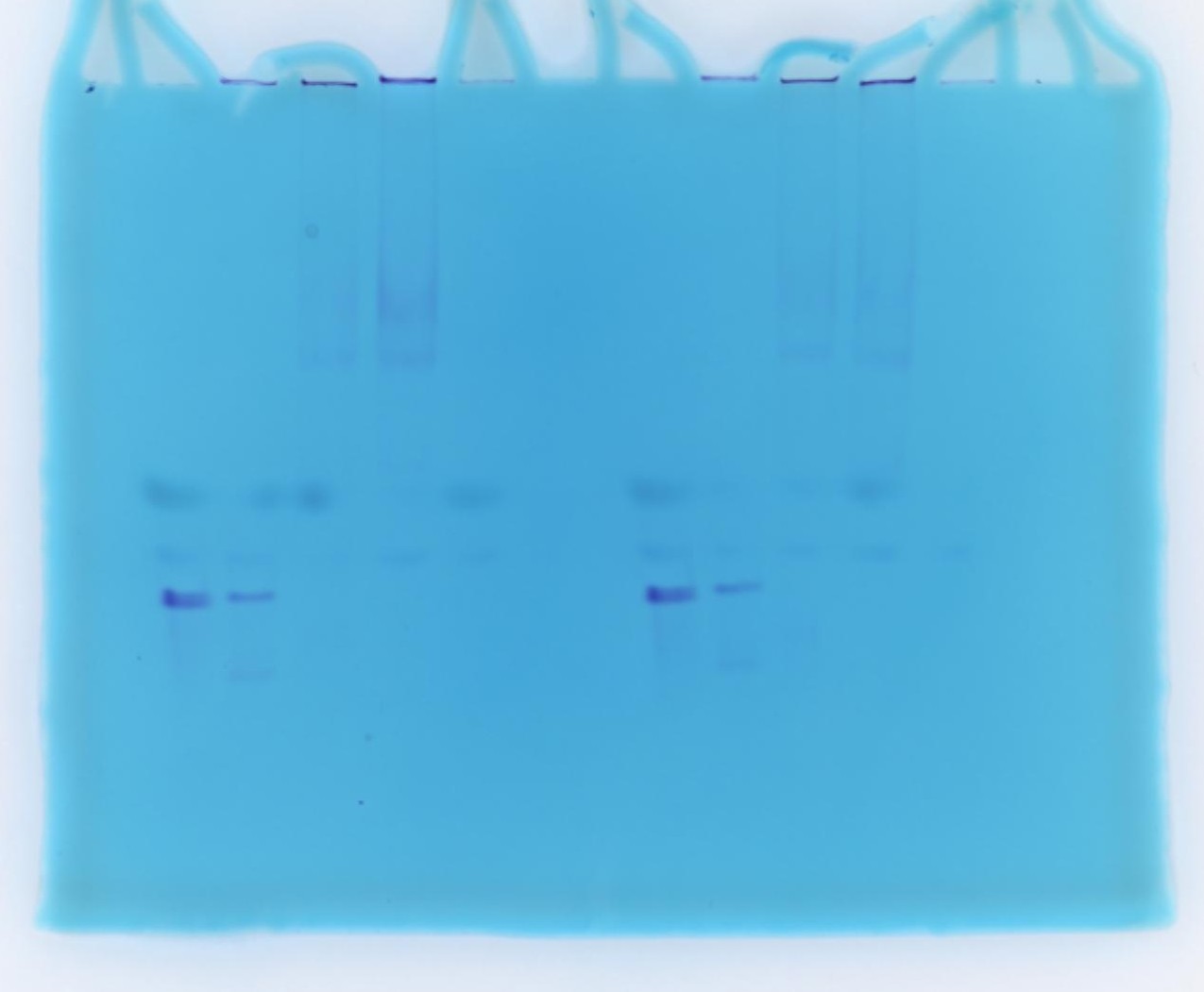

Supplement: Figure 4—figure supplement 2—source data 2. [file elife-101717-fig4-figsupp2-data2.zip › A/Sld3Sld7Cdc45 1-2-3, 1-5-3.jpg]

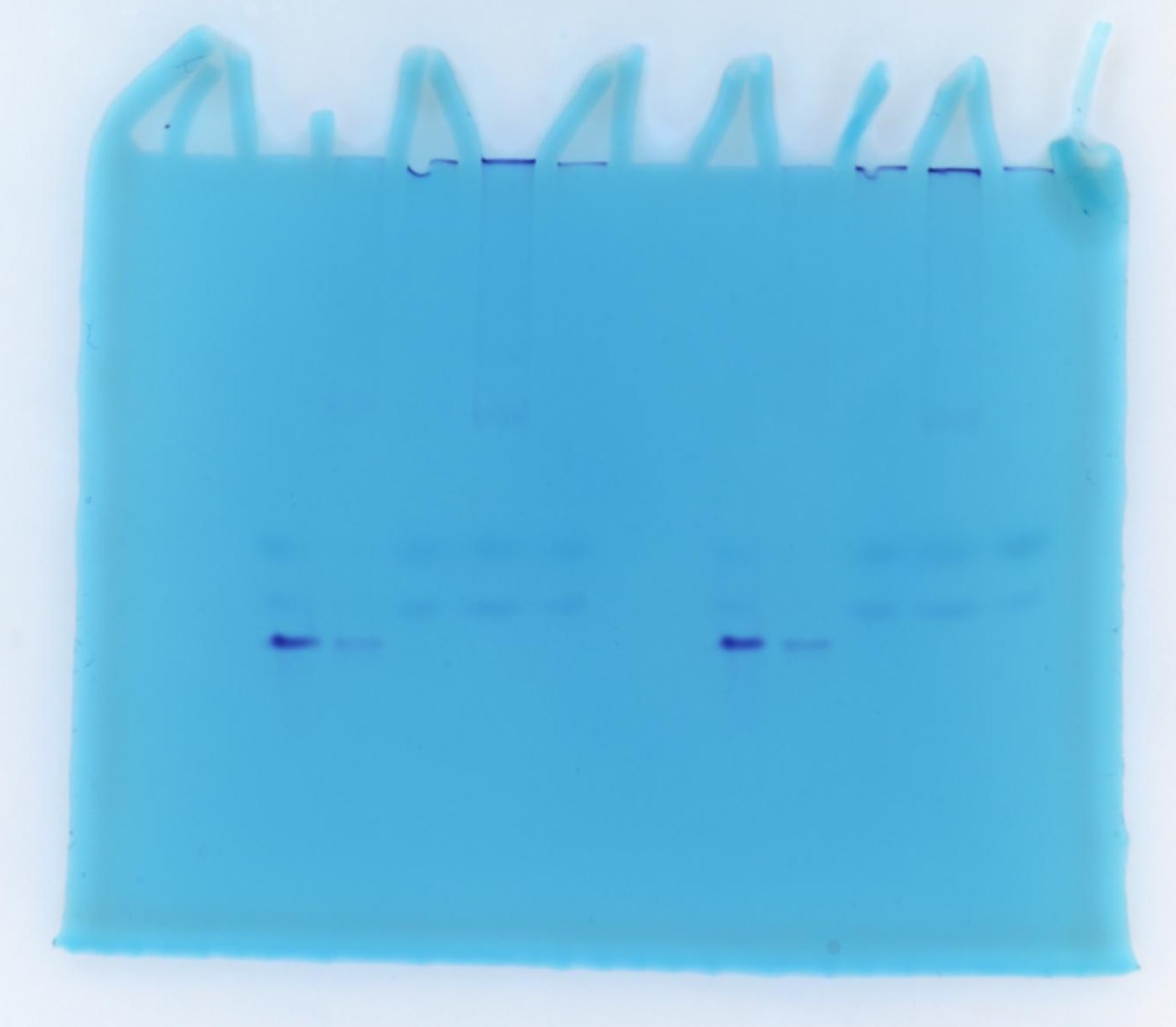

Supplement: Figure 4—figure supplement 2—source data 2. [file elife-101717-fig4-figsupp2-data2.zip › A/sld3sld7 1-2-3,1-5-3.jpg]

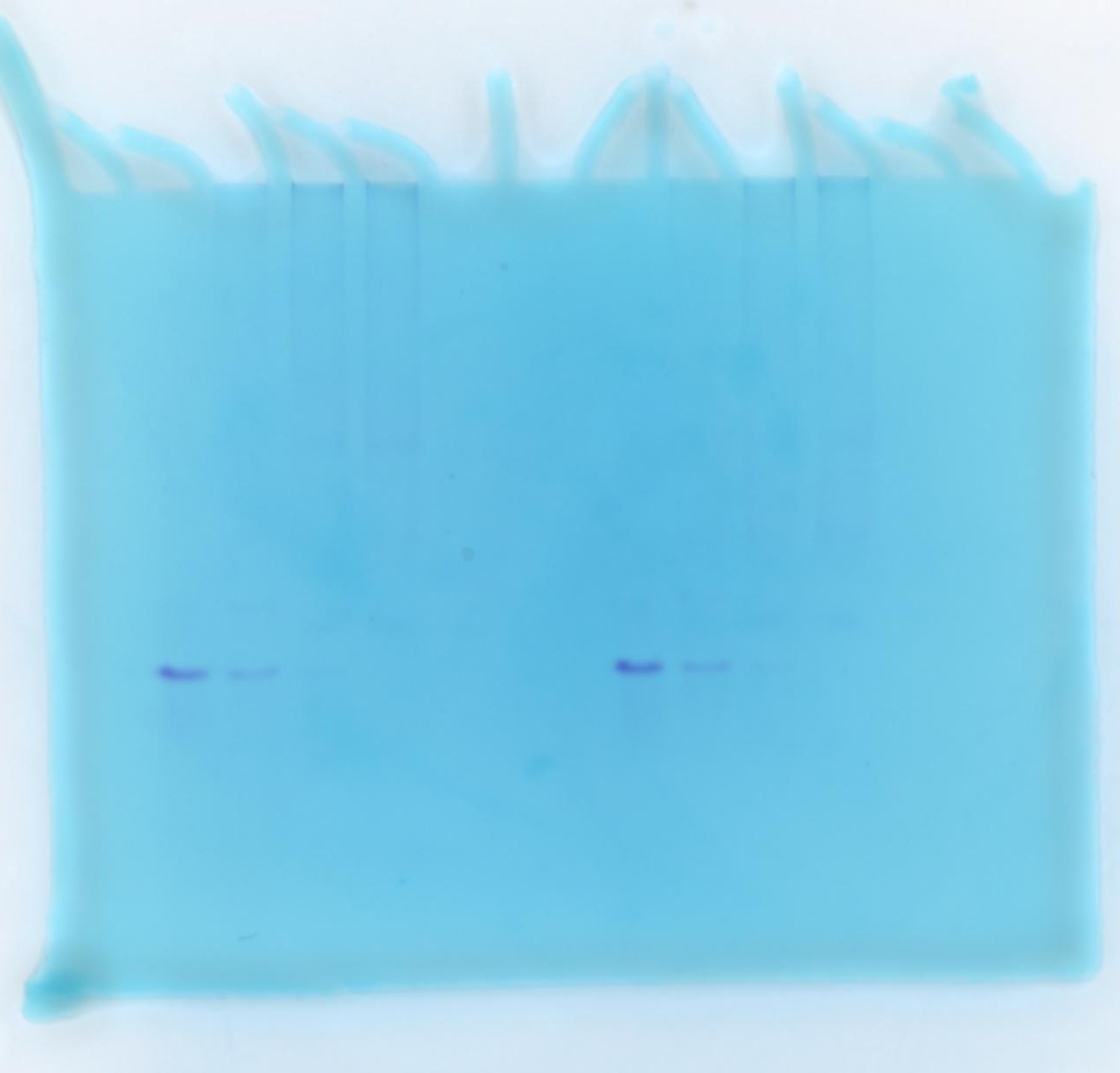

Supplement: Figure 4—figure supplement 2—source data 2. [file elife-101717-fig4-figsupp2-data2.zip › A/sld3sld7 1-2-1,2.jpg]

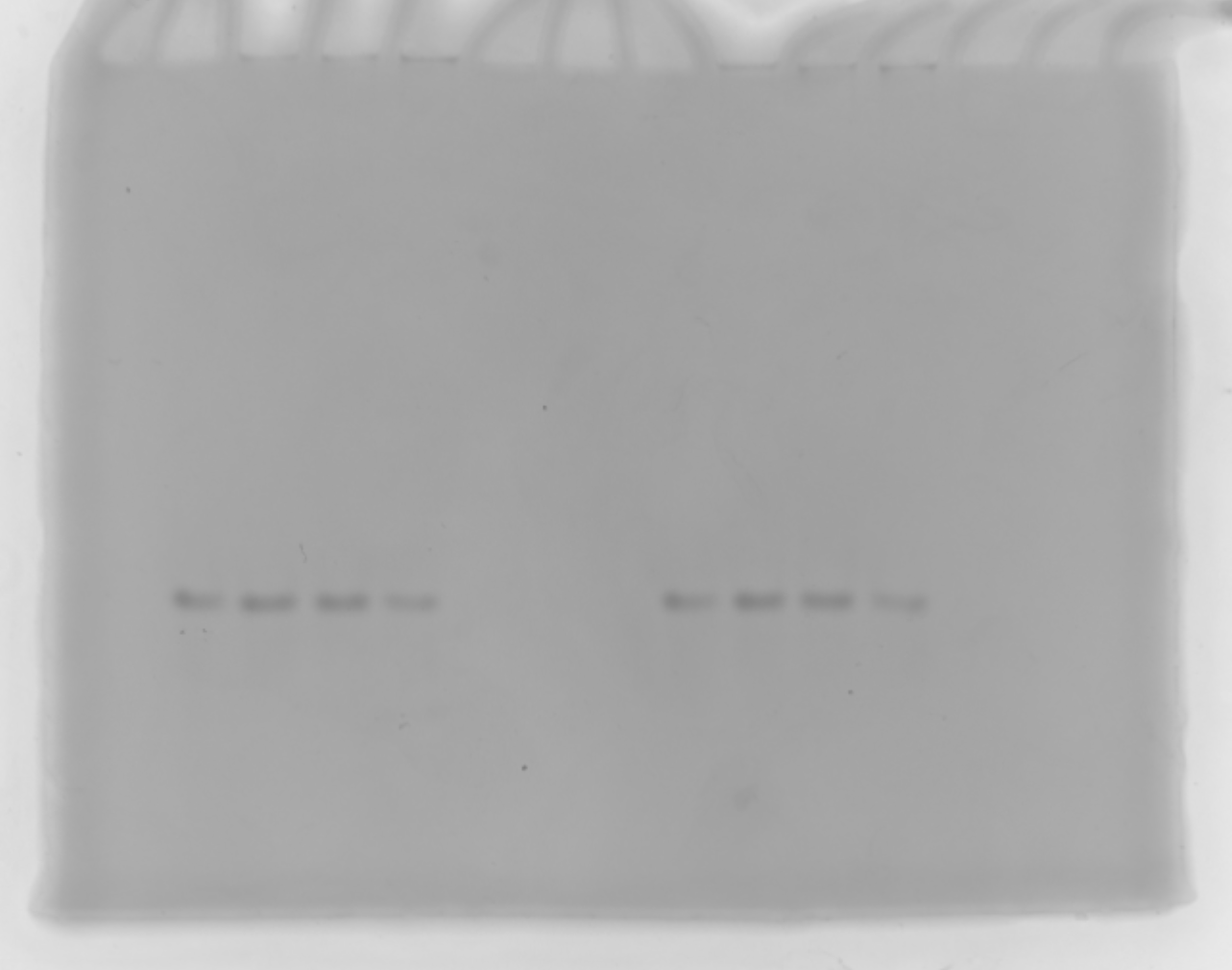

Supplement: Figure 4—figure supplement 2—source data 2. [file elife-101717-fig4-figsupp2-data2.zip › A/sld3CBDcdc45 1-2-1,2.tif]

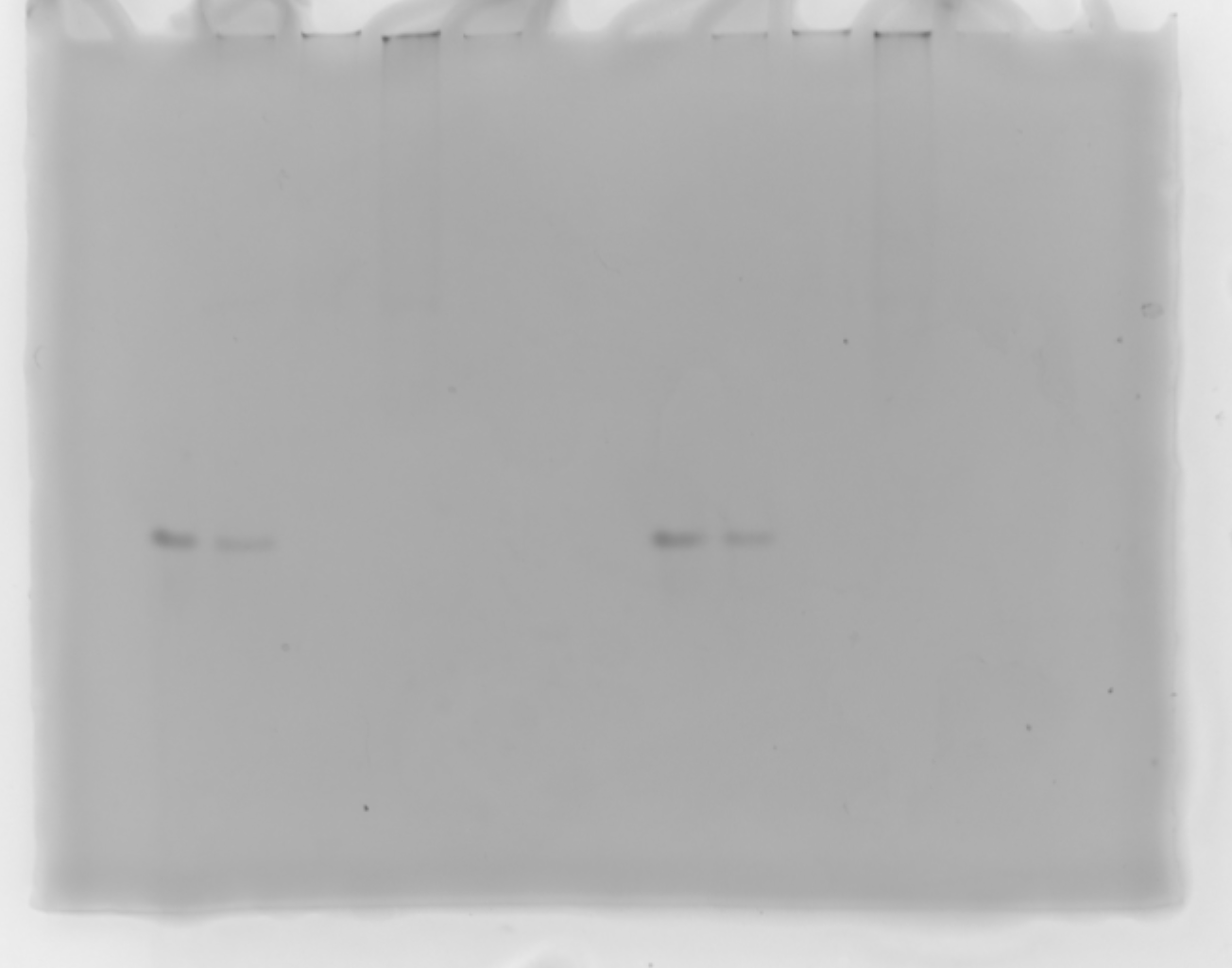

Supplement: Figure 4—figure supplement 2—source data 2. [file elife-101717-fig4-figsupp2-data2.zip › A/sld3 1-2-1,2.tif]

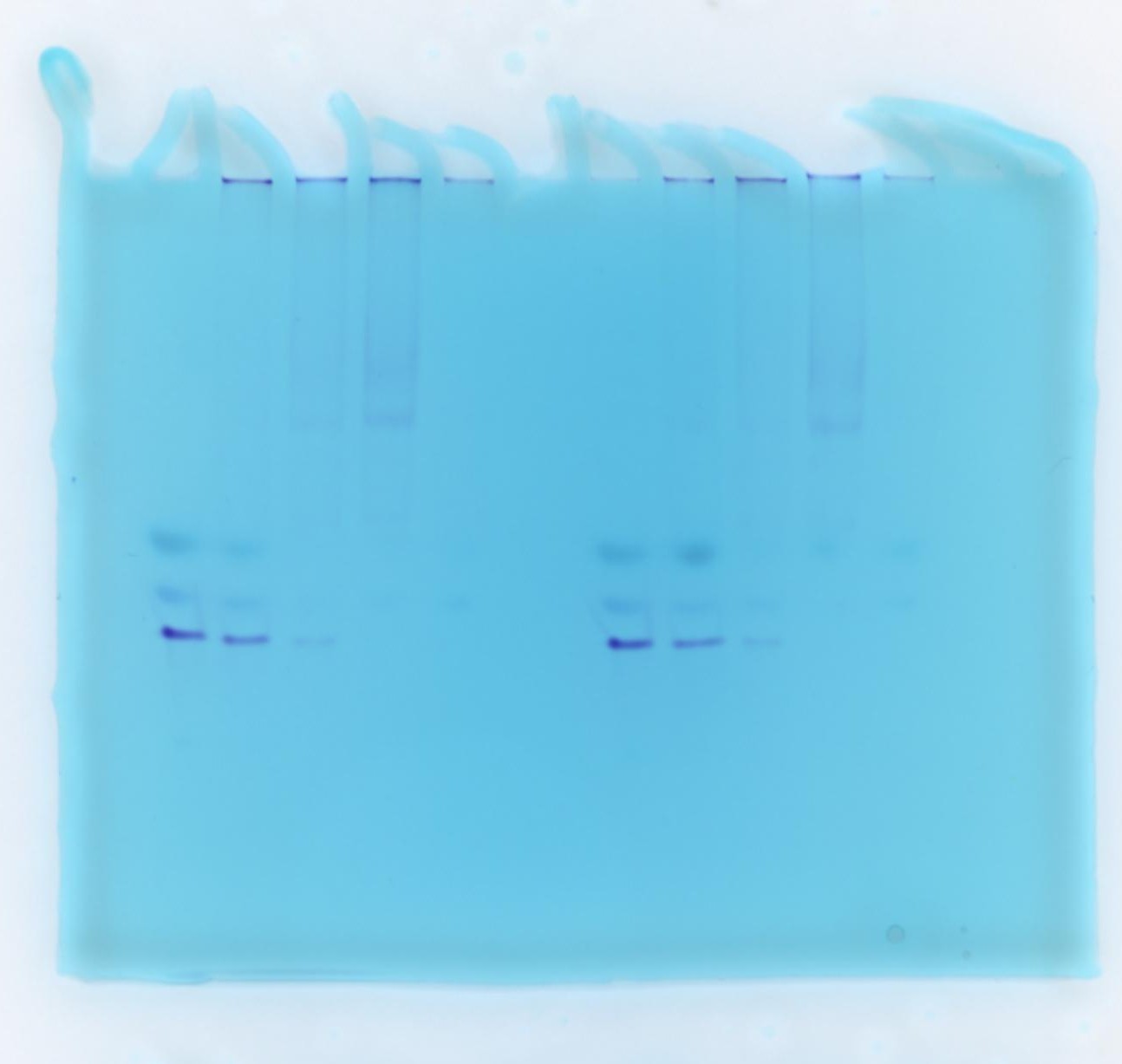

Supplement: Figure 4—figure supplement 2—source data 2. [file elife-101717-fig4-figsupp2-data2.zip › A/Sld3Sld7Cdc45 1-2-1,2.jpg]

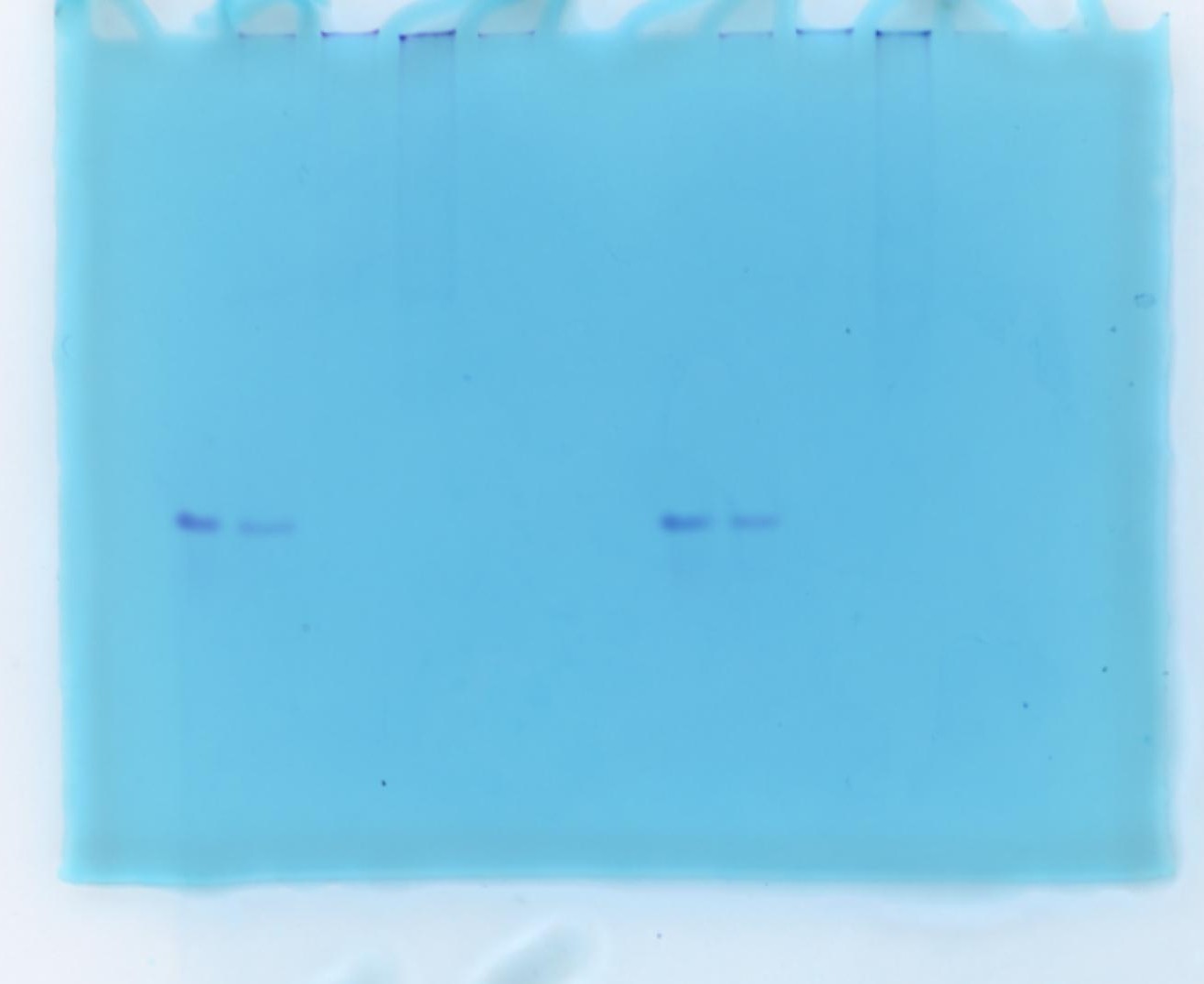

Supplement: Figure 4—figure supplement 2—source data 2. [file elife-101717-fig4-figsupp2-data2.zip › A/sld3 1-2-1,2.jpg]

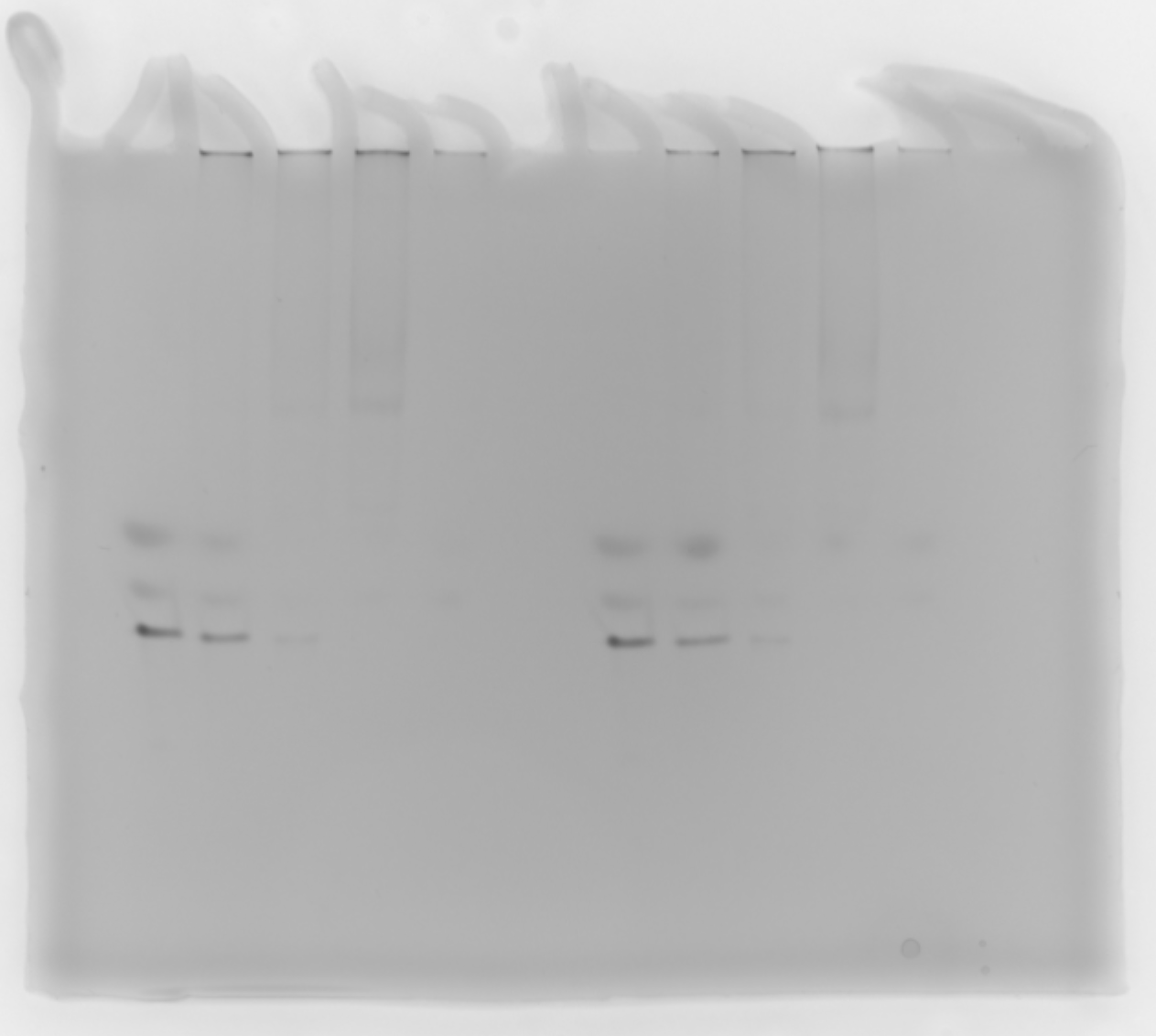

Supplement: Figure 4—figure supplement 2—source data 2. [file elife-101717-fig4-figsupp2-data2.zip › A/Sld3Sld7Cdc45 1-2-1,2.tif]

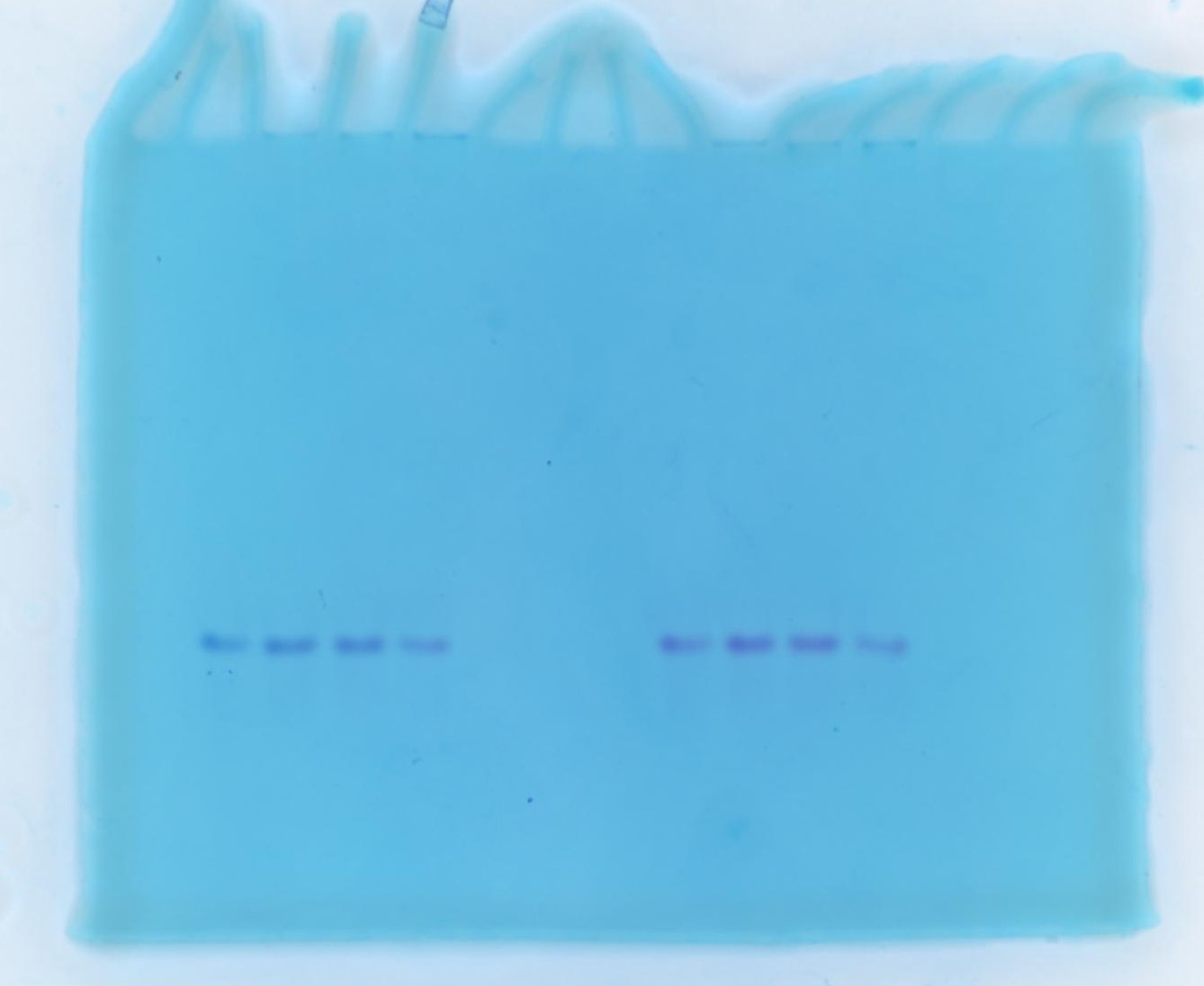

Supplement: Figure 4—figure supplement 2—source data 2. [file elife-101717-fig4-figsupp2-data2.zip › A/sld3CBDcdc45 1-2-1,2.jpg]

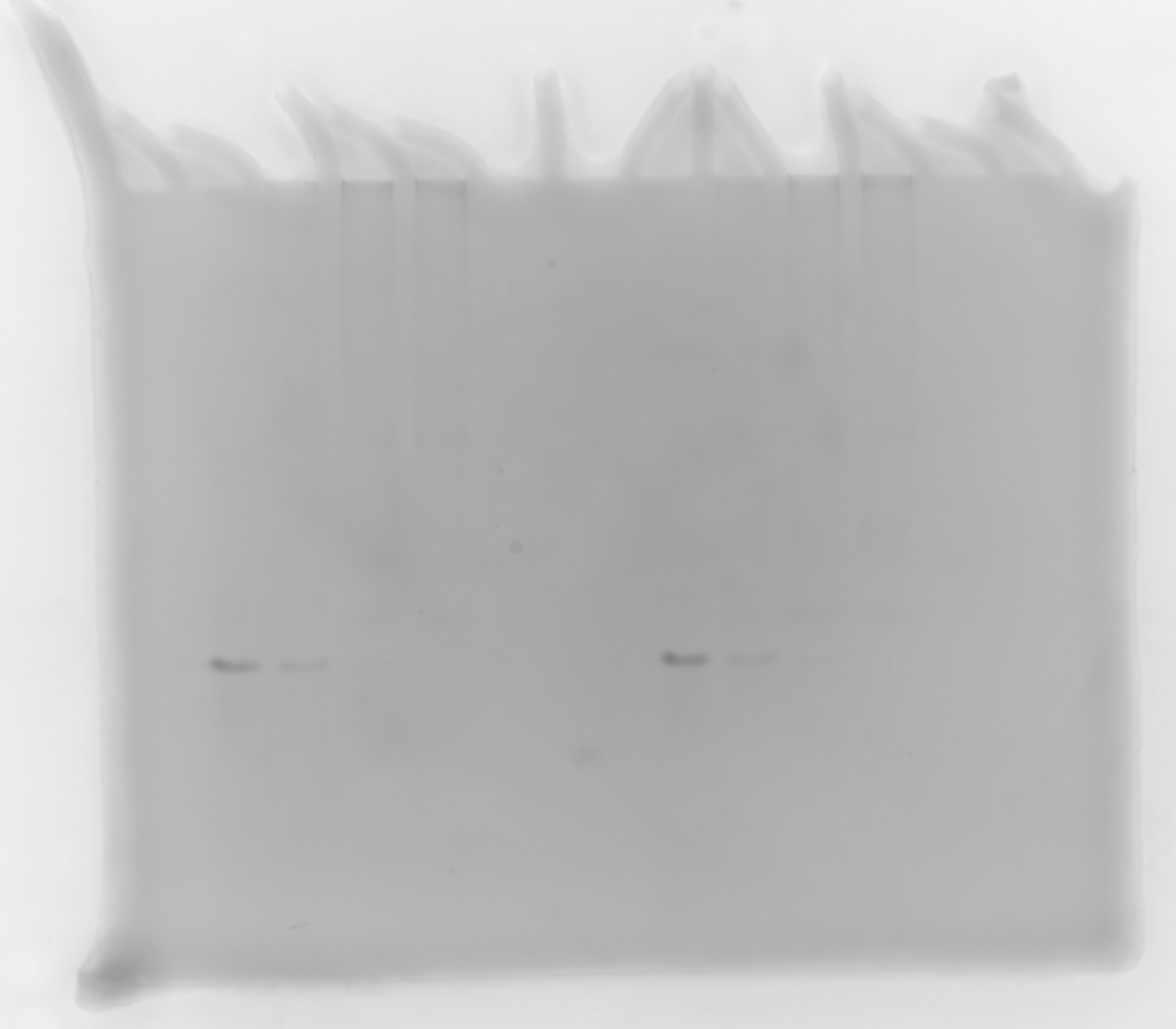

Supplement: Figure 4—figure supplement 2—source data 2. [file elife-101717-fig4-figsupp2-data2.zip › A/sld3sld7 1-2-1,2.tif]

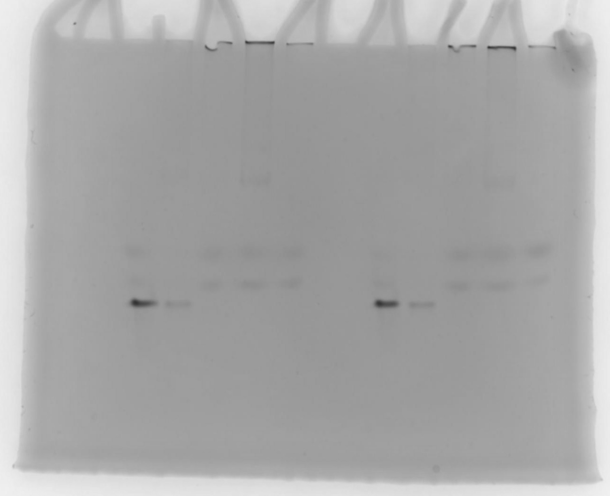

Supplement: Figure 4—figure supplement 2—source data 2. [file elife-101717-fig4-figsupp2-data2.zip › A/sld3sld7 1-2-3,1-5-3.tif]

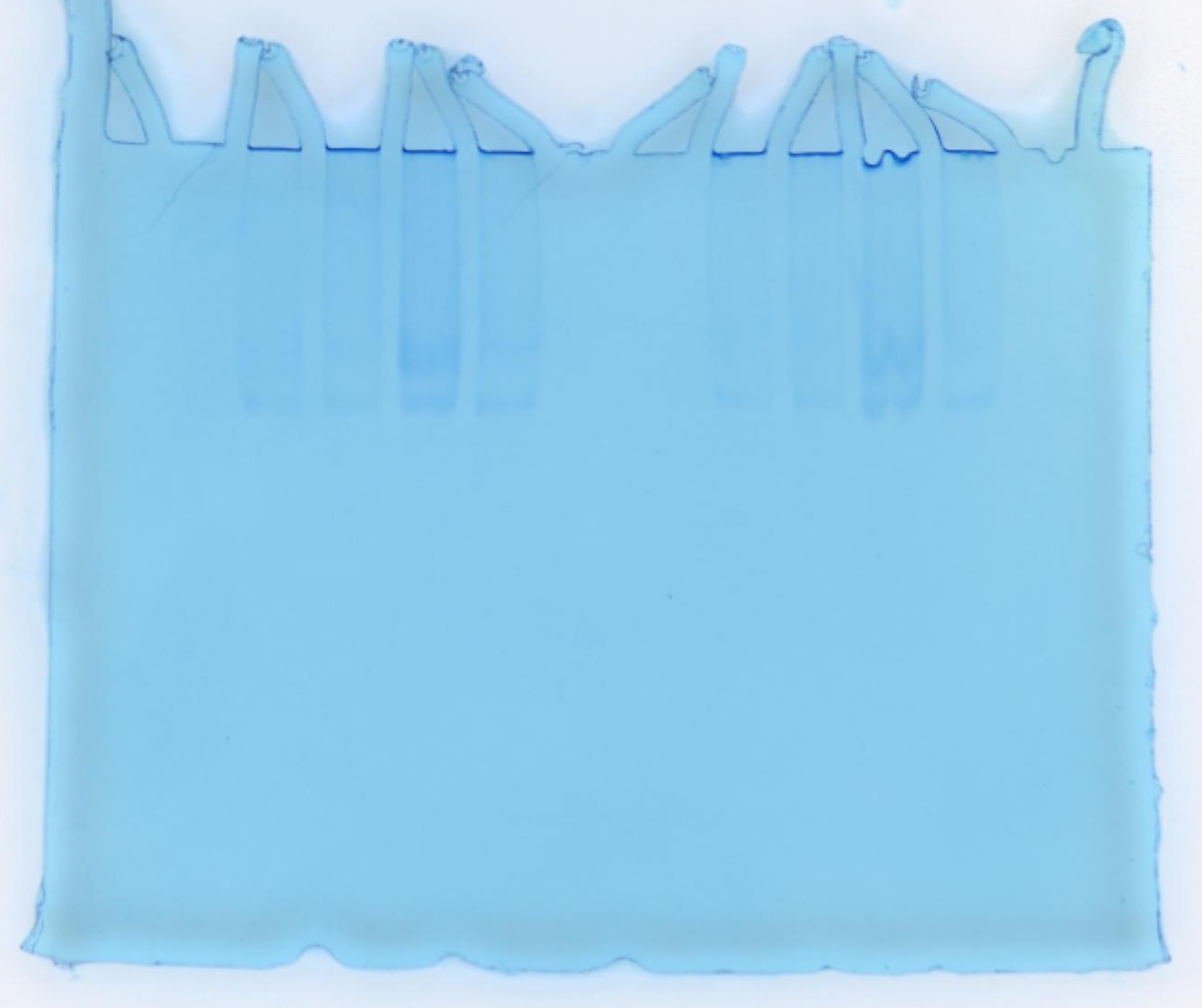

Supplement: Figure 4—figure supplement 2—source data 2. [file elife-101717-fig4-figsupp2-data2.zip › B/sld3sld7 1-5-1,2.jpg]

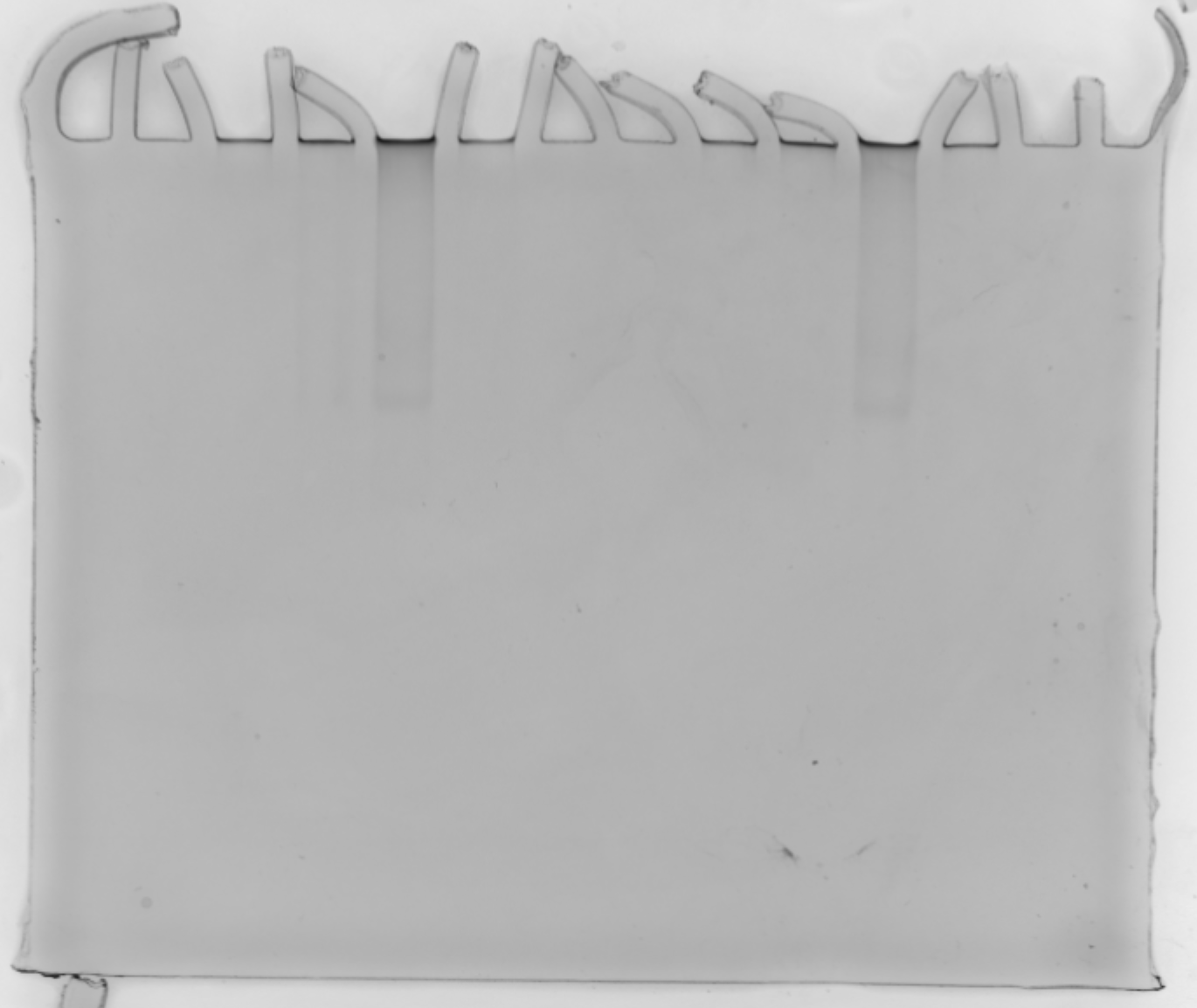

Supplement: Figure 4—figure supplement 2—source data 2. [file elife-101717-fig4-figsupp2-data2.zip › B/sld3cdc45 1-2,5-3.tif]

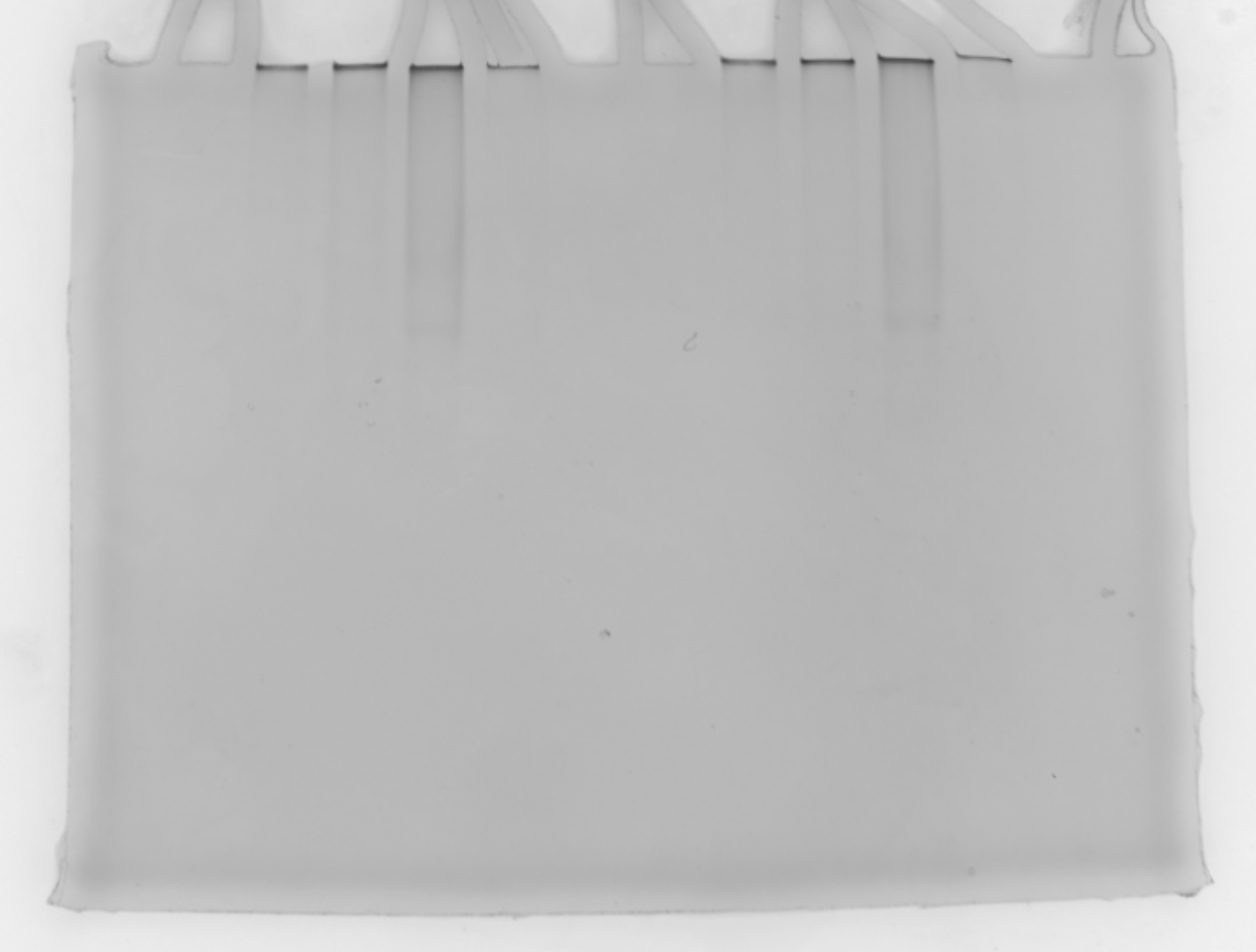

Supplement: Figure 4—figure supplement 2—source data 2. [file elife-101717-fig4-figsupp2-data2.zip › B/sld3cdc45 1-5-1,2.tif]

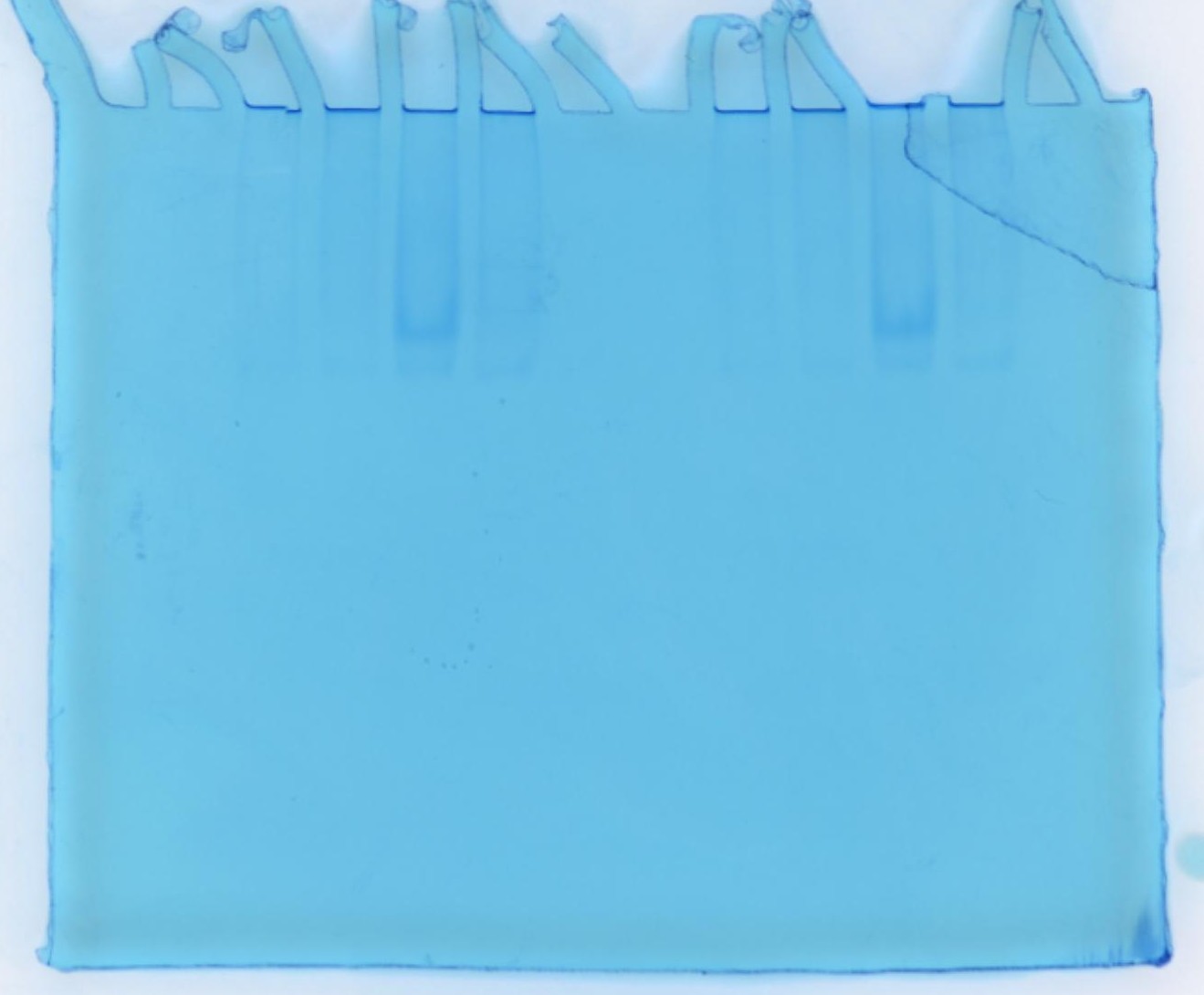

Supplement: Figure 4—figure supplement 2—source data 2. [file elife-101717-fig4-figsupp2-data2.zip › B/sld3sld7 1-2,5-3.jpg]

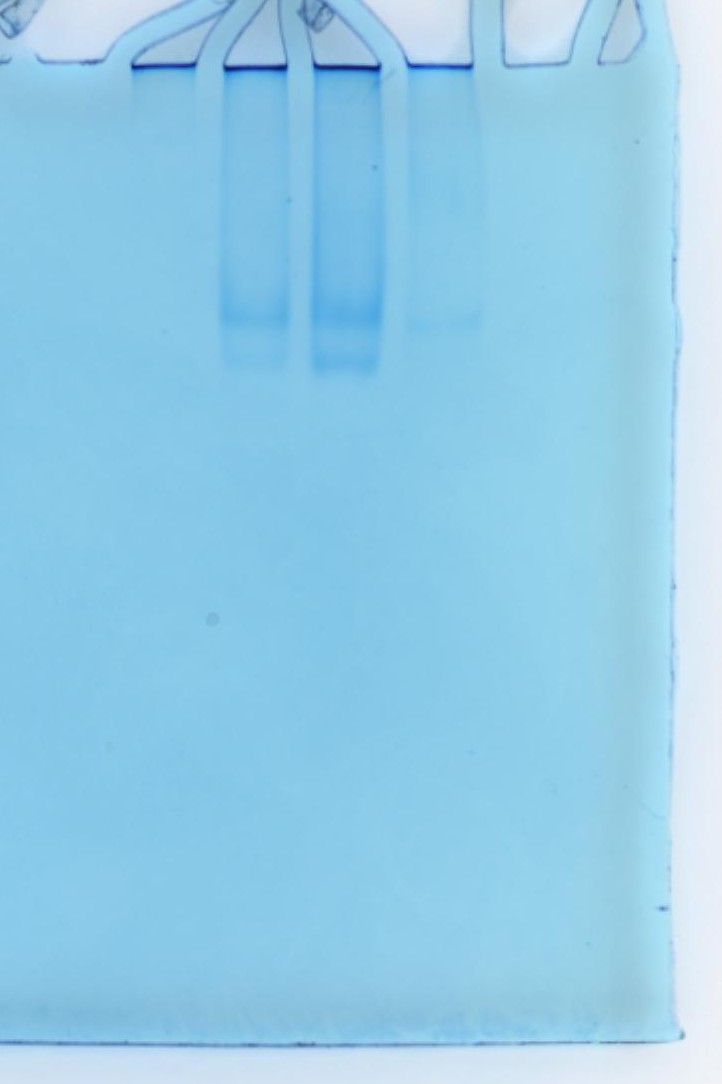

Supplement: Figure 4—figure supplement 2—source data 2. [file elife-101717-fig4-figsupp2-data2.zip › B/Sld7Sld3Cdc45 1-2-3.jpg]

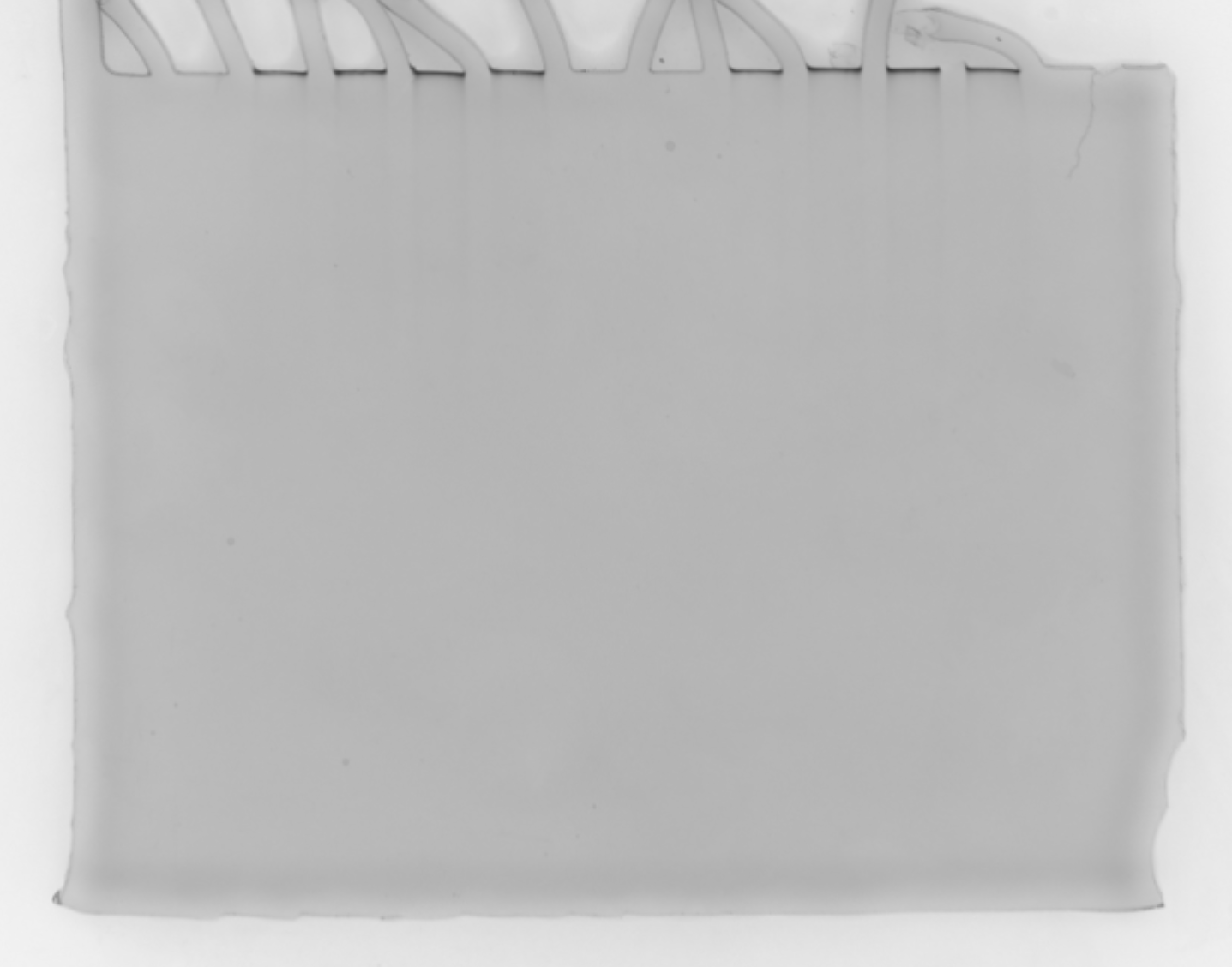

Supplement: Figure 4—figure supplement 2—source data 2. [file elife-101717-fig4-figsupp2-data2.zip › B/sld3 1-2,5-3.tif]

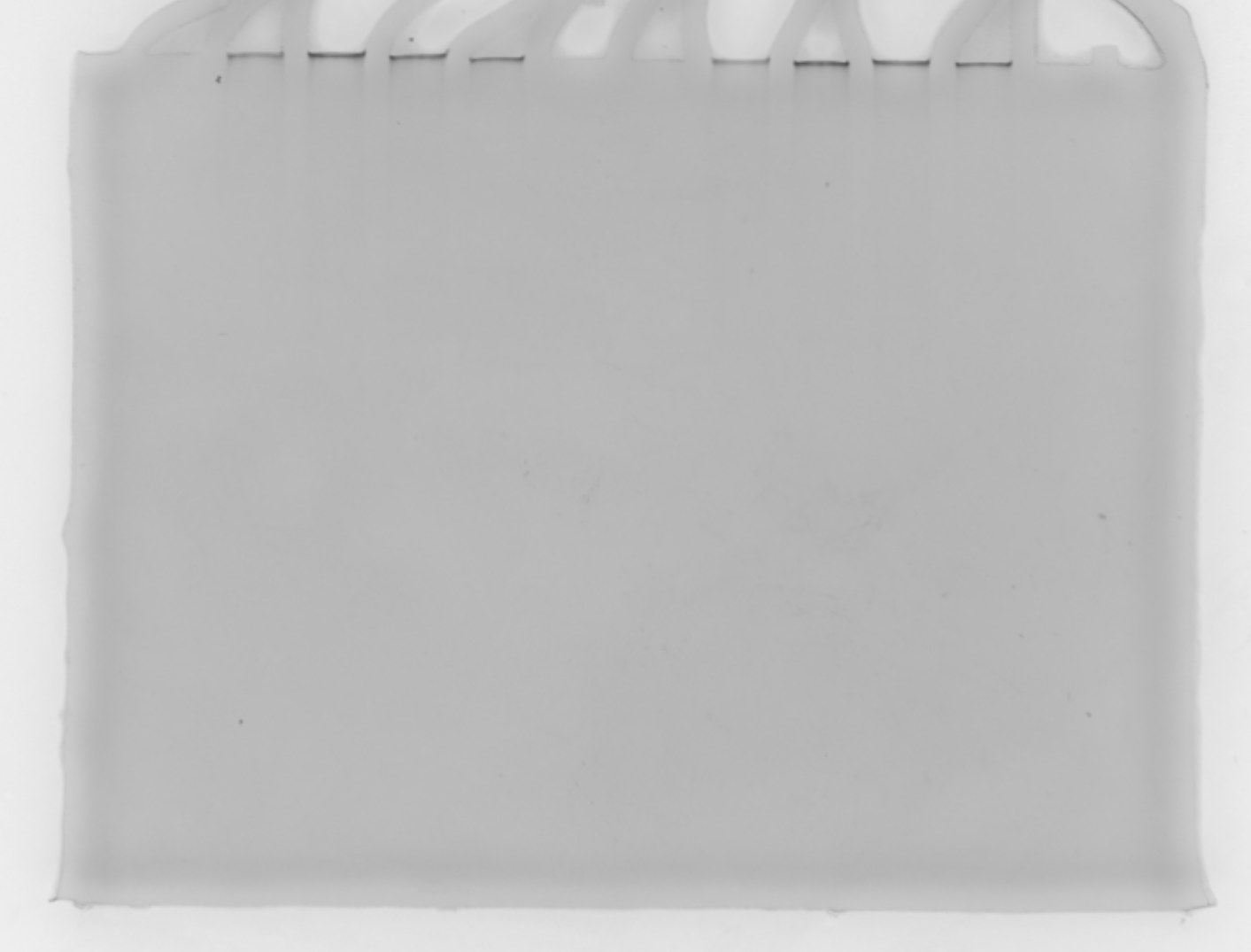

Supplement: Figure 4—figure supplement 2—source data 2. [file elife-101717-fig4-figsupp2-data2.zip › B/sld3 1-5-1,2.tif]

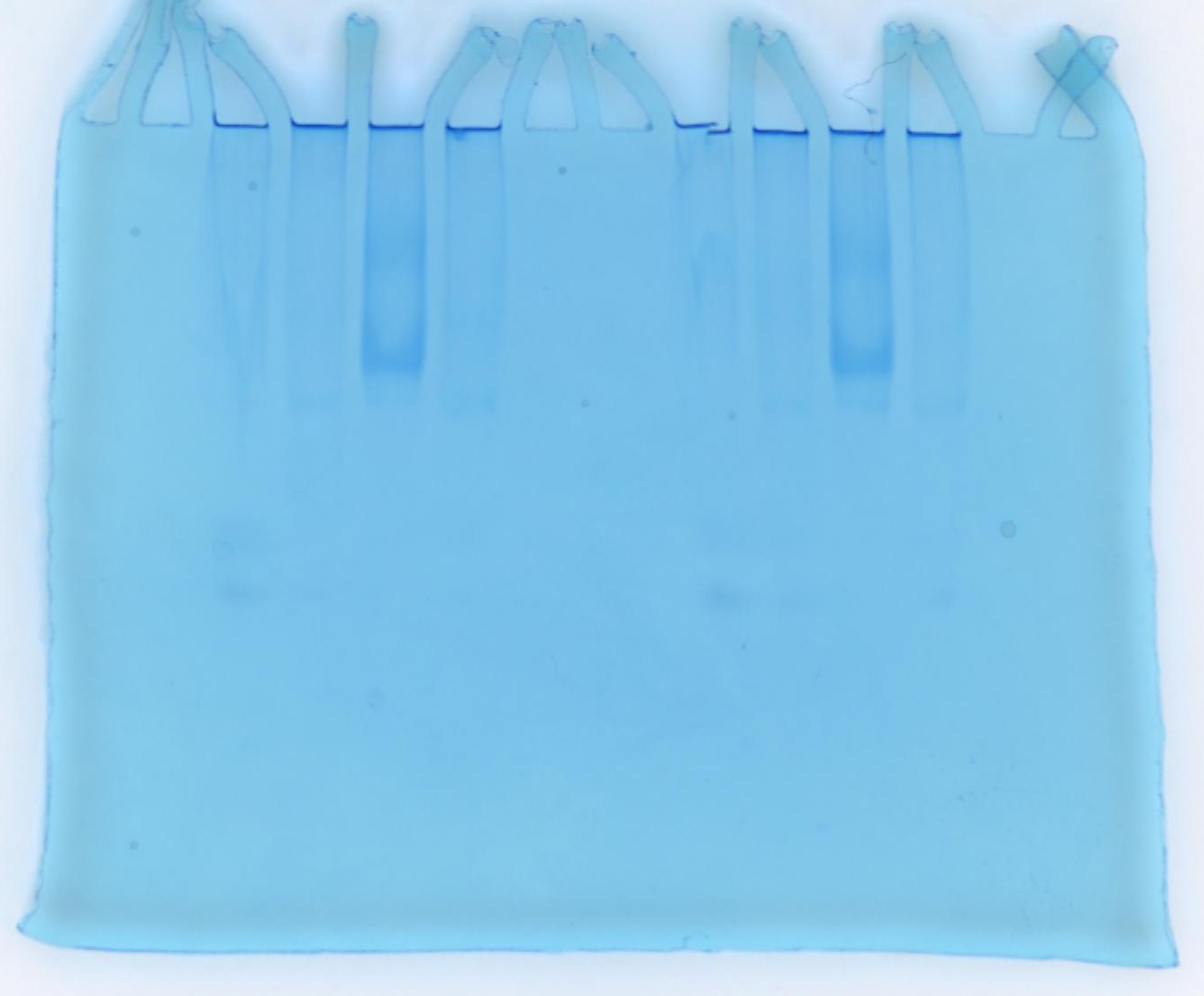

Supplement: Figure 4—figure supplement 2—source data 2. [file elife-101717-fig4-figsupp2-data2.zip › B/Sld3Sld7Cdc45 1-5-1,2.jpg]

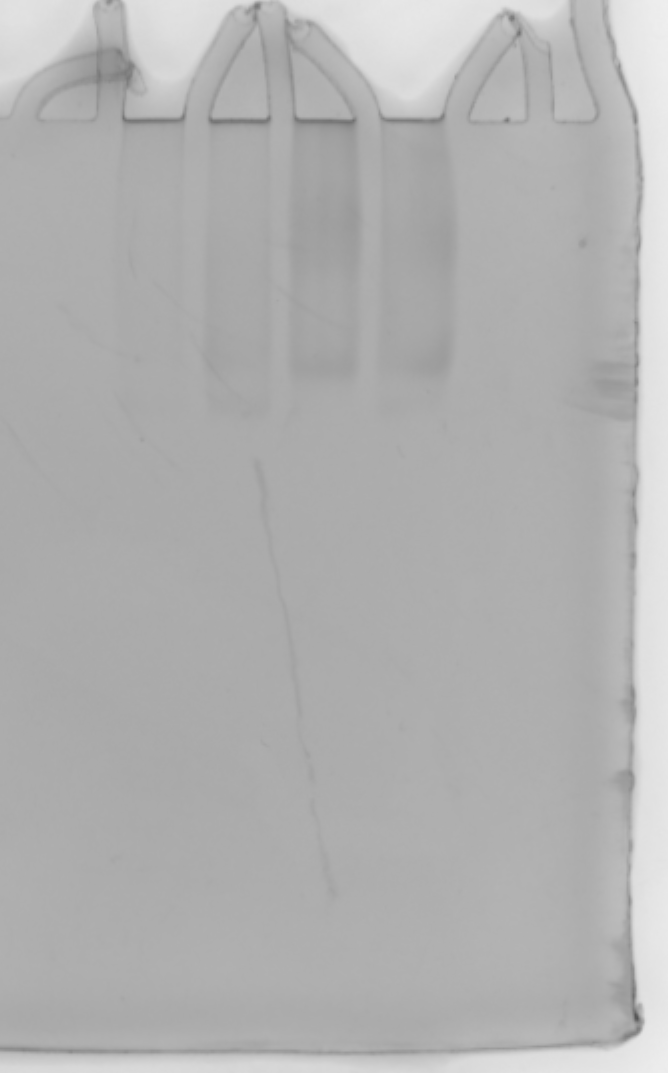

Supplement: Figure 4—figure supplement 2—source data 2. [file elife-101717-fig4-figsupp2-data2.zip › B/Sld7Sld3Cdc45 1-5-3.tif]

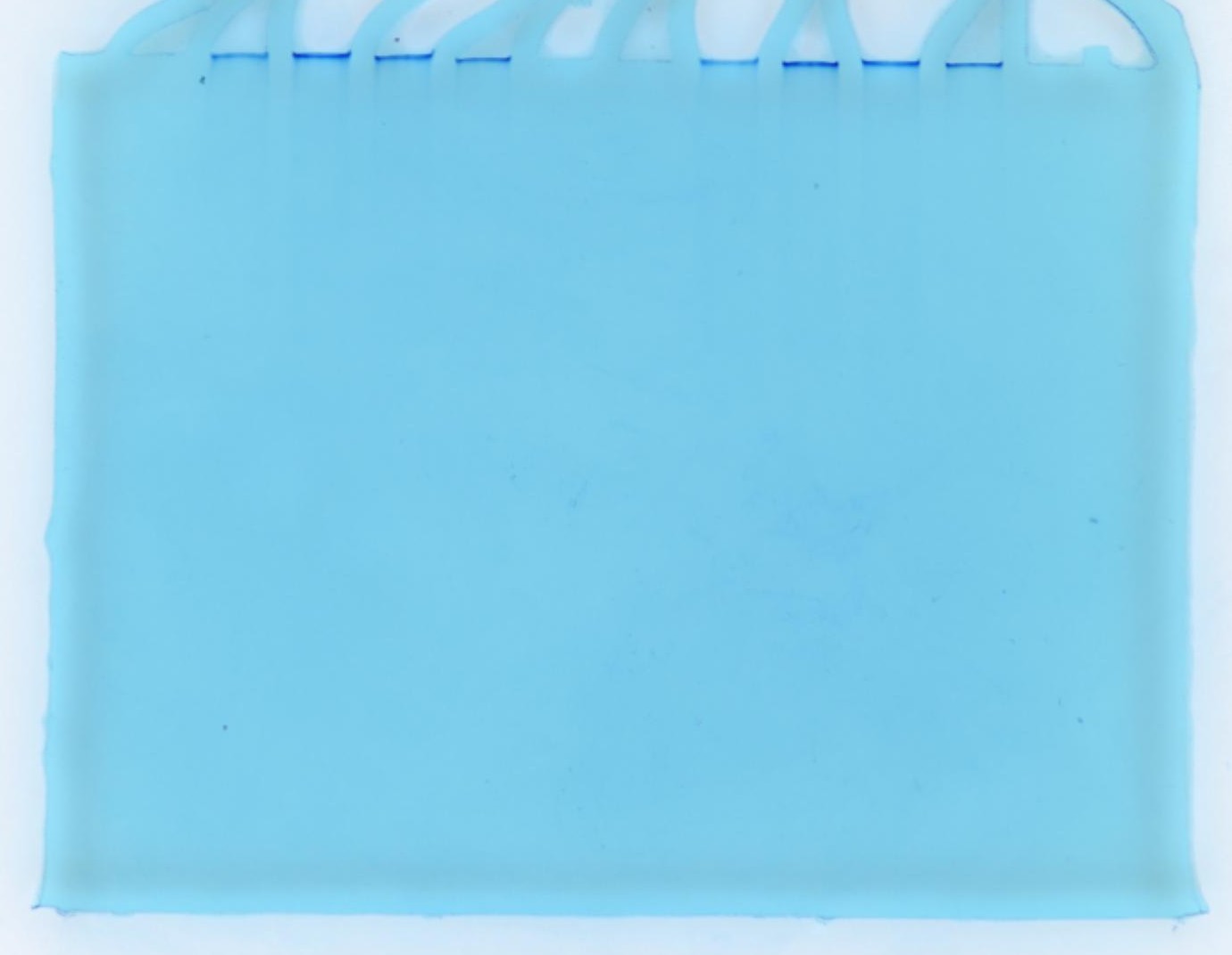

Supplement: Figure 4—figure supplement 2—source data 2. [file elife-101717-fig4-figsupp2-data2.zip › B/sld3 1-5-1,2.jpg]

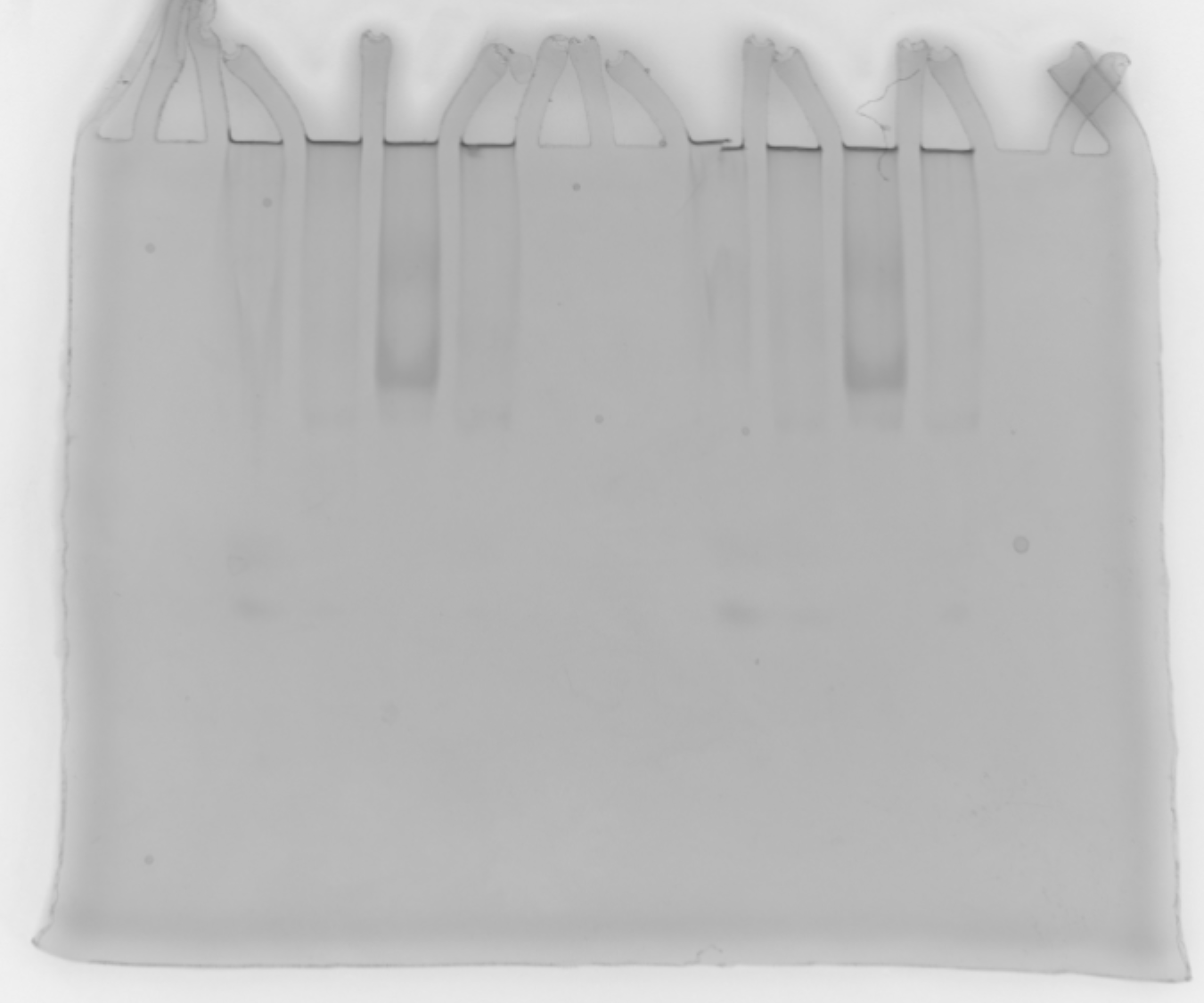

Supplement: Figure 4—figure supplement 2—source data 2. [file elife-101717-fig4-figsupp2-data2.zip › B/Sld3Sld7Cdc45 1-5-1,2.tif]

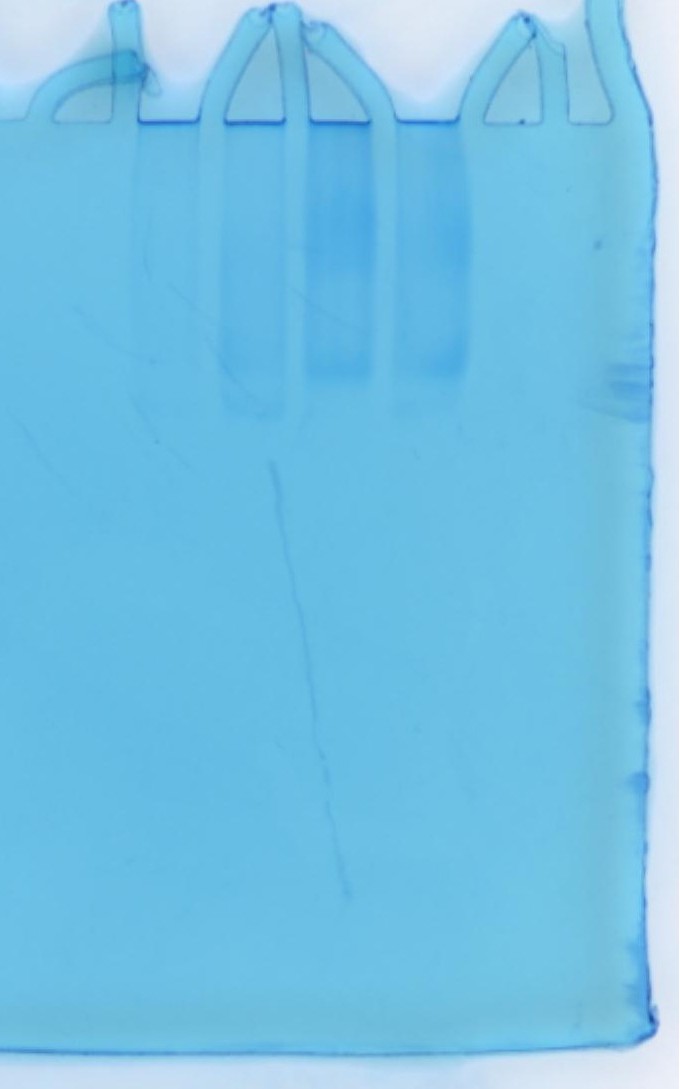

Supplement: Figure 4—figure supplement 2—source data 2. [file elife-101717-fig4-figsupp2-data2.zip › B/Sld7Sld3Cdc45 1-5-3.jpg]

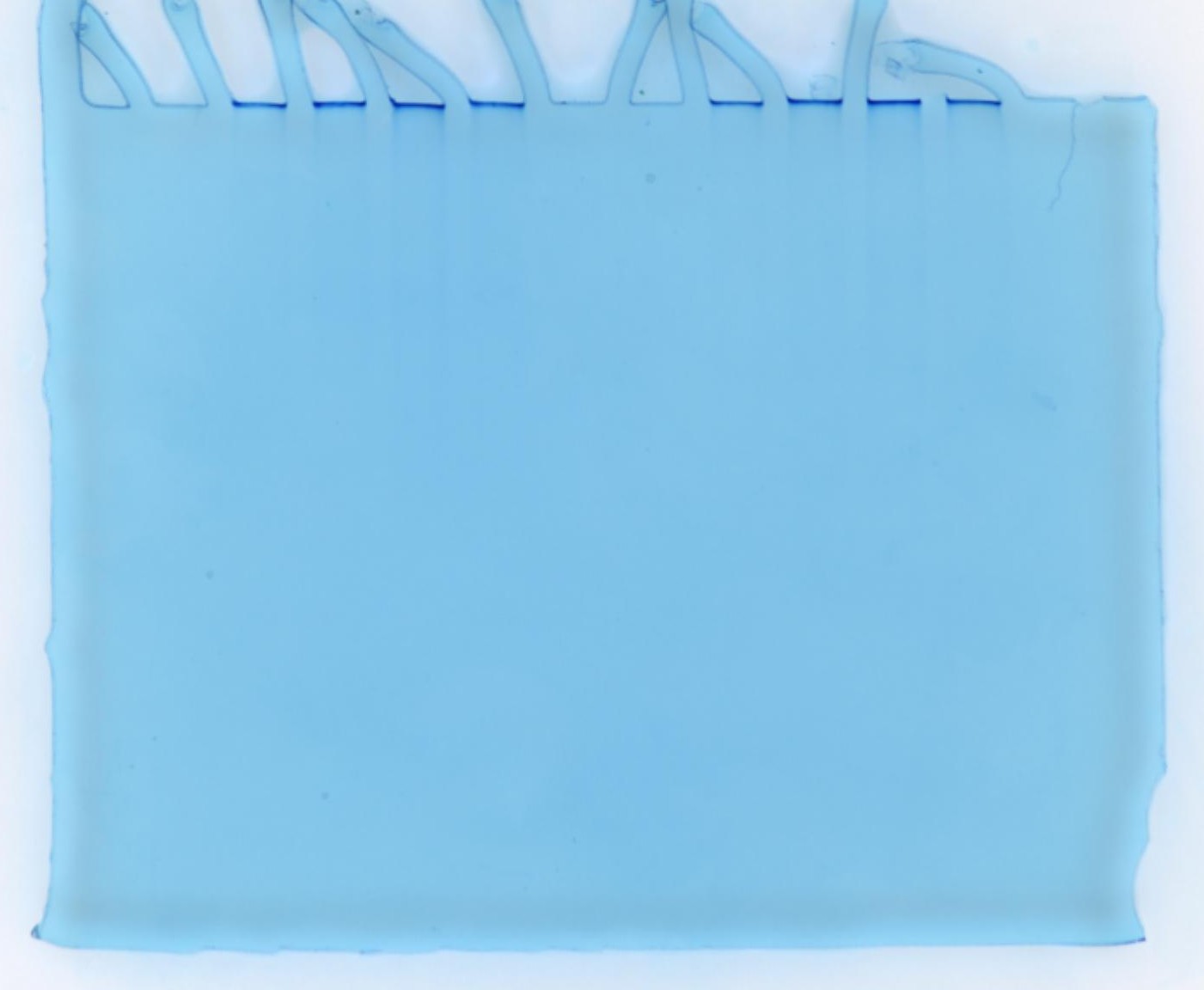

Supplement: Figure 4—figure supplement 2—source data 2. [file elife-101717-fig4-figsupp2-data2.zip › B/sld3 1-2,5-3.jpg]

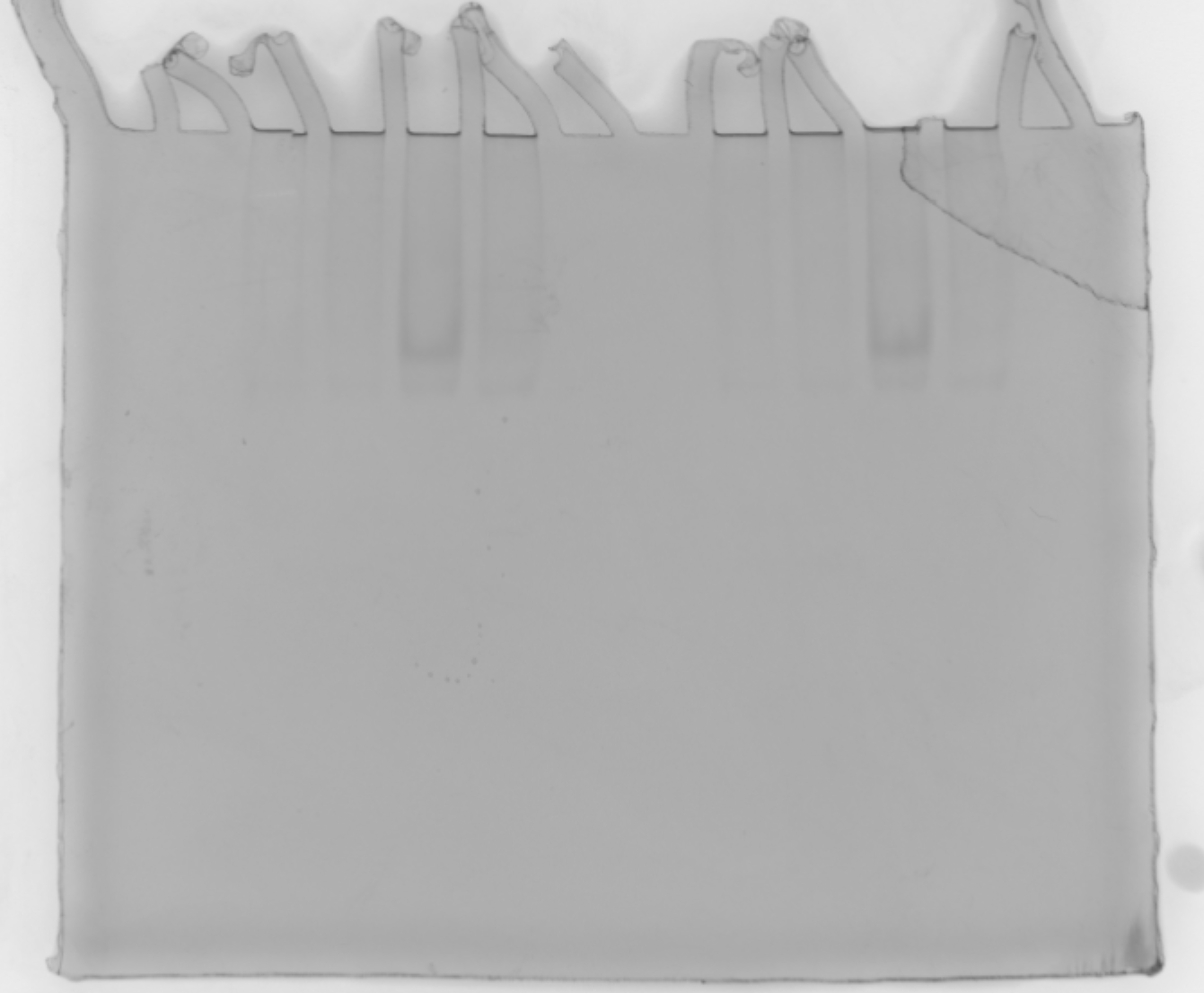

Supplement: Figure 4—figure supplement 2—source data 2. [file elife-101717-fig4-figsupp2-data2.zip › B/sld3sld7 1-2,5-3.tif]

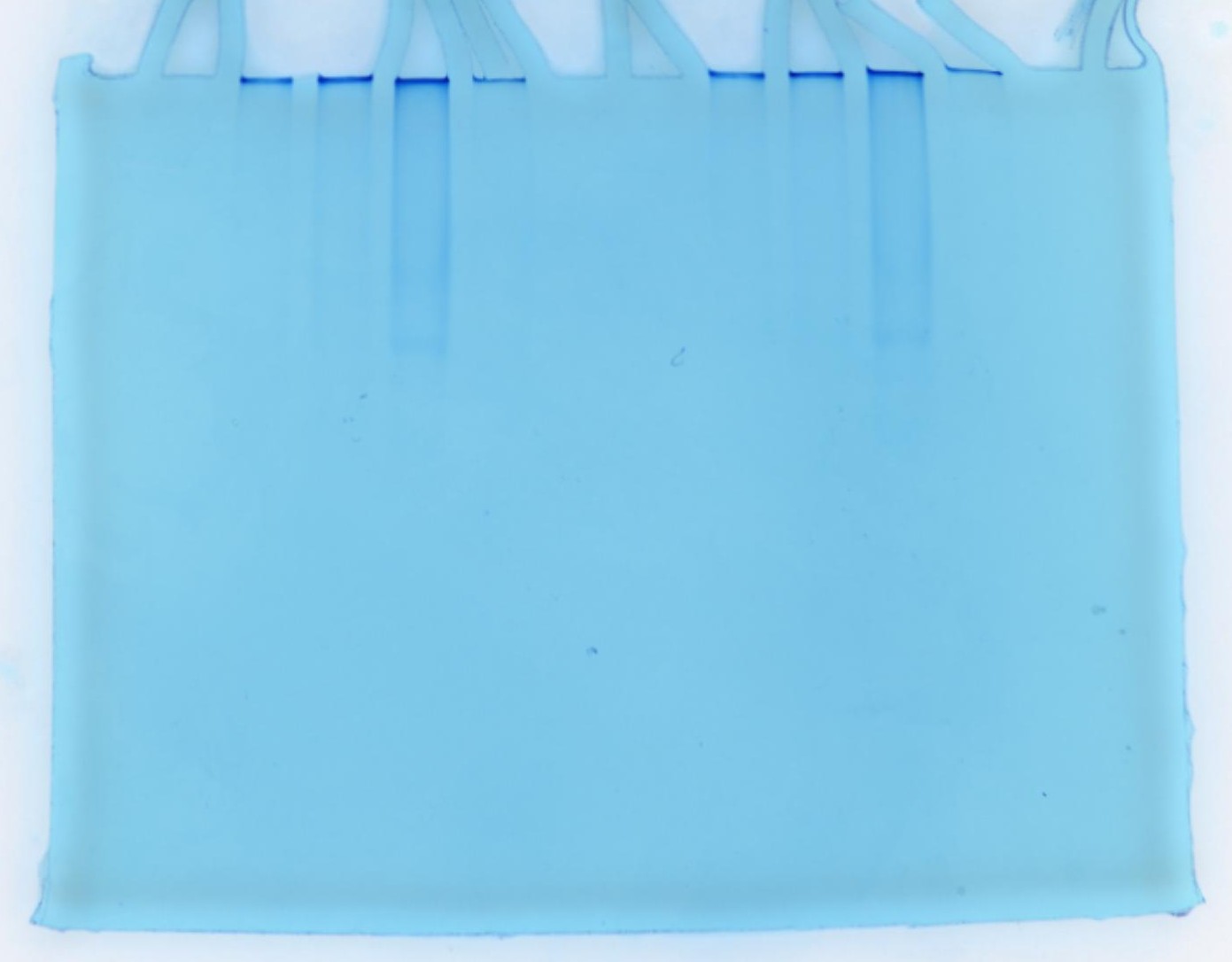

Supplement: Figure 4—figure supplement 2—source data 2. [file elife-101717-fig4-figsupp2-data2.zip › B/sld3cdc45 1-5-1,2.jpg]

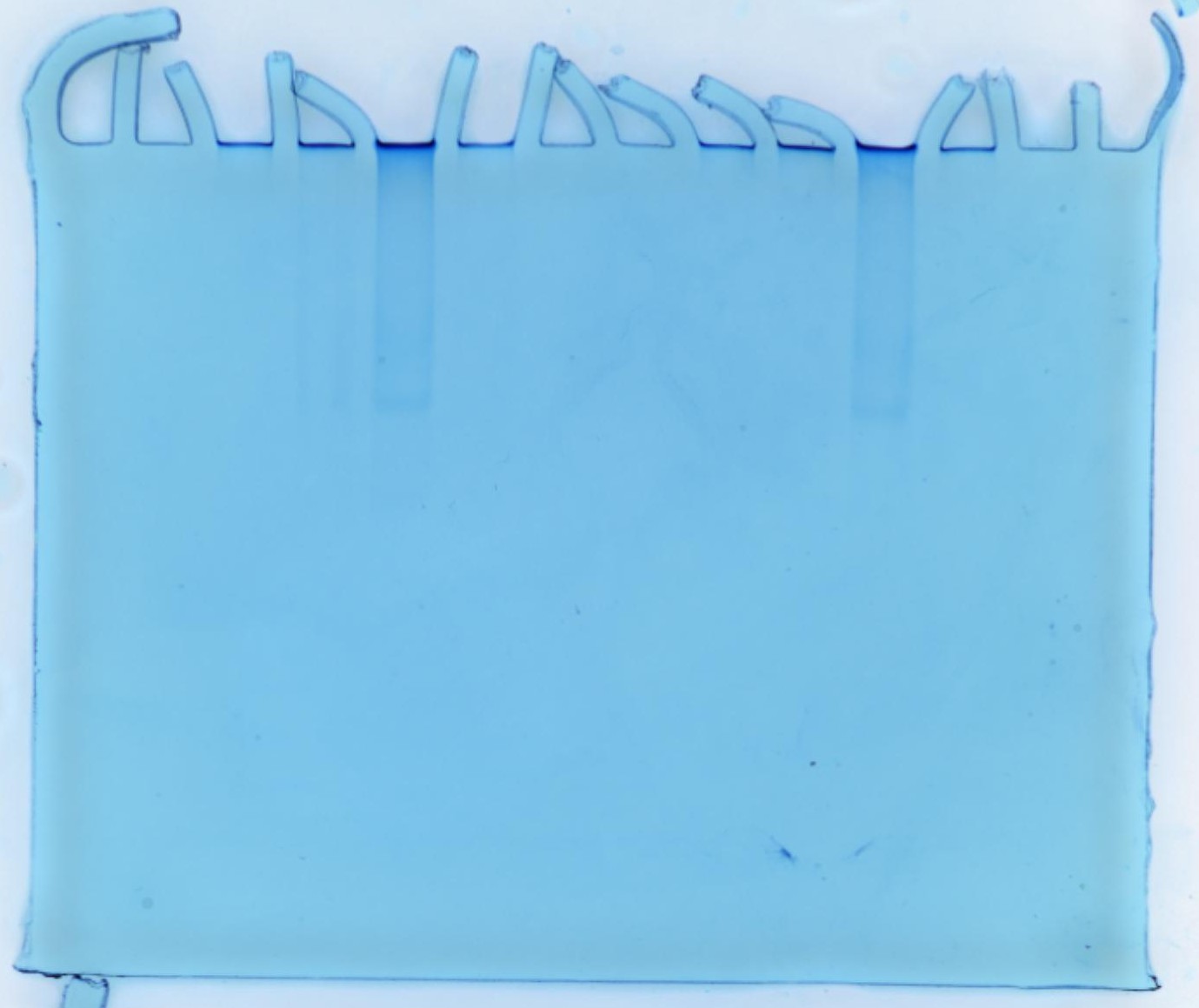

Supplement: Figure 4—figure supplement 2—source data 2. [file elife-101717-fig4-figsupp2-data2.zip › B/sld3cdc45 1-2,5-3.jpg]

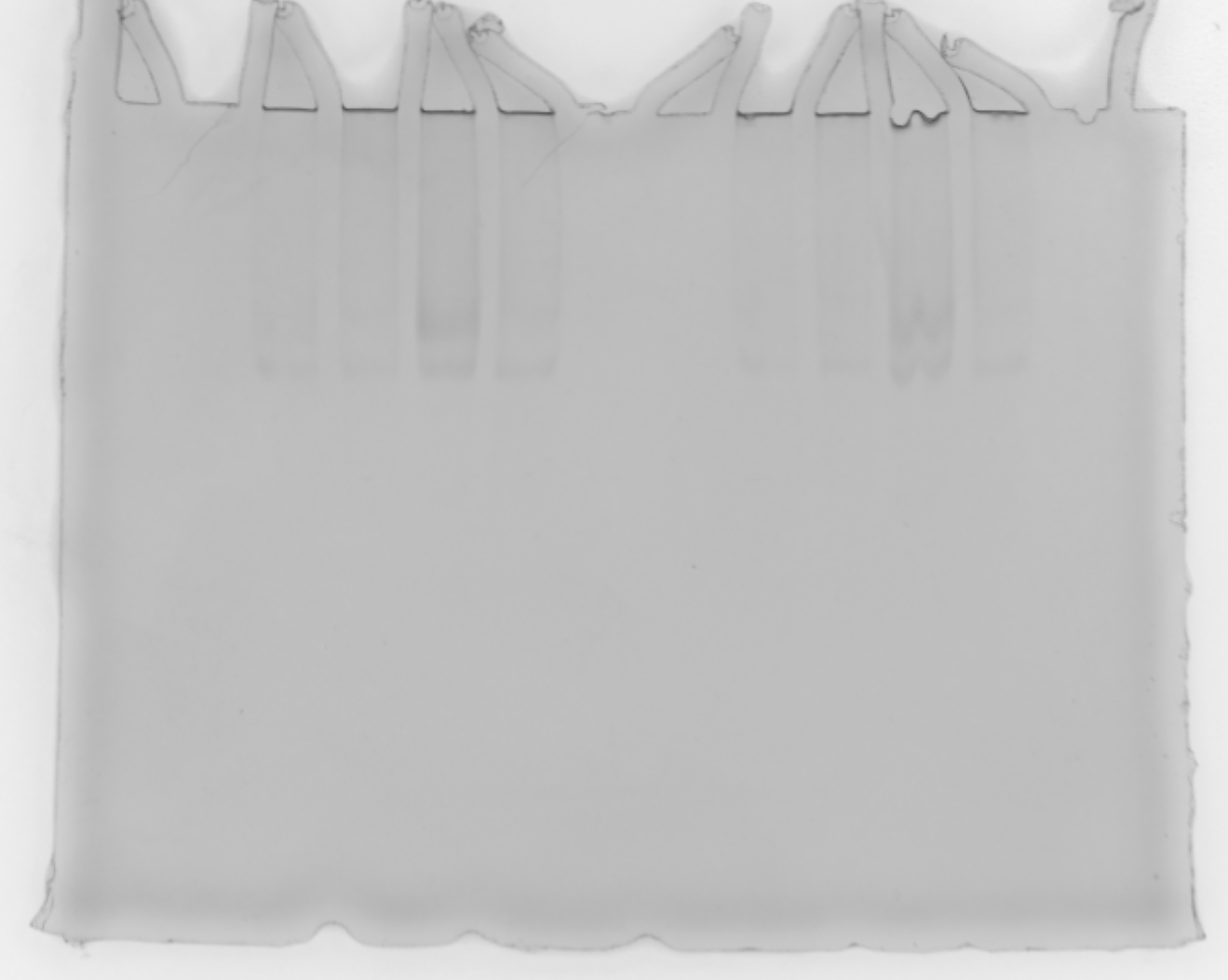

Supplement: Figure 4—figure supplement 2—source data 2. [file elife-101717-fig4-figsupp2-data2.zip › B/sld3sld7 1-5-1,2.tif]

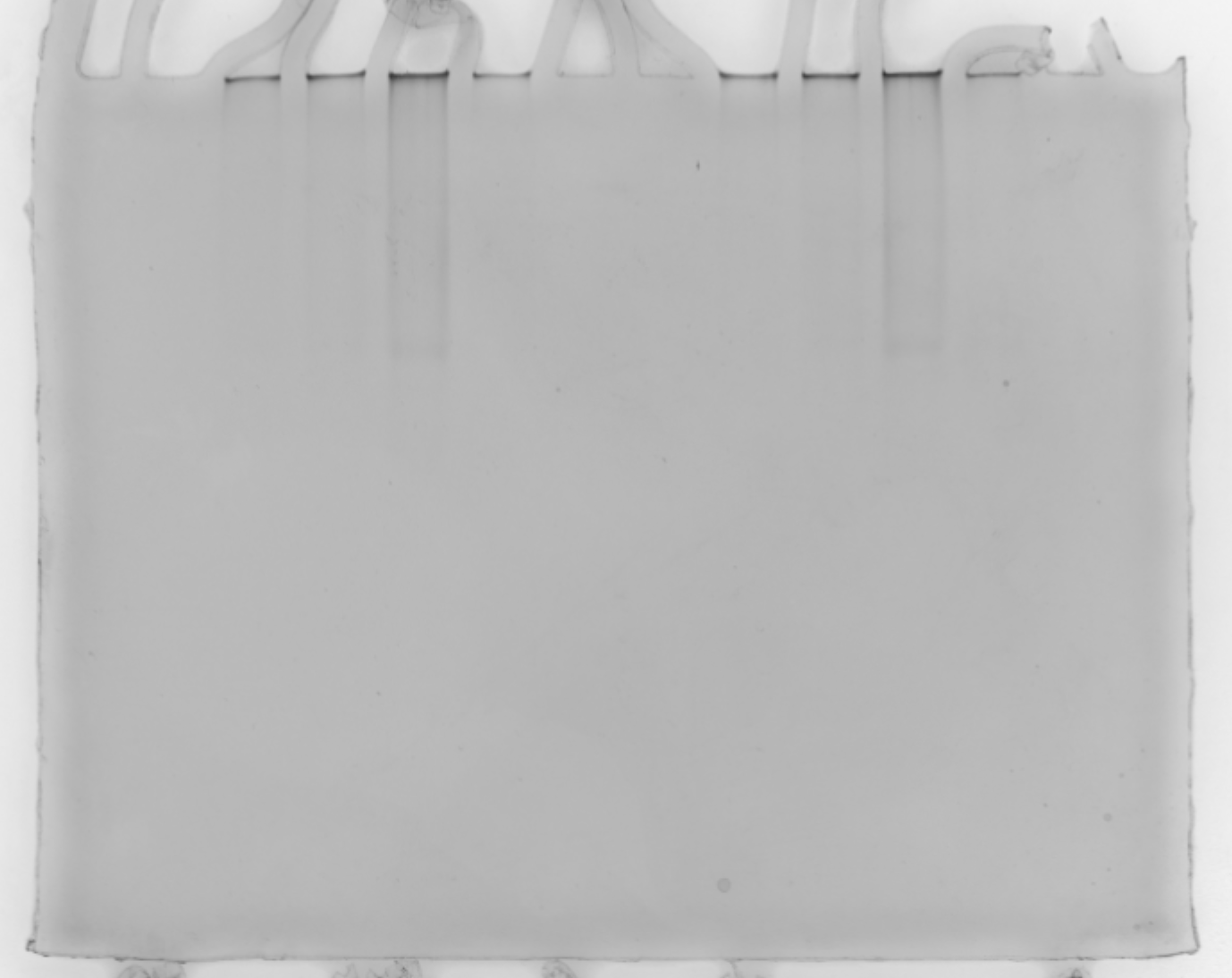

Supplement: Figure 4—figure supplement 2—source data 2. [file elife-101717-fig4-figsupp2-data2.zip › B/sld3cdc45 1-2-1,2.tif]

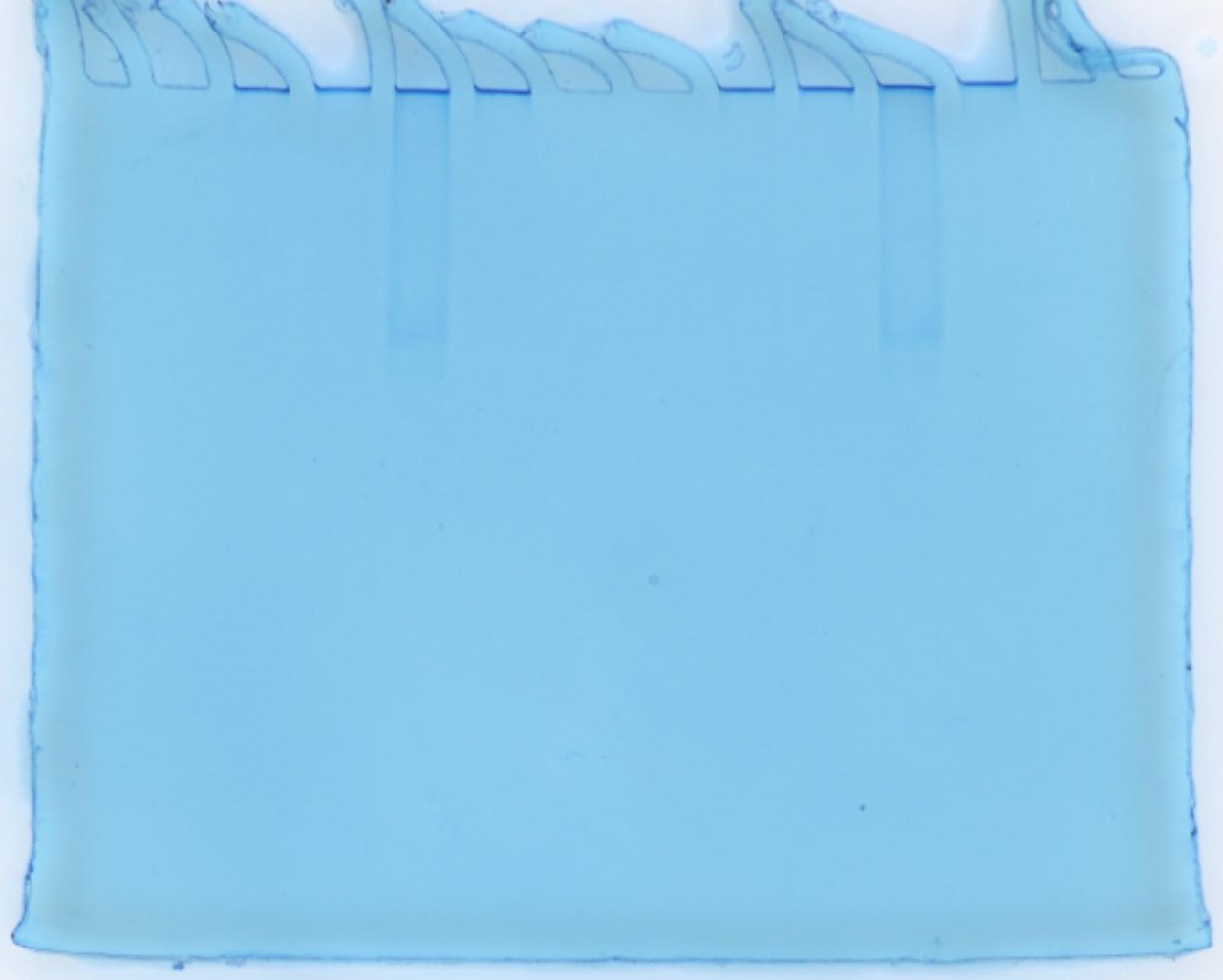

Supplement: Figure 4—figure supplement 2—source data 2. [file elife-101717-fig4-figsupp2-data2.zip › B/sld3sld7 1-2-1,2.jpg]

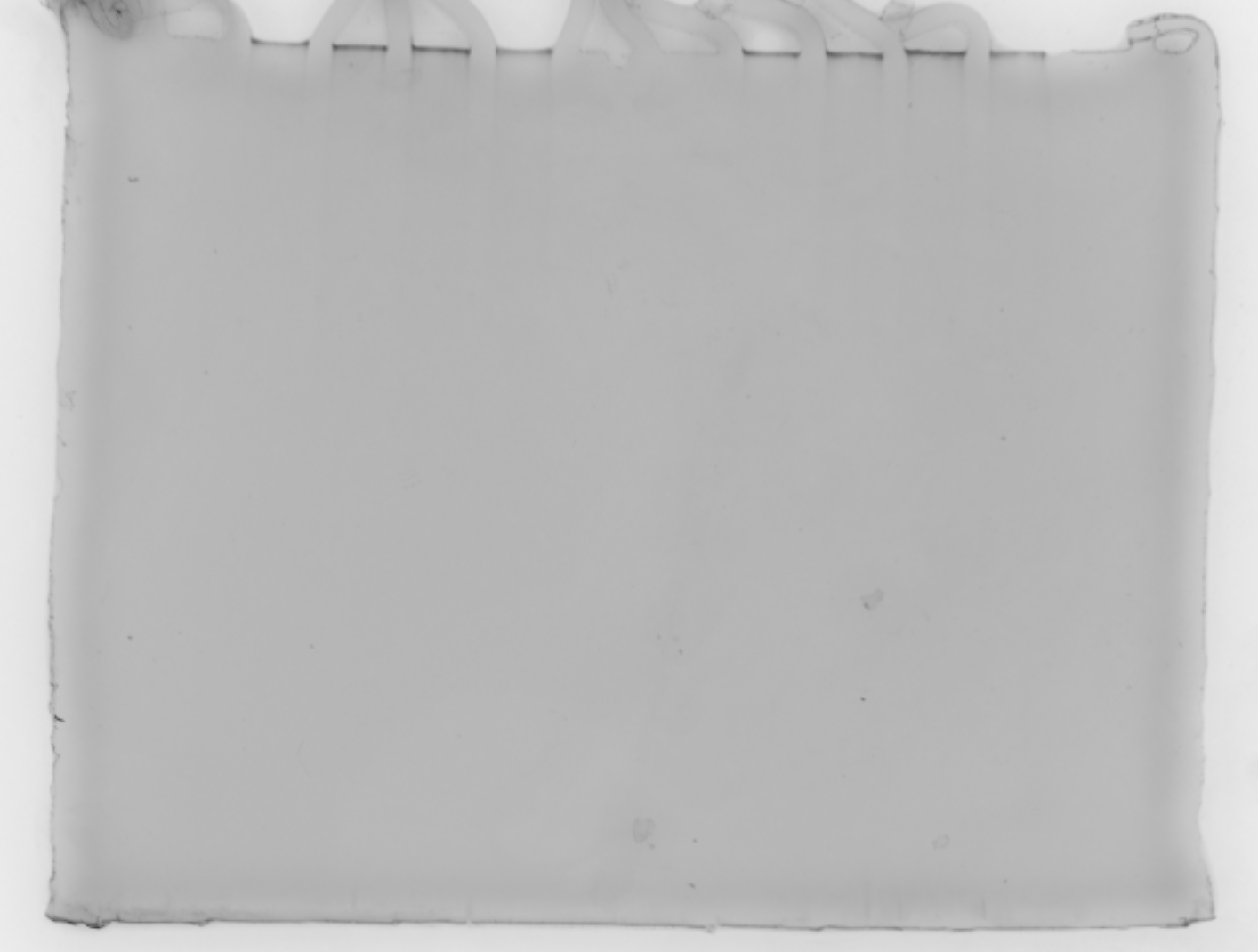

Supplement: Figure 4—figure supplement 2—source data 2. [file elife-101717-fig4-figsupp2-data2.zip › B/sld3 1-2-1,2.tif]

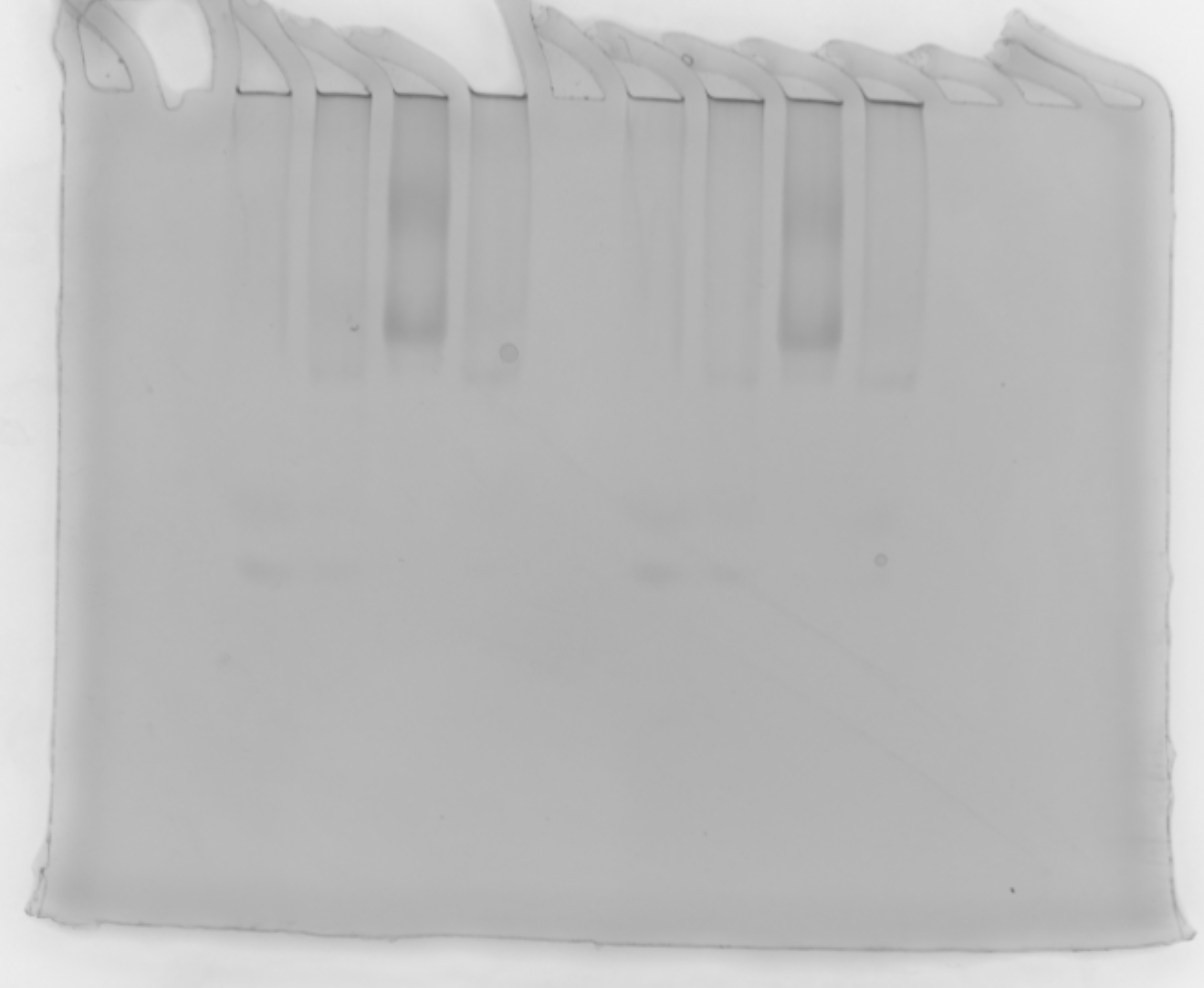

Supplement: Figure 4—figure supplement 2—source data 2. [file elife-101717-fig4-figsupp2-data2.zip › B/Sld7Sld3Cdc45 1-2-1,2.tif]

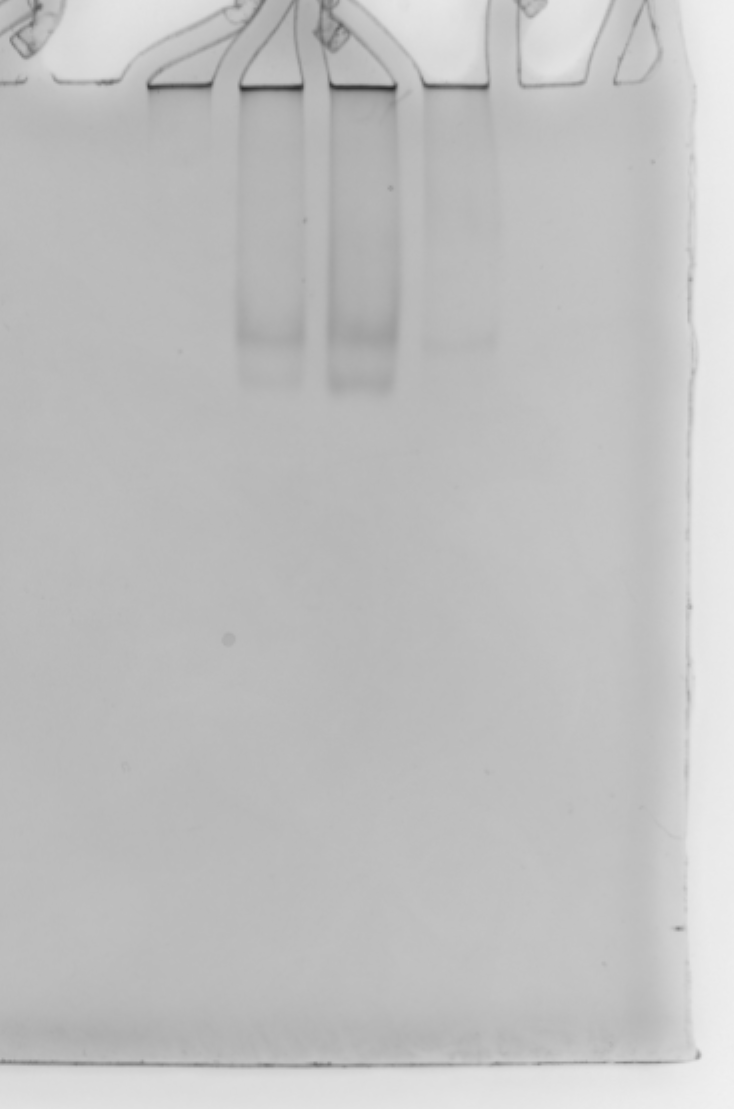

Supplement: Figure 4—figure supplement 2—source data 2. [file elife-101717-fig4-figsupp2-data2.zip › B/Sld3Sld7Cdc45 1-2-3.tif]

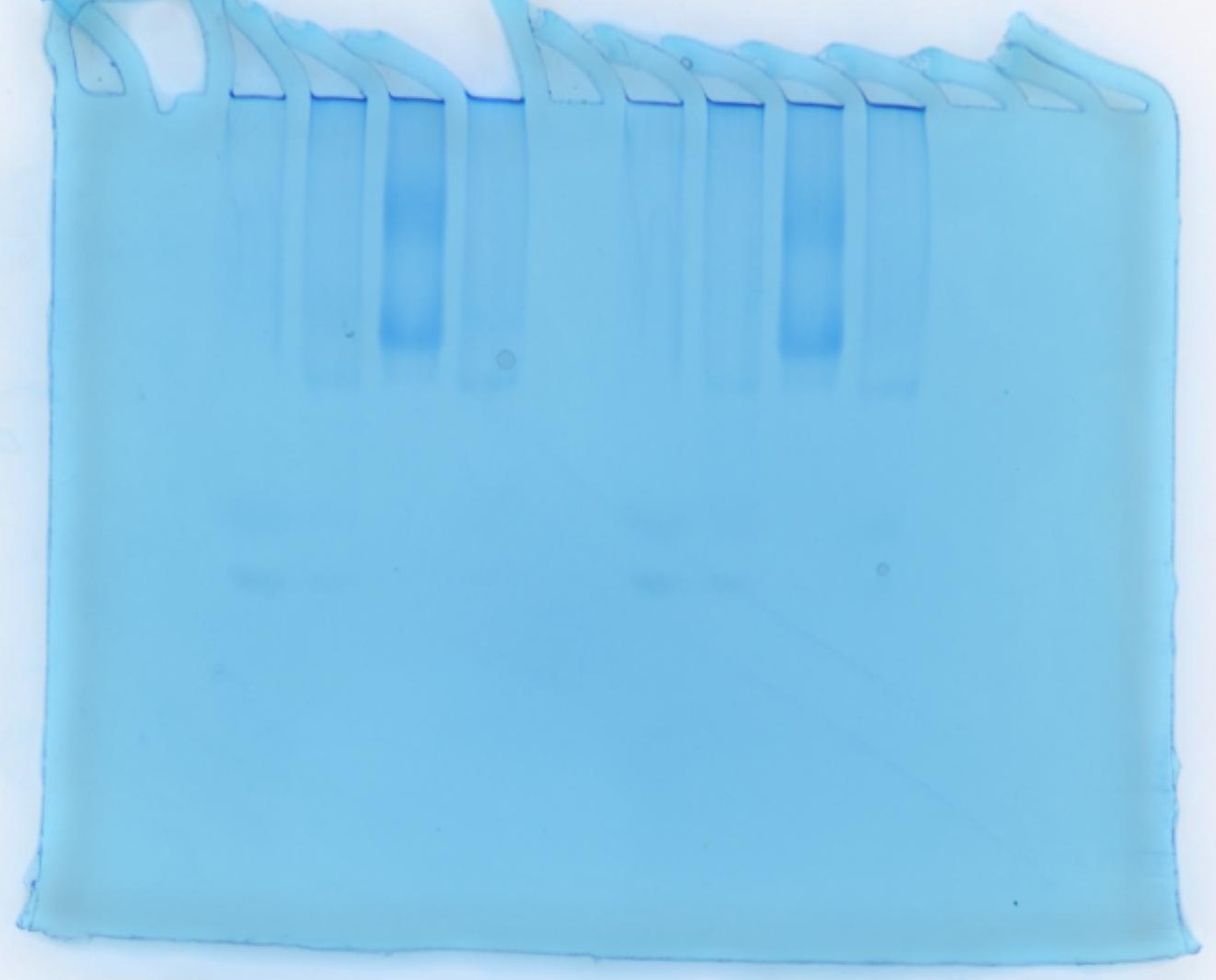

Supplement: Figure 4—figure supplement 2—source data 2. [file elife-101717-fig4-figsupp2-data2.zip › B/Sld7Sld3Cdc45 1-2-1,2.jpg]

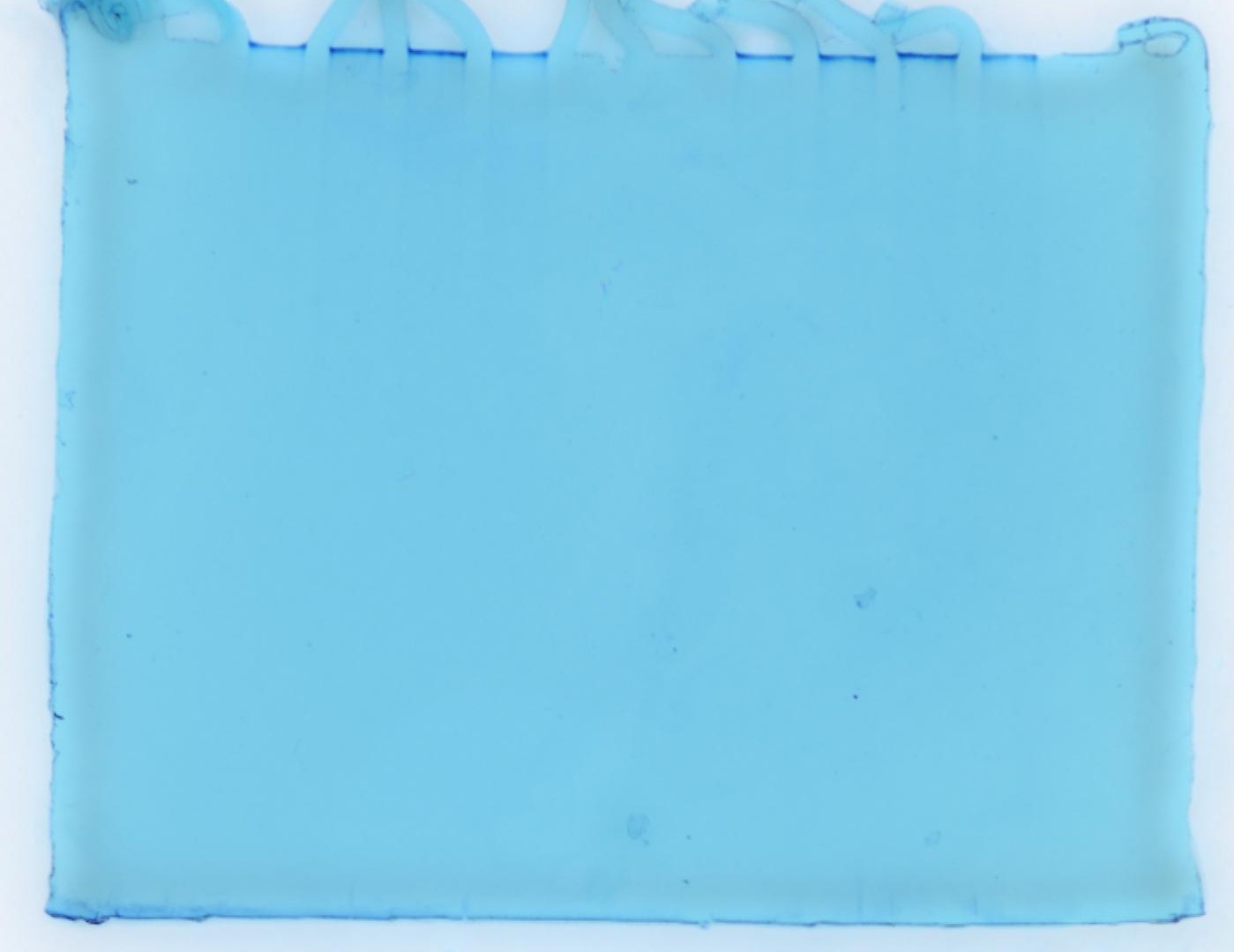

Supplement: Figure 4—figure supplement 2—source data 2. [file elife-101717-fig4-figsupp2-data2.zip › B/sld3 1-2-1,2.jpg]

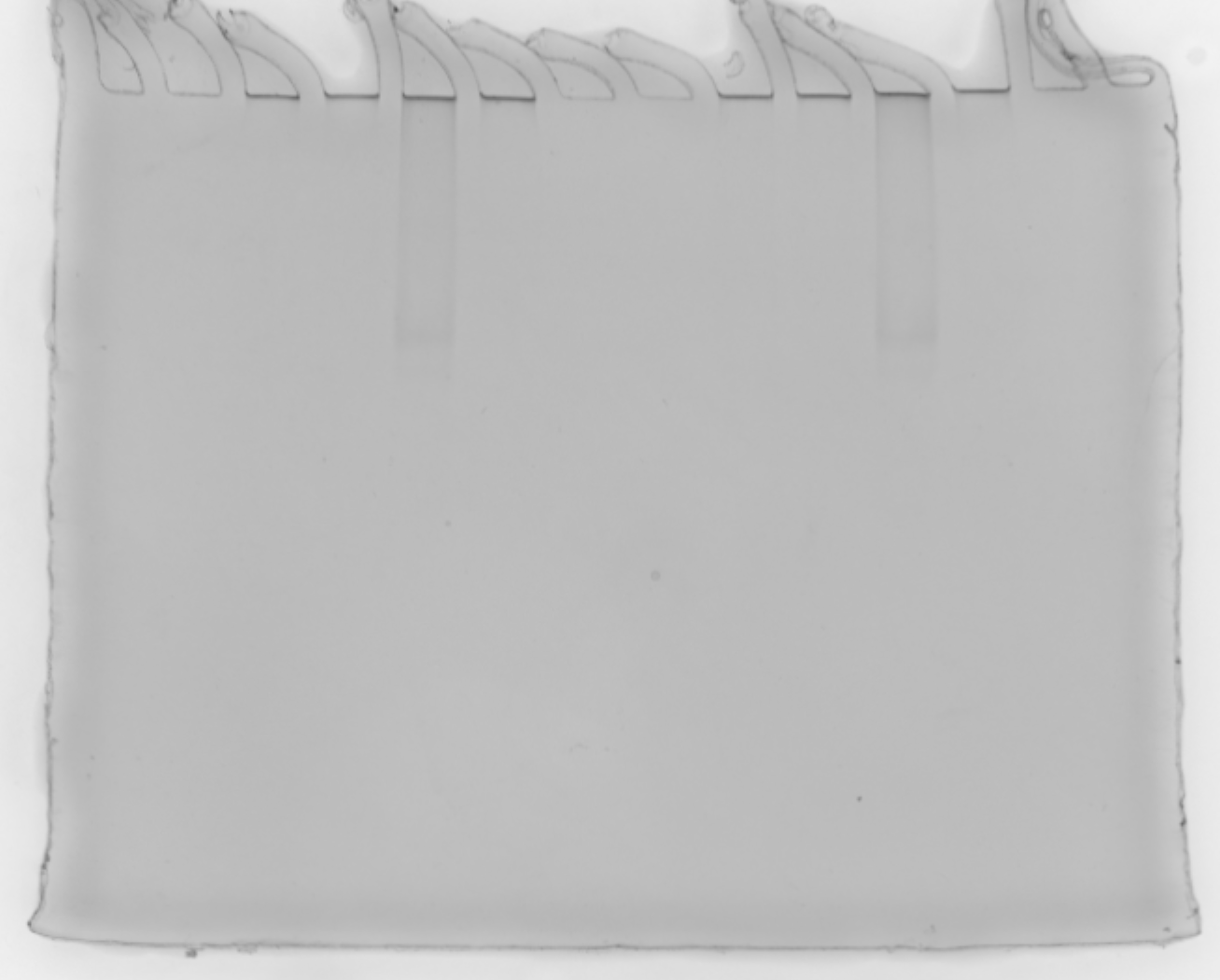

Supplement: Figure 4—figure supplement 2—source data 2. [file elife-101717-fig4-figsupp2-data2.zip › B/sld3sld7 1-2-1,2.tif]

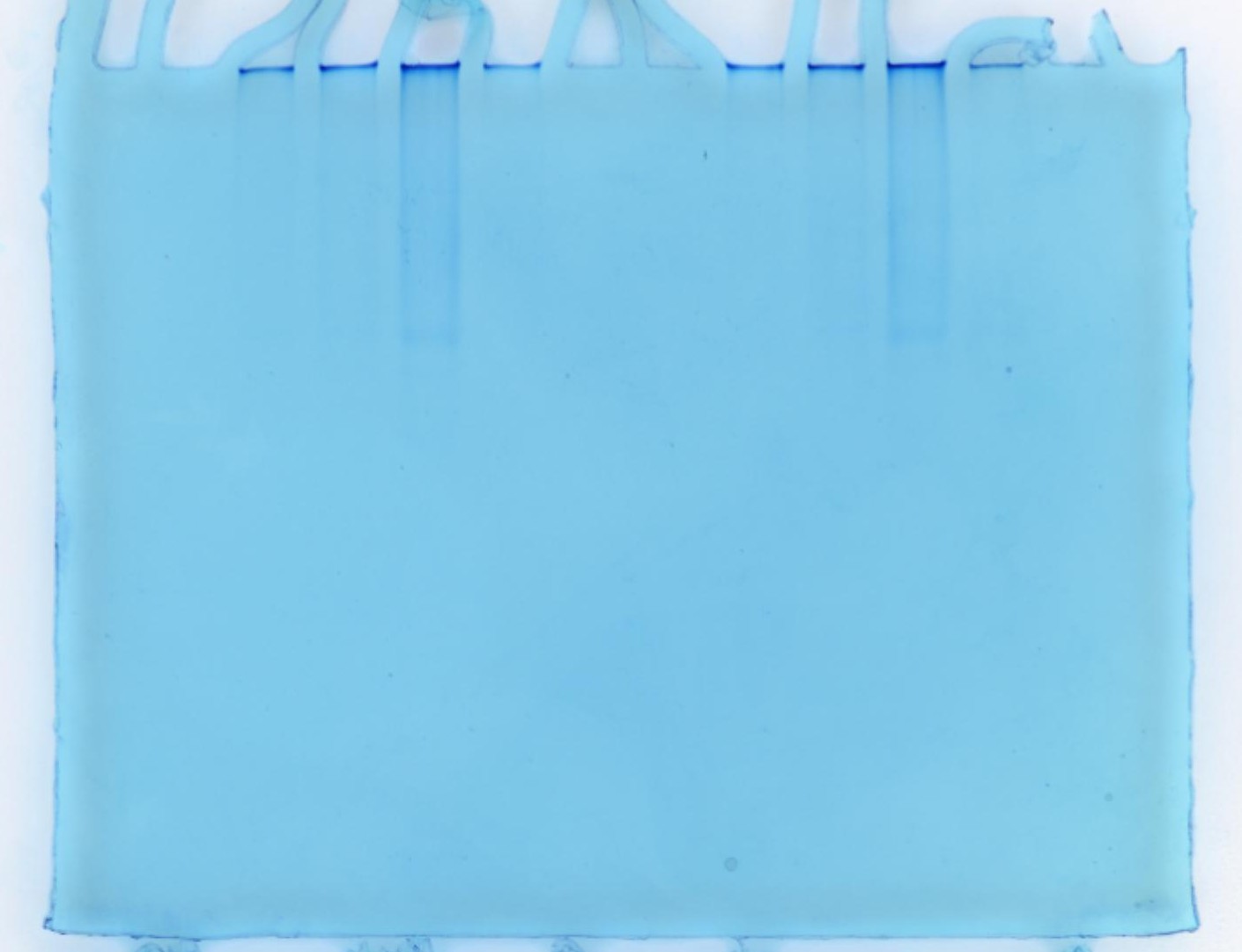

Supplement: Figure 4—figure supplement 2—source data 2. [file elife-101717-fig4-figsupp2-data2.zip › B/sld3cdc45 1-2-1,2.jpg]

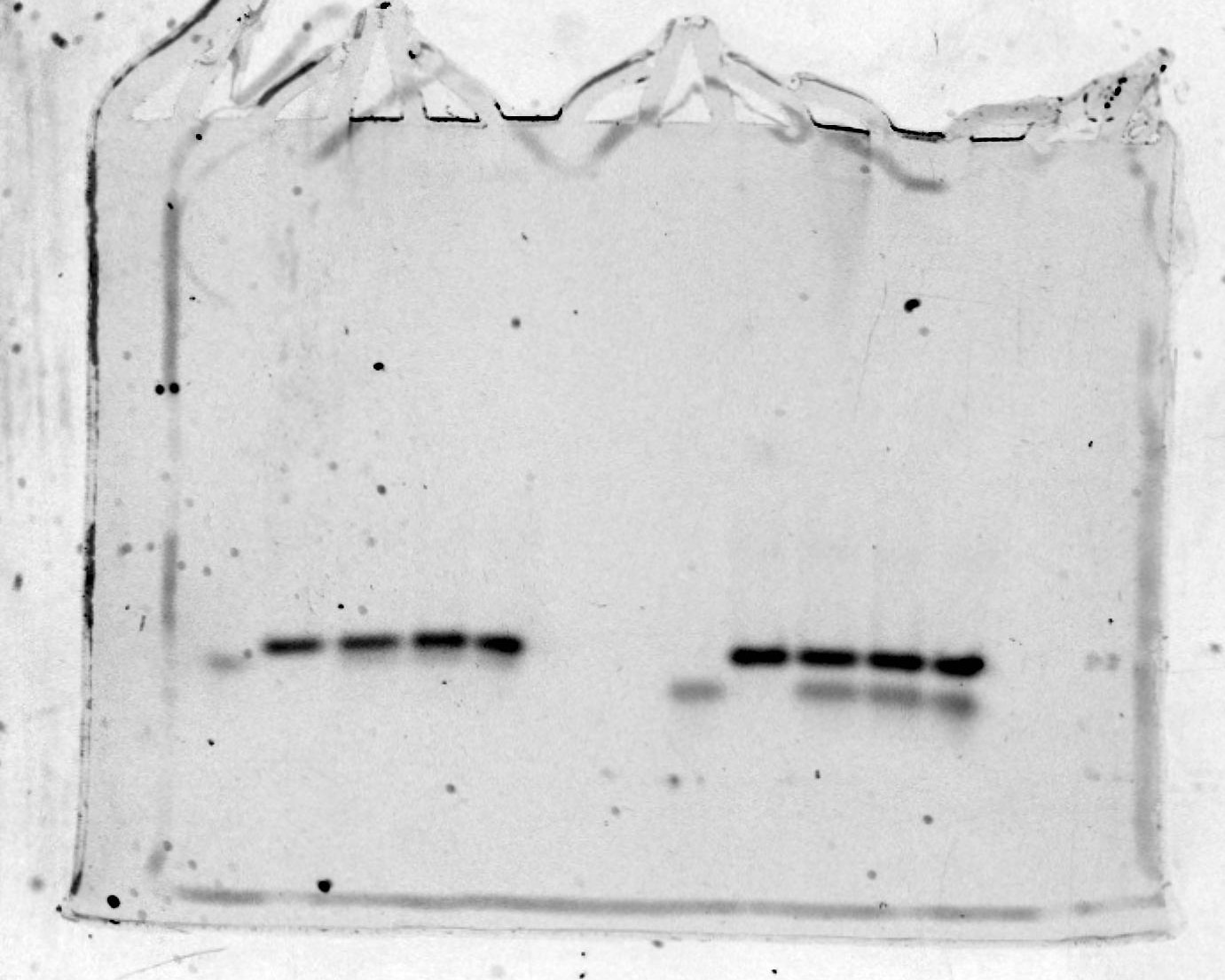

Supplement: Figure 4—figure supplement 3—source data 2. [file elife-101717-fig4-figsupp3-data2.zip › Sld3 1-3+ds SYBR 2024.03.23_22.00.51_Fl-UV.jpg]

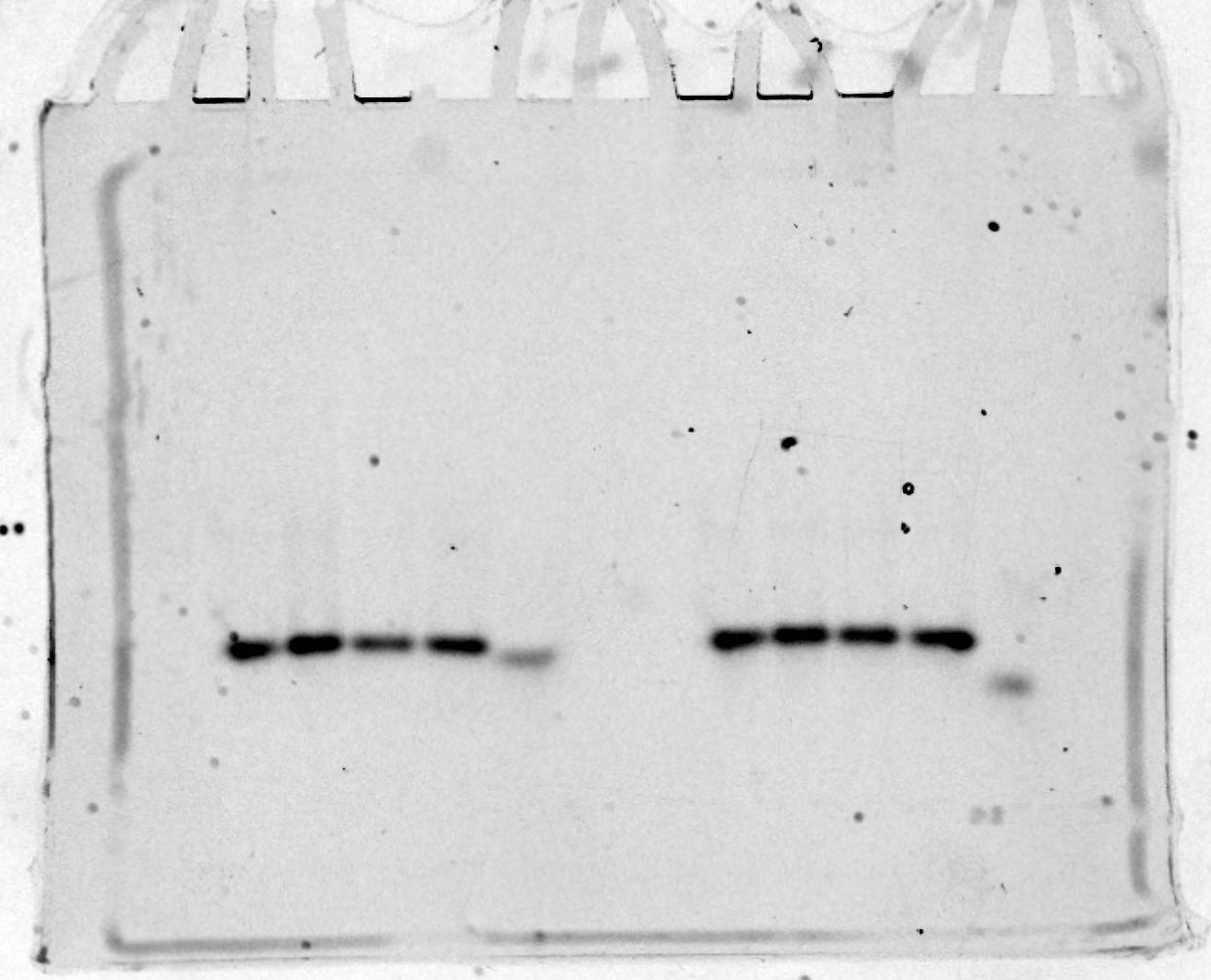

Supplement: Figure 4—figure supplement 3—source data 2. [file elife-101717-fig4-figsupp3-data2.zip › Sld3 1-25+ds SYBR 2024.03.23_21.57.48_Fl-UV.jpg]

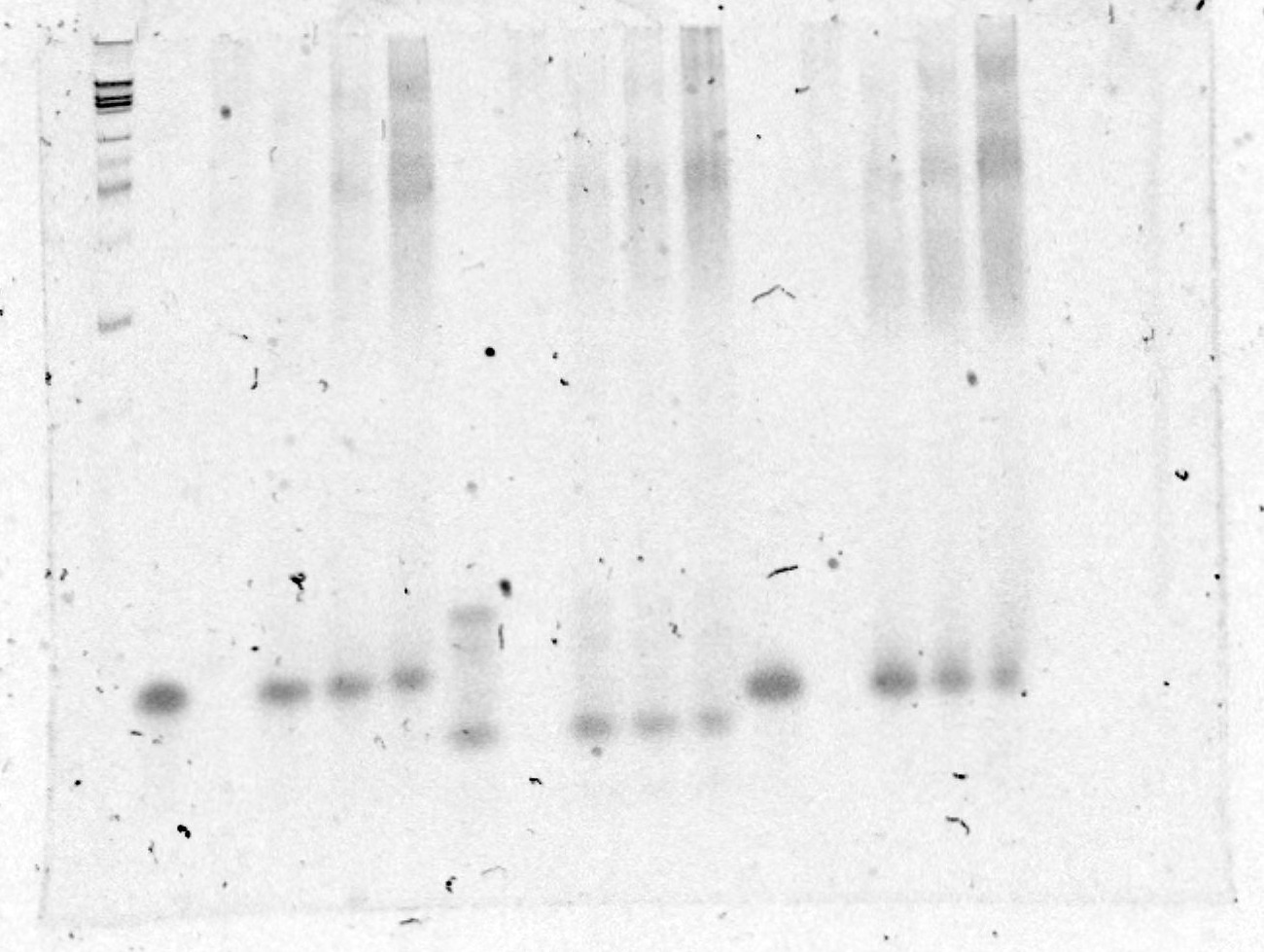

Supplement: Figure 4—figure supplement 3—source data 2. [file elife-101717-fig4-figsupp3-data2.zip › Sld3CBDCdc45 to 1-1 2019.07.04_14.39.33_Fl-UV.jpg]

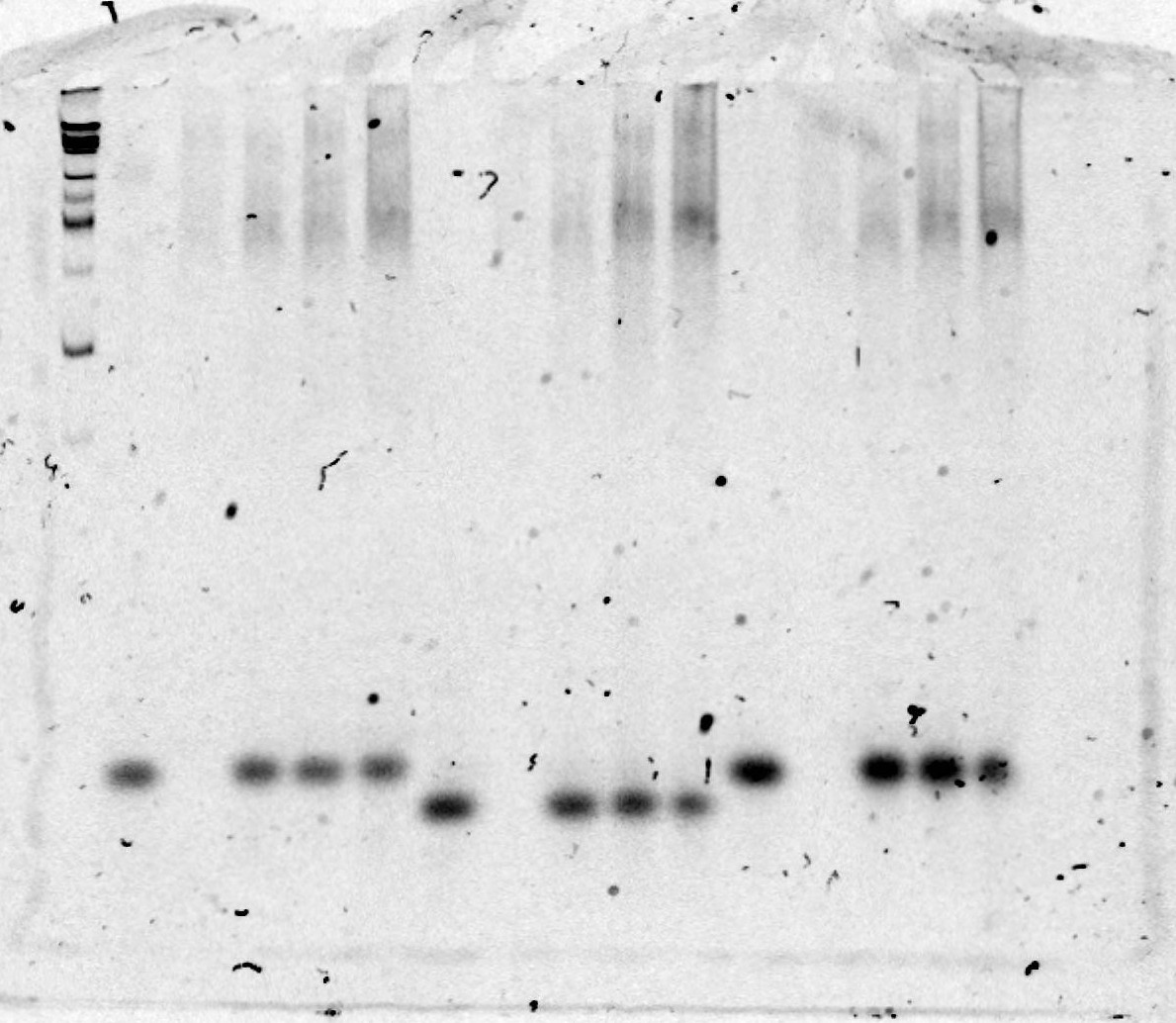

Supplement: Figure 4—figure supplement 3—source data 2. [file elife-101717-fig4-figsupp3-data2.zip › Sld3CBDCdc45 to 1-3 2019.07.05_12.42.55_Fl-UV.jpg]

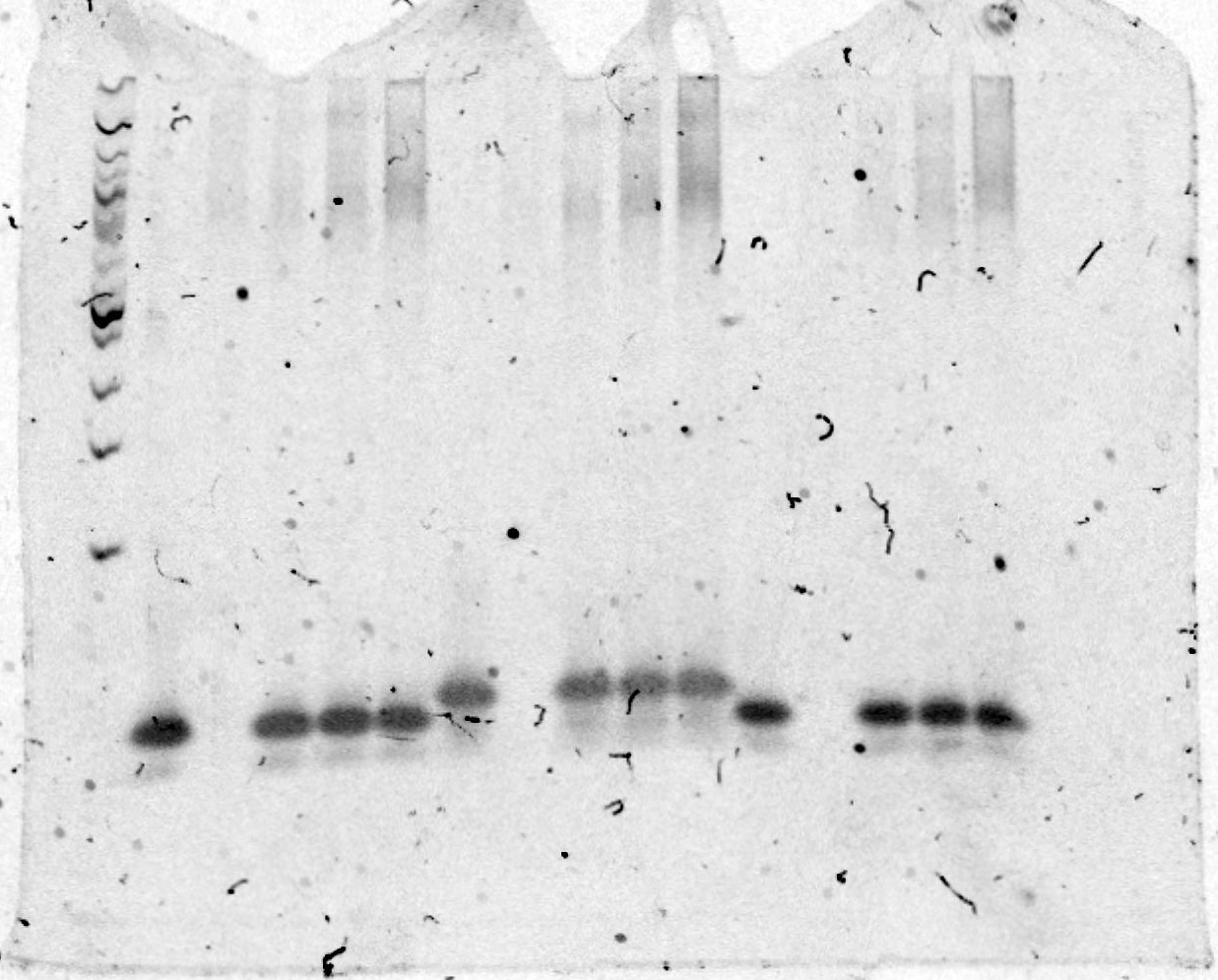

Supplement: Figure 4—figure supplement 3—source data 2. [file elife-101717-fig4-figsupp3-data2.zip › Sld3CBDCdc45 to 1-4 2019.07.17_12.19.09_Fl-UV.jpg]

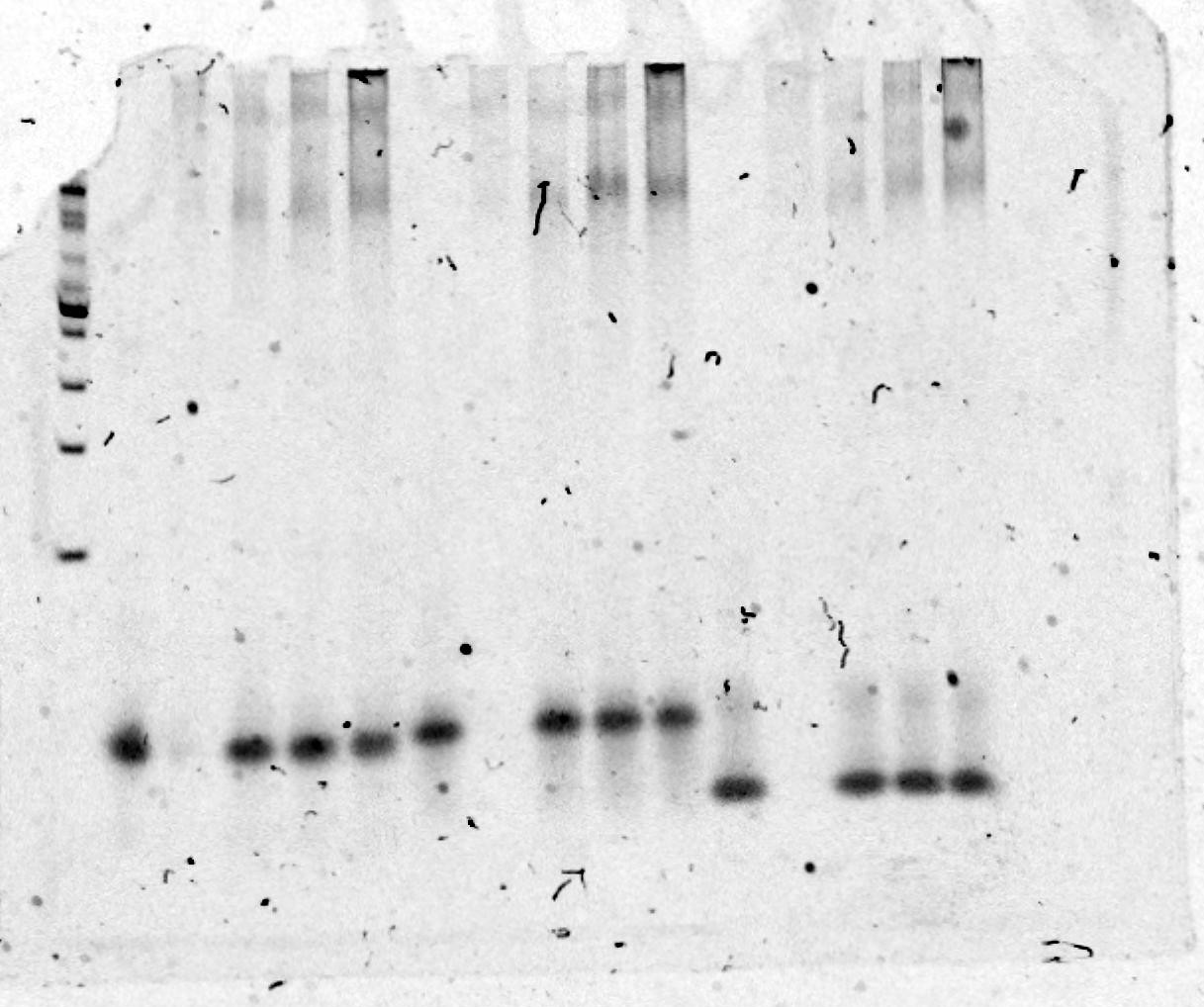

Supplement: Figure 4—figure supplement 3—source data 2. [file elife-101717-fig4-figsupp3-data2.zip › Sld3CBDCdc45 to 1-6 2019.07.16_12.03.37_Fl-UV.jpg]

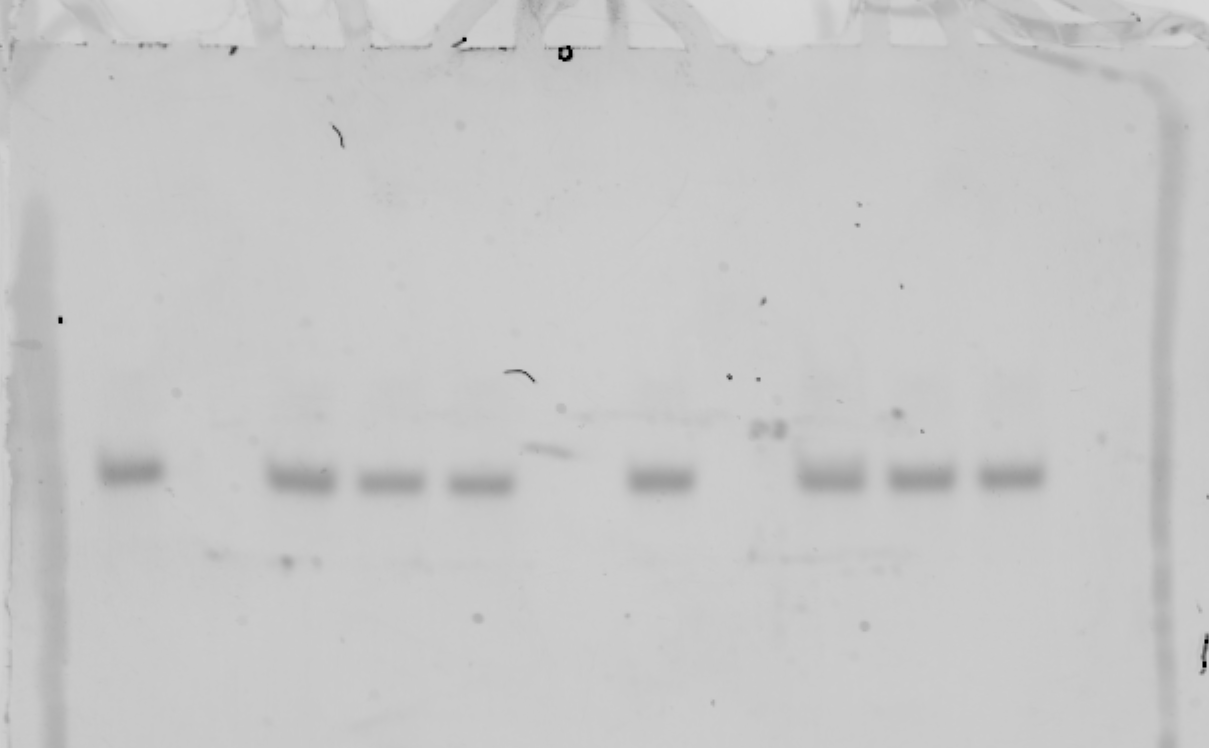

Supplement: Figure 4—figure supplement 3—source data 2. [file elife-101717-fig4-figsupp3-data2.zip › Sld7Sld3Cdc45 to 1-1-1,2.tif]

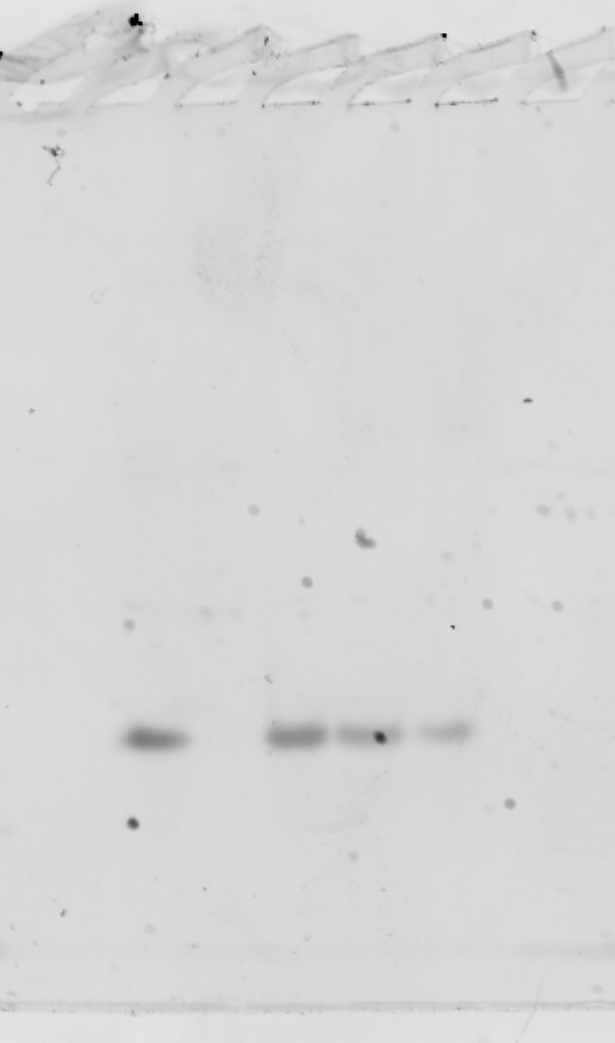

Supplement: Figure 4—figure supplement 3—source data 2. [file elife-101717-fig4-figsupp3-data2.zip › Sld7Sld3Cdc45 to 1-1-3.tif]

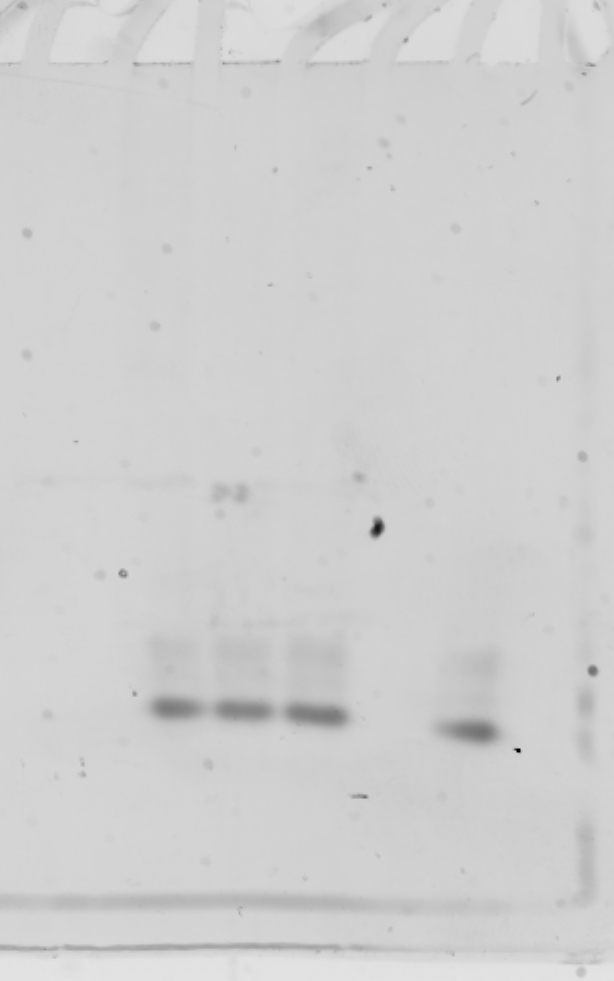

Supplement: Figure 4—figure supplement 3—source data 2. [file elife-101717-fig4-figsupp3-data2.zip › Sld7Sld3Cdc45 to 1-3-1.tif]

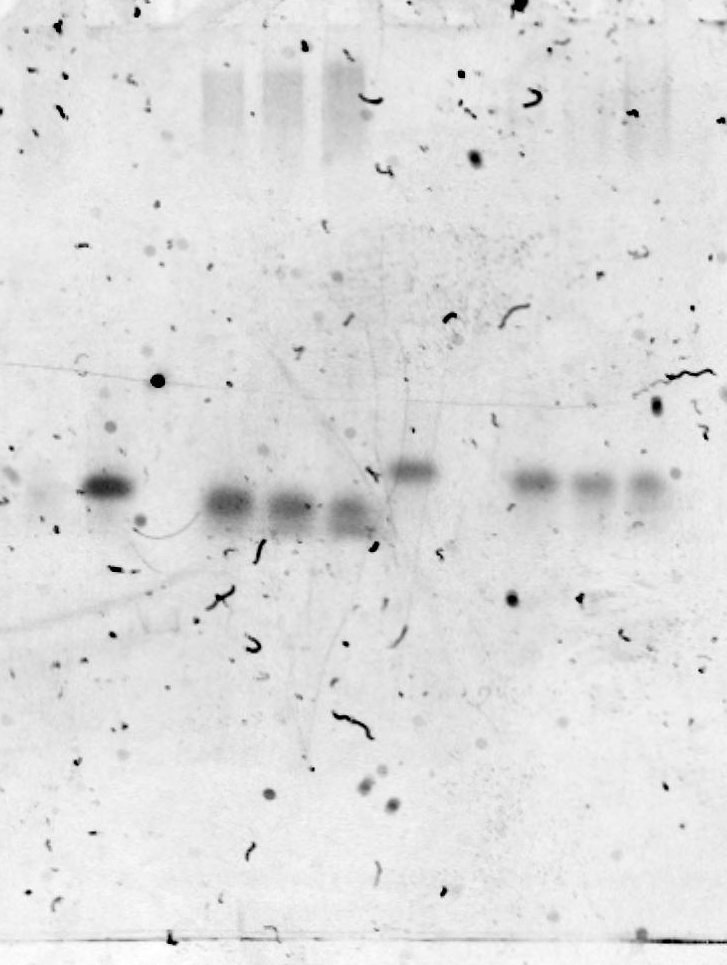

Supplement: Figure 4—figure supplement 3—source data 2. [file elife-101717-fig4-figsupp3-data2.zip › Sld7Sld3Cdc45 to 1-3-2,3.tiff]

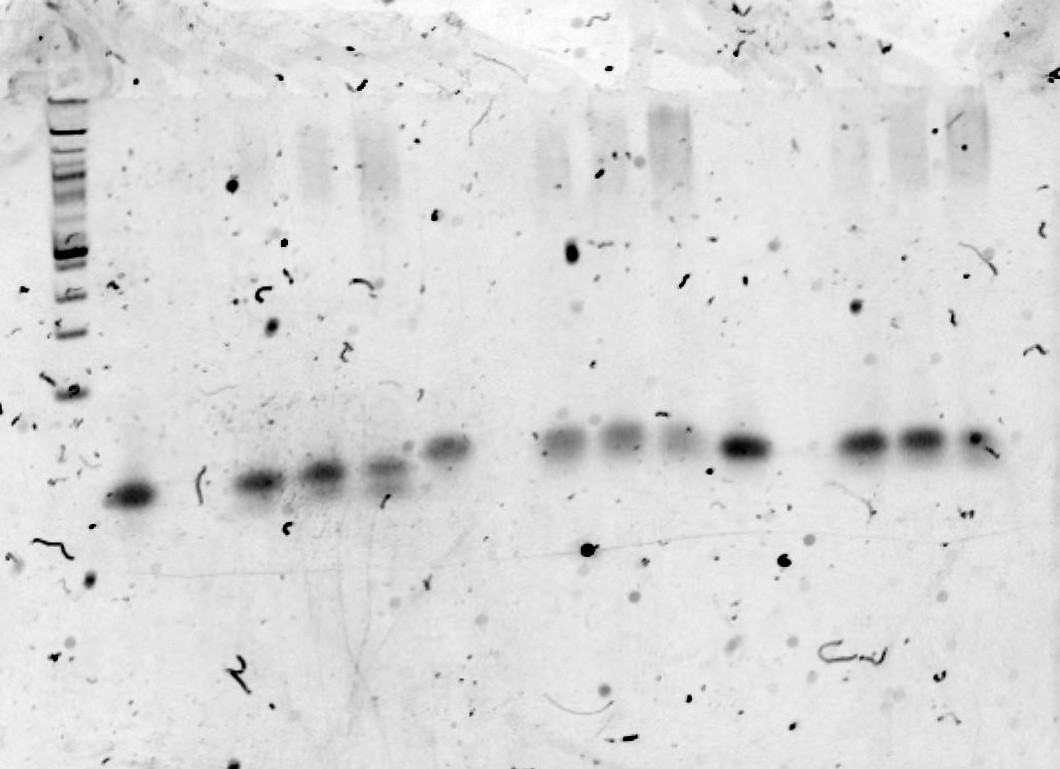

Supplement: Figure 4—figure supplement 3—source data 2. [file elife-101717-fig4-figsupp3-data2.zip › Sld7Sld3Cdc45 to 1-4-1,2,3.jpg]

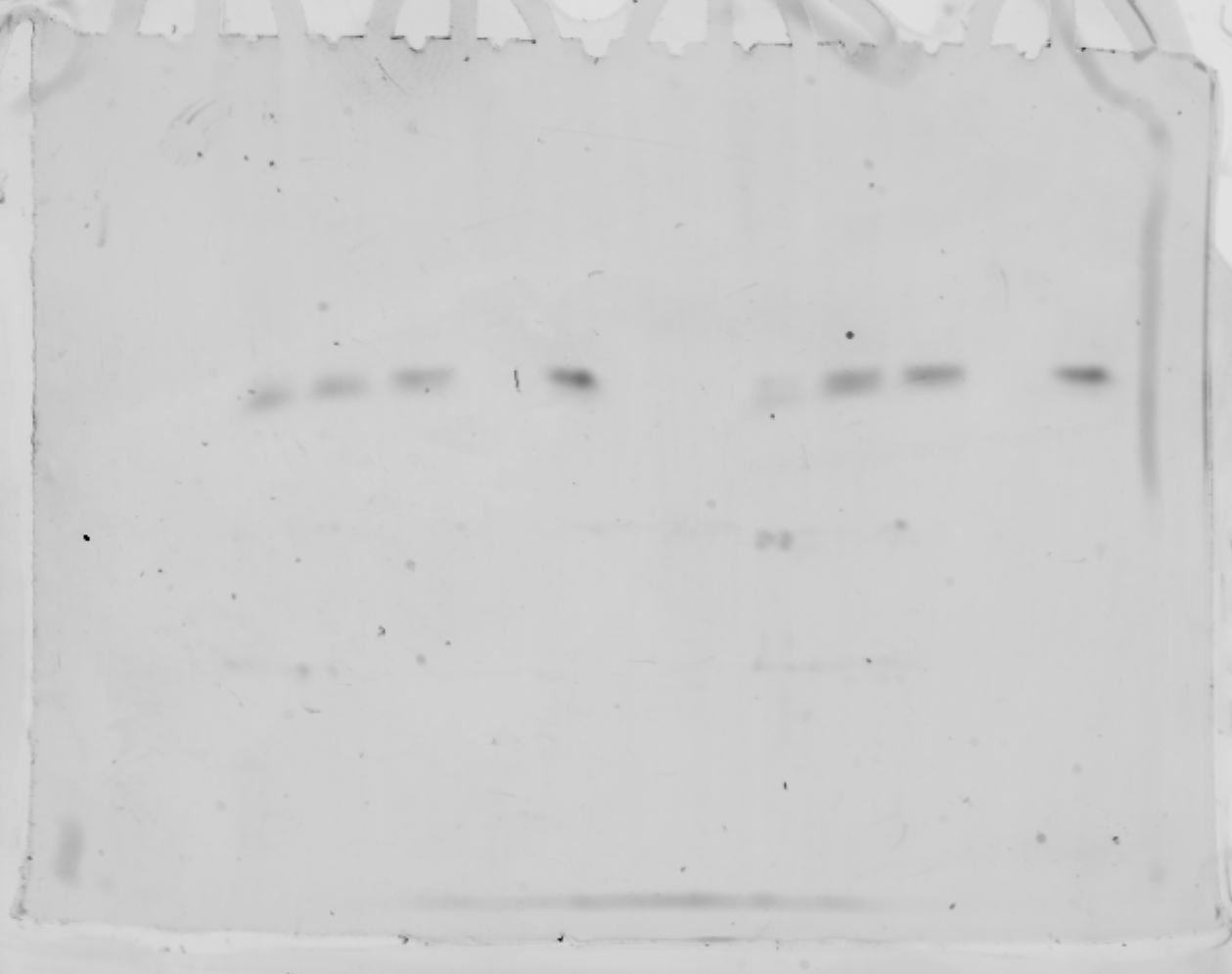

Supplement: Figure 4—figure supplement 3—source data 2. [file elife-101717-fig4-figsupp3-data2.zip › Sld7Sld3Cdc45 to 1-6-1,2.tif]

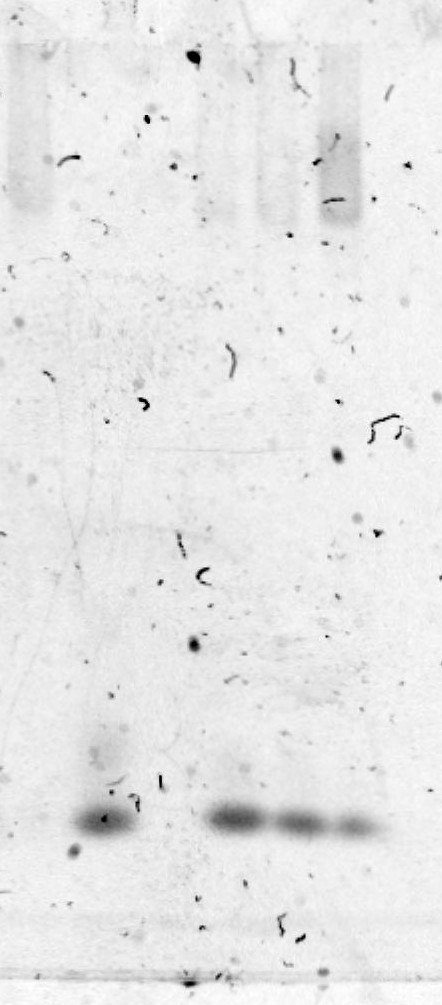

Supplement: Figure 4—figure supplement 3—source data 2. [file elife-101717-fig4-figsupp3-data2.zip › Sld7Sld3Cdc45 to 1-6-3.jpg]
