# Supplementary material for: Sld3CBD–Cdc45 structural insights into Cdc45 recruitment for CMG complex formation during DNA replication
Source: eLife. 2025 Sep 8;13:RP101717. doi: 10.7554/eLife.101717 (PMC12416888; doi:10.7554/eLife.101717)
Supplement: Figure 2—figure supplement 3—source data 1. [file elife-101717-fig2-figsupp3-data1.pdf]

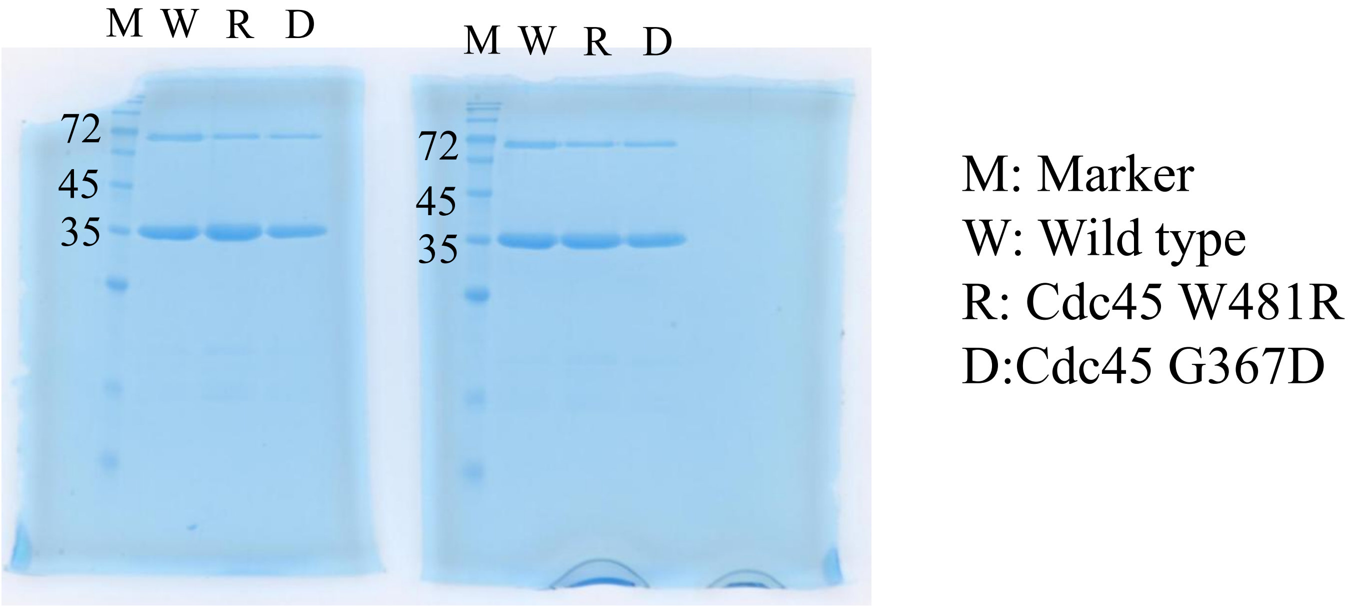

Figure2-figure supplement3, Source Data 1. Original SDS-PAGE corresponding to Figure2-figure supplement3. The right panel shows a biological replicate performed concurrently.
