# Supplementary material for: Sld3CBD–Cdc45 structural insights into Cdc45 recruitment for CMG complex formation during DNA replication
Source: eLife. 2025 Sep 8;13:RP101717. doi: 10.7554/eLife.101717 (PMC12416888; doi:10.7554/eLife.101717)
Supplement: Figure 4—figure supplement 3—source data 1. [file elife-101717-fig4-figsupp3-data1.pdf]

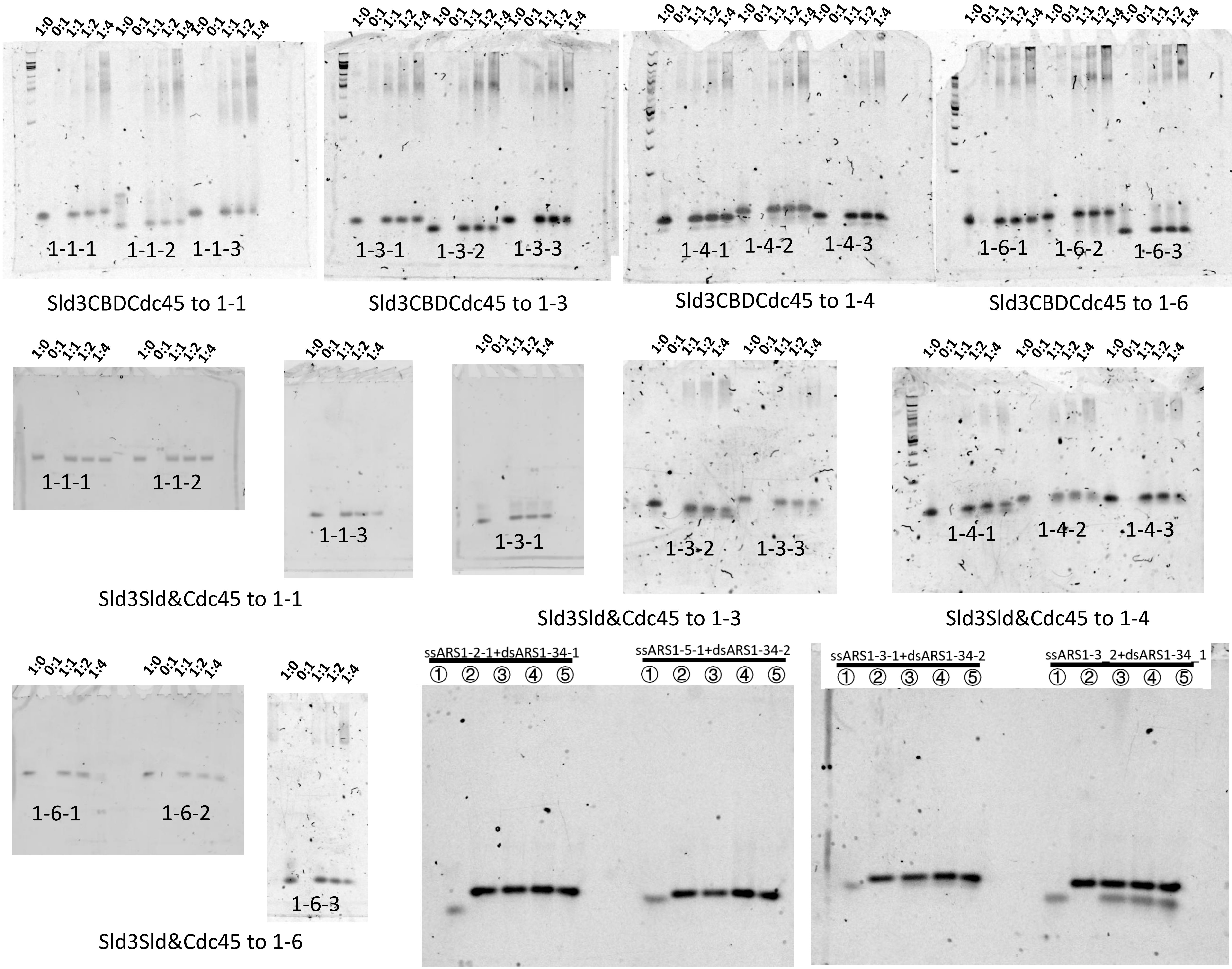

Figure 4-figure supplement 3, Source Data 1. Original native-PAGE corresponding to Figure 4-figure supplement 3. ssDNA was stained with SYBR safe. Due to replacement of imaging equipment, the images exhibit certain background differences, which do not affect the experimental conclusions.
