# Supplementary material for: Sld3CBD–Cdc45 structural insights into Cdc45 recruitment for CMG complex formation during DNA replication
Source: eLife. 2025 Sep 8;13:RP101717. doi: 10.7554/eLife.101717 (PMC12416888; doi:10.7554/eLife.101717)
Supplement: Supplementary file 1. [file elife-101717-supp1.docx]

**Supplementary file 1. Primers used in this study**

Sld3CBD-Cdc45

|  | **Forward primer** | **Reverse primer** |
| --- | --- | --- |
| **Sld3CBD** | 5’CTCGAGCACCACCACCACCACCACTGAG | 5’GGTATATCTCCTTCTTAAAGTTAAAGTTAAACAAAATTATTTCTAGAGG |
| **Cdc45** | 5’CTCGAGTCTGGTAAAGAAACCGCTG | 5’ATTTCGATTATGCGGCCGTGTACAATAC |

Sld7-Sld3ΔC-Cdc45

|  | **Forward primer** | **Reverse primer** |
| --- | --- | --- |
| **Sld7** | 5’GGAATTCCATATGCCGCTGTTTAAAGAAC | 5’CCGCTCGAGTTACGTTTTGGTGAACATTTC |
| **Sld3ΔC** | 5’CATGCCATGGAACCGAGCGAAG | 5’CCGCTCGAGGTTACCCTTCGGAACTTTG |
| **Cdc45** | 5’GGAATTCCATATGTATGGGTATCAACG | 5’CCGCTCGAGTTAAATCAGACCGCTCAGG |

Sld7-Sld3ΔC-Cdc45 IIS

|  | **Forward primer** | **Reverse primer** |
| --- | --- | --- |
| **Cdc45 IIS** | 5’ATTATCGAAATCCGCAAAGAAGATTCGCAGCCGTTCTCGGAACGTCTGACCC | 5’GGGTCAGACGTTCCGAGAACGGCTGCGAATCTTCTTTGCGGATTTCGATAAT |

Sld3CBD mutants

|  | **Forward primer** | **Reverse primer** |
| --- | --- | --- |
| **Sld3-3S** | 5’GACAGTAGTCTATCAAGTGAAACGCCCAAC | 5’CAAGCTGCATGCTCTATCTAAGTACAAGTCTAACTGTTC |
| **Sld3-3E** | 5’GACGAAGAACTATCAAGTGAAACGCCCAAC | 5’CAATTCGCATGCTCTATCTAAGTACAAGTCTAACTGTTC |
| **Sld3-Y** | 5’CCCAACCCAGATGCCATAGAAGCAT | 5’CGTTTCACTTGATAGTAGAATGTCCAAGTAGCATGCTCTATC |
| **Sld3-2R** | 5’ACTTACGTAGAGCATGCATCTTGGACATTCTACTATCAAGTG | 5’ACAAGCGTAACTGTTCACAATAATCCAAAGATGTTGTTATTCTC |

Cdc45 mutants

|  | **Forward primer** | **Reverse primer** |
| --- | --- | --- |
| **Cdc45-RA** | 5’GCAATTTATAGATTATGCGTCTTACAAGACGGACCC | 5’TAAATGCTTGATTAATTTCTTCTCCAATATAGCAACCC |
| **Cdc45-3E** | 5’ATAGAGAGTGCGAGTTACAAGACGGACCCGATTTAGACTTG | 5’AAATTCTCTCATGCTTGATTAATTTCTTCTCCAATATAGCAACCC |
| **Cdc45-2E** | 5’CATTCGAAGAGAAGCTGACCTTGAGTGGATTG | 5’GTGATTCATCTTCACGACGTATTTCAATTATGGAACTTTC |
| **Cdc45-3S** | 5’ATAGAAGTTGCTCATTACAAGACGGACCCGATTTAGACTTG | 5’AAATTCTTGAATGCTTGATTAATTTCTTCTCCAATATAGCAACCC |
| **Cdc45-2S** | 5’CATTCAGTGAGAAGCTGACCTTGAGTGGATTG | 5’GTGAACTATCTTCACGACGTATTTCAATTATGGAACTTTC |
| **Cdc45 W481R** | 5’GCTCAGTAGAGATGCTCTAGATGACAGAAAGGTGG | 5’ GAGCATCTCTACTGAGCCAAAAATTCGAAACC |
| **Cdc45 G367D** | 5’GCTAGAATGGATATACCATTAAGTACTGCACAAGAAACATG | 5’GGTATatcCATTCTAGCAAACATCTTATGCAATC |
