# Supplementary material for: Sld3CBD–Cdc45 structural insights into Cdc45 recruitment for CMG complex formation during DNA replication
Source: eLife. 2025 Sep 8;13:RP101717. doi: 10.7554/eLife.101717 (PMC12416888; doi:10.7554/eLife.101717)
Supplement: Supplementary file 2. [file elife-101717-supp2.docx]

**Supplementary** **file 2. Statistics of data collection and refinement**

CC_1/2_ (%) 99.7 (75.7)

No. unique reflection 30628 (4843)

Multiplicity 6.6 (6.5)

Structure refinement

R_work_ / R_free_ (%) 20.1 / 26.3

Statistics for the highest-resolution shell are shown in parentheses

Data collection

Resolution range(Å) 43.5 - 2.6 (2.7 - 2.6)

Cell dimension(Å) 70.8 107.6 128.3

Space group *P*2_1_2_1_2_1_

Number of unique reflections 30622 (3000)

Completeness (%) 99.9 (99.6)

Multiplicity 6.6 (6.5)

R_merge_ (%)^a^ 12.7 (97.1)

<I/sigma(I)> 13.3 (2.05)

CC_1/2_(%) 99.7 (75.7)

Number of Sld3CBD-Cdc45 in ASU 1

Wilson B factor 43.9

Refinement

Resolution range(Å) 43.5 - 2.6

R_free_/R_work_ (%)^b^ 26.2 / 21.9

Total number of atoms 6516

Protein atoms 6476

Water atoms 40

Others 0

Averaged B factor 55.0

RMS deviations

Bonds (Å) 0.0026

Angles (º) 0.63

Ramachandran plot (%)

　　 Favored 94.1

Allowed 4.62

Outliers　 　　　　　　　　 1.28

Favored 94.1

Allowed 4.62

Outliers 1.28

PDB code 8J09

^a^ $R_{merge}={\sum_{hkl} \sum_{i} \left| I_{i}\left( hkl \right)-\left\langle I_{i}\left( hkl \right) \right\rangle\right|}/{\sum_{hkl} \sum_{i} I_{i}\left( hkl \right)}$, where *i* is the number of observations of a given reflection and *I(hkl)* is the average intensity of the *i* observations.

^b^ $R={\sum\left| \left| F_{0} \right|-\left| F_{c} \right| \right|}/{\sum\left| F_{0} \right|}$. |$F_{0}$| and |$F_{c}$| are amplitudes of the observed and calculated structure factors, respectively. $R_{work}$is the R value for reflections used in the refinement, whereas $R_{free}$ is the R value for 5% of the reflections, which are selected in thin shells and are not included in the refinement.
